# Low-Temperature Dynamics at Nano- and Macroscales: Organic Crystal That Exhibits Low-Temperature Molecular Motion and the Thermosalient Effect

**DOI:** 10.1021/acs.chemmater.5c00171

**Published:** 2025-05-01

**Authors:** José L. Belmonte-Vázquez, Durga Prasad Karothu, Carl H. Fleischer, Dazaet Galicia-Badillo, Mauricio Maldonado-Domínguez, Robert W. Schurko, Liang Li, Panče Naumov, Braulio Rodríguez-Molina

**Affiliations:** † Instituto de Química (IQ), Universidad Nacional Autónoma de México (UNAM), Circuito Exterior s/n, Ciudad Universitaria, Coyoacán, Ciudad de México 04510, México; ‡ Departamento de Química Orgánica, Facultad de Química (FQ), Universidad Nacional Autónoma de México (UNAM), Ciudad Universitaria, Ciudad de México 04510, México; § Smart Materials Lab, New York University Abu Dhabi, PO Box 129188, Abu Dhabi, UAE; ∥ Department of Chemistry & Biochemistry, Florida State University, Tallahassee, Florida 32306, United States; ⊥ National High Magnetic Field Laboratory, Tallahassee, Florida 32310, United States; # Center for Smart Engineering Materials, New York University Abu Dhabi, PO Box 129188, Abu Dhabi UAE; ∇ Novel Materials Development Lab, Sorbonne University Abu Dhab, PO Box 38044, Abu Dhabi UAE; ○ Research Center for Environment and Materials, Macedonian Academy of Sciences and Arts, Bul. Krste Misirkov 2, Skopje MK−1000, Macedonia; ◆ Molecular Design Institute, Department of Chemistry, New York University, 100 Washington Square East, New York, New York 10003, United States

## Abstract

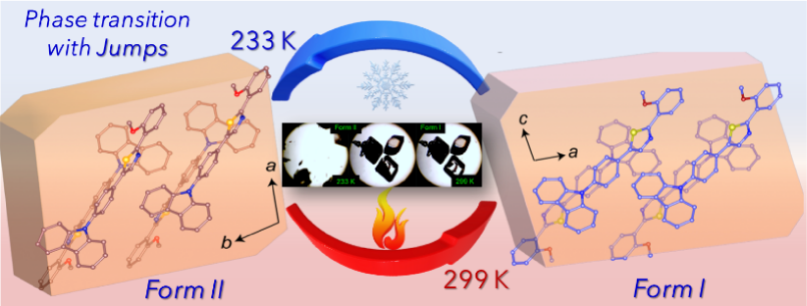

The thermosalient effect is a rarely observed, potentially very useful and at the present, unpredictable mechanical response during a phase transition that is thought to hold the potential for rapid and clean energy conversion devoid of gaseous products. Here, we report the serendipitous discovery of a rare instance of a thermosalient organic solid that exhibits the effect below room temperature. The crystals of this carbazole-based material are dynamic at both molecular and macroscopic scales. Using variable temperature synchrotron X-ray diffraction and variable-temperature solid-state nuclear magnetic resonance (ssNMR), we thoroughly examined the hysteretic structural transition in this material, emphasizing its macroscopic reconfigurability. We discovered unexpected large-amplitude molecular oscillations in the low-temperature phase, which challenge conventional assumptions about salient materials. Notably, we combined ^2^H ssNMR with computational modeling to reveal this dual-scale dynamism, setting the groundwork for advancements in energy-efficient actuators, sensors, and intelligent materials. This work might open new avenues for developing crystalline materials that can be implemented in innovative devices operating seamlessly across various scales.

## Introduction

The power to transform energy into motion has propelled societal development forward, shaping our progress and achievements throughout history. Motility, which is defined as the ability of living organisms and cells to move independently using energy, is central to their survival, behavior, adaptability, and evolution.^1,2^ Replicating such motions with artificial materials is at the forefront of materials research, with examples of both irreversible and reversible mechanisms being used to generate motility, whereby systems can advance in space in a controlled fashion. One of the most impressive and recently explored demonstrations of the dynamic nature of crystalline materials is the thermosalient effect that manifests as a sudden propulsion of some crystals when they cross over a thermal phase transition boundary due to heating or cooling, whereupon they reshape, explode, or leap over very long distances.^3,4^ There are more than two dozen materials known to display this mechanical effect, with a few more examples being added every year.^5−17^ It was later discovered that, in addition to heat, this effect can also be induced by light (photosalient effect)^18−27^ and pressure (mechanosalient effect).^28−47^ These so-called salient phenomena, which are the fastest known mechanical responses among a range of dynamic effects in molecular crystals,^48−51^ have recently attracted attention from the perspective of their potential for extremely rapid propulsion, disintegration, or actuation when they are induced in pure materials^52−60^ or in their composites with polymers.^61^ The low density of these organic materials, which translates into light weights and absence of metals, can undergo rapid transformations with motions that occur on time scales shorter than milliseconds, suggesting their use as prospective rapid organic actuators.^62^ Thermosalient crystals are rare, and in many ways, unique dynamic materials for being able to rapidly respond to heat (10^–3^–10^–1^ s) and efficiently generate and transfer momentum to other objects for actuation, thereby acting as fast single-stroke actuators. The ability to detect temperature changes, amplify molecular motion to a macroscopic scale, and respond with shapeshifting or ballistic events qualify these solids as the prime class of materials underpinning the emerging field of crystal adaptronics.^50^ Unlike typical high-energy materials that generate motion by releasing gaseous products, the salience-driven propulsion is considered “chemically clean” because it relies solely on a phase transition between two solids. Along these lines, recent efforts have been directed toward quantification of the performance of thermosalient solids as single-crystal actuators, and testing in devices such as solid fuses,^55^ microfluidic elements, or soft robots.^63−65^

Despite the increasing interest in the thermosalient effect, our understanding of its causes and mechanisms remains limited.^66^ Generally, the effect has been observed in relatively soft molecular crystals, which can store strain in a metastable structural phase before transforming. Analysis of the crystal structures suggests that certain structural requirements contribute to the effect, oftentimes including flat molecular shape, absence of strong intermolecular bonds, and/or a low-dimensional hydrogen bonding network.^49^ The mechanism is believed to be a martensitic transition—a nondisplacive, synchronous transformation that occurs with minimal structural changes.^67^ Recent studies have demonstrated that a low-frequency rotational mode can trigger the transition,^68,69^ and this event is preceded by softening of the crystal lattice.^42^ A Brillouin light scattering study showed that the transition is driven by elastic instability with considerable anharmonic intermolecular interaction associated with the molecular motions.^70^ Once initiated, the phase transition progresses very quickly,^71^ and the inability of the unit cell to absorb and dissipate the strain results in rapid deformation or disintegration; in the latter case, defects usually result in strong concentration of energy and subsequent catastrophic events with an explosive outcome. The advancement of the transition is sufficient to generate a shock wave that can be detected as acoustic signals.^72^ Detailed studies have established that the rate of progression of the phase front in thermosalient crystals is much faster than other phase transitions.^51,71^ However, a full explanation for these observations, and especially of the primary processes in these transitions has not been provided, thereby hampering rational design and improvement;^71^ a deeper understanding of the atomic-scale mechanism and related dynamics of the thermosalient transitions^66^ is essential for comparison and correlation with the respective crystal structures, assessment of the efficacy required for optimization in the actuator design, as well as for implementation in actual devices. It is worth noting that techniques such as low-frequency Raman or terahertz time-domain spectroscopies could be used to trace this kind of phenomenon as recently demonstrated.^73,74^ There is only one example to date where a thermosalient crystal is dynamic on a molecular scale, which is composed by a rotor and stator molecules.^36^ Many of these transitions result in partial or complete destruction of the crystal by splitting^3^ or explosion.^9^ In this study, we report and describe a new thermosalient crystalline solid that undergoes a significant and sudden change in size following a single-crystal-to-single-crystal transition while exerting a substantial, measurable force. The phase transition is reversible over several cooling and heating cycles, occurring rapidly with thermal hysteresis. This study contributes an example of a low-temperature thermosalient solid (233–243 K) built from a low symmetry molecule that remains rigid at room temperature, and unexpectedly, selected vibrational modes are activated at low temperature.

## Results and Discussion

### Characterization of the Phase Transition

**Figure 1:**
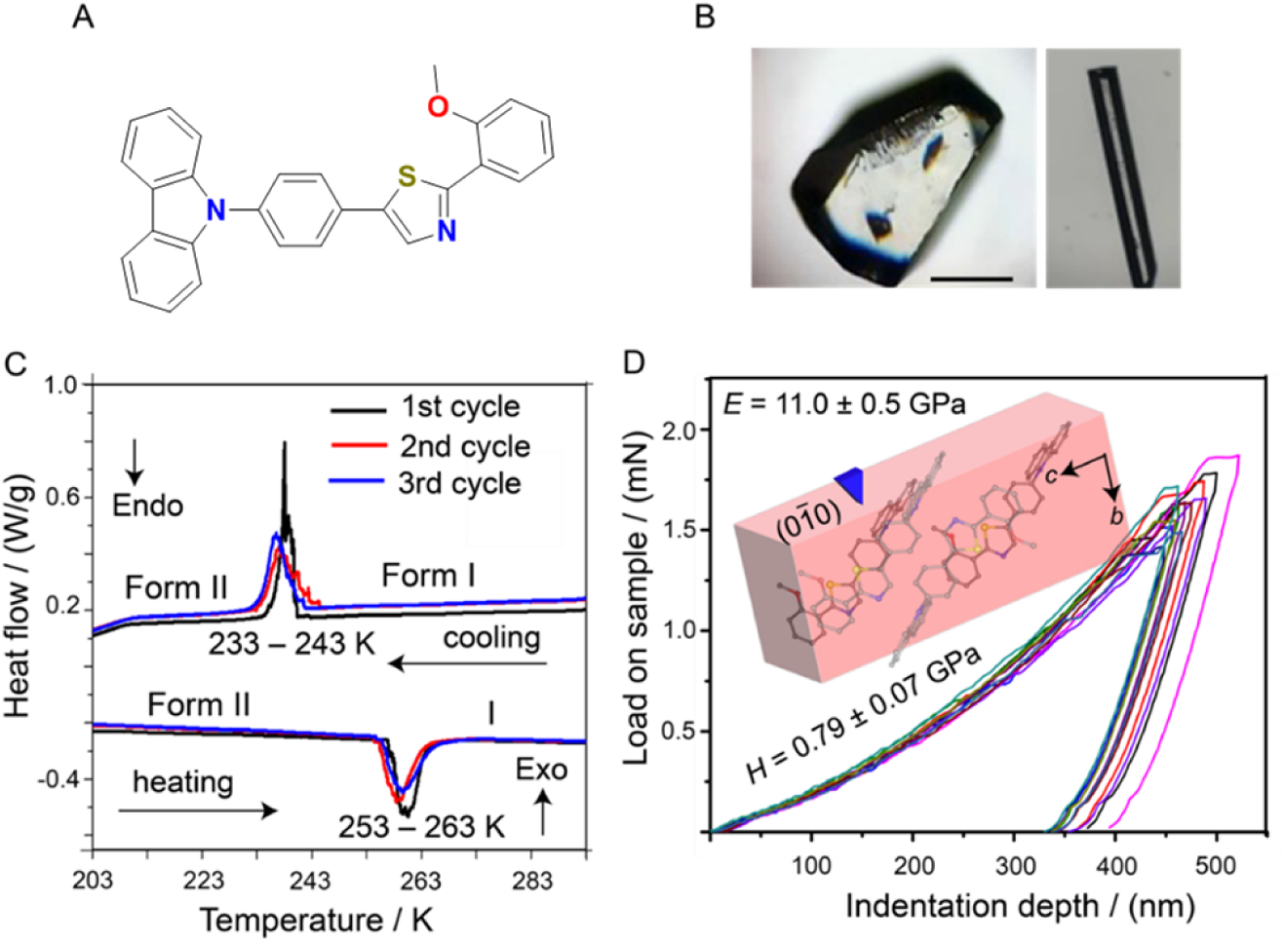
Phase transition and mechanical properties of form I crystals of **1**. (A) Molecular structure of compound **1**. (B) Optical microscopy images of two different habits of form I—irregular blocky and elongated crystals. (C) Thermal effects accompanying the transition between forms I and II recorded by DSC (three thermal cycles are shown). Note the small offset in the maximum transition temperature between the consecutive cycles. (D) Load–depth curves recorded for form I crystal on its (010) face at selected penetration depths. Young’s modulus (*E*) and hardness (*H*) are shown with the e.s.d.’s calculated from 36 indents performed at a single indentation depth.

Our studies into carbazole derivatives as potentially thermosalient compounds were motivated by the serendipitous discovery of a thermosalient amphidynamic cocrystal of carbazole and 1,4-diazabicyclo[2.2.2]octane (DABCO).^36^ Subsequent screening of other hydrogen-bonded cocrystals showed interesting phase transitions and fast rotational dynamics;^75−78^ however, no significant salient effects could be detected. Along these lines, we turned to derivatives of carbazole and prepared compound **1** (Figure 1A), using a modified synthetic protocol for thiazoles that was previously reported by some of us^79^ (for more detailed synthesis, see the Methods section in the Supporting Information). Elongated prismatic and block-shaped single crystals of compound **1** of good quality were obtained by slow solvent evaporation from a solution in a mixture of dichloromethane and hexanes at room temperature (Figure 1B). The crystals of **1** were assessed for thermal effects by using Differential Scanning Calorimetry (DSC). Single crystals of **1**(80) obtained at room temperature (form I) were found to undergo an exothermic phase transition on cooling to 233–243 K to a low-temperature phase (form II). Form II returns to the original form I upon heating via an endothermic transition at 253–263 K. The relatively sharp transition and significantly large thermal hysteresis of about 10 K suggest that the phase transition is of first order. The phase transition is sharp and reversible, with temperature shifts in the second and later thermal cycles similar to those reported for other thermosalient transitions (Figure 1C). The phase transition was observed during cooling of block-shaped crystals by using hot-stage microscopy, showing impressive thermosalient behavior with changes in the crystal dimensions (Video S1). During the phase transition, the crystals often exhibited splintering behavior, ejecting small pieces. Depending on their size and quality, the overall integrity of the crystals was maintained during the initial phase transition. However, during the transition from form I to form II, a slight reduction in transparency was observed, accompanied by the formation of defects and cracks. These defects became more pronounced with successive cooling and heating cycles (Figure S5). After two to three cycles, the structural integrity of the crystals as a single entity was compromised, resulting in fragmentation into smaller crystalline pieces. Notably, these smaller fragments continued to exhibit jumping behavior. It is worth noting that, based on synchrotron single-crystal X-ray diffraction data at variable temperature, a pair of CH···π interactions dominate the structure at 300 K, likely restricting the mobility of the phenylene ring. Upon cooling to 200 K, one CH···π interaction disappears due to crystal rearrangement, and a new π···π interaction emerges (Figure S6).

The accessible crystal face(s) of a crystal of form I, which were amenable for indentation, were matched with their Bravais-Friedel-Donnay-Harker (BFDH) morphology reconstructed from the crystal structure. The respective load–displacement curves for form I were obtained by performing indentation experiments at varying penetration depths. The respective indents were analyzed with atomic force microscopy (AFM), and the profiles were found to be smooth, without a pileup of material around the indent. The crystals of form I are relatively stiff and hard for organic crystal, with Young’s modulus for the (010)/(01̅0) pair *E* = 11.0 ± 0.5 GPa and *H* = 0.79 ± 0.07 GPa (Figure 1D). Due to experimental limitations and the unavailability of low-temperature indentation capability, a direct comparison of the mechanical properties between forms I and II could not be performed.

### Structural Characterization and the Mechanism of the Phase Transition

During the transition from form I to form II upon cooling, most of the block-shaped crystals jump off the base due to the sudden release of mechanical stress or strain associated with the phase change, and this phenomenon highlights the dynamic nature of the transformation process. The transition is fully reversible; when the crystals of form II are heated, they revert to form I, again accompanied by sudden self-propulsion. This reversible and rapid motion underscores the structural changes occurring during the phase transitions between these two forms. The propagation of the phase transition can be observed in real-time using an optical microscope as progression of the phase boundary during the cooling of a crystal to low temperature (Video S2).

**Figure 2:**
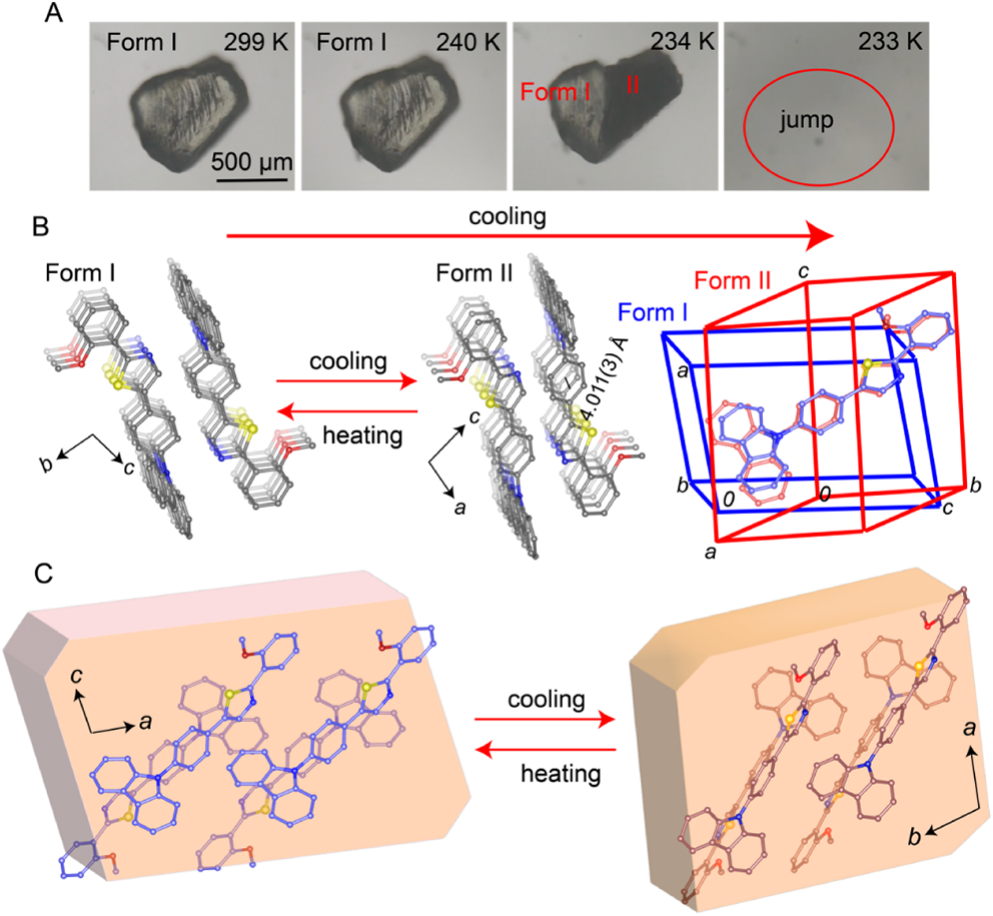
Optical and structural analysis of the thermosalient phase transition of **1**. (A) Optical micrographs showing a crystal of form I undergoing a thermosalient phase transition to form II during cooling. (B) Changes in the crystal packing during the phase transition are observable with the superimposition of the structures of forms I and II. (C) Mechanism of the phase transition depicting the molecular orientation in the two phases and the orientation of the unit cell axes.

To investigate the molecular mechanism of the observed phase transition, single crystal structure analyses of form I and form II were performed by using X-ray diffraction from crystals at 290 and 200 K, respectively. A crystal of form I was cooled to the phase transition temperature, where it transformed into the other form, and diffraction data were collected. The crystal of form I is in the triclinic space group *P*1̅ (*a* = 9.0482(7) Å, *b* = 11.2757(9) Å, *c* = 11.5271(9) Å, α = 93.629(3)°, β = 99.053(2)°, γ = 112.116(2)°), with one molecule in the asymmetric unit (Table S5). The molecules are arranged in a head-to-tail manner along the [100] crystallographic direction. They interact, though weak intermolecular interactions, with adjacent molecules along the [010] and [001] directions. The structure is dominated by π–π stacking and C–H···π interactions. Upon cooling, form II is obtained, and its structure is in the triclinic space group *P*1̅ with slightly different unit cell size (*a* = 9.3165(13) Å, *b* = 9.7354(12) Å, *c* = 11.7977(15) Å, α = 76.334(4)°, β = 85.051(4)°, γ = 87.674(4)°) and a single molecule in the asymmetric unit (Table S5). At low temperatures, the molecules are arranged in a head-to-tail manner via short π–π stacking interactions, with a stacking distance between two adjacent molecules of 4.073(3) Å (Figure 2B, Figure S6). The molecular arrangement in the crystal of form II remains similar to that of form I. Although the conformational change between the two phases is small—much like other cases involving minor structural differences related to a thermosalient phase transition^36,72^—this change is likely the primary contributor to the internal stress that leads to the phase transition (Figure 2C). The unit cell parameters recorded upon cooling and heating show hysteresis, in line with the thermal effects described above (Figure S3 and Table S1).

### Dynamics of the Phase Transition Studied by Solid-State NMR

**Figure 3:**
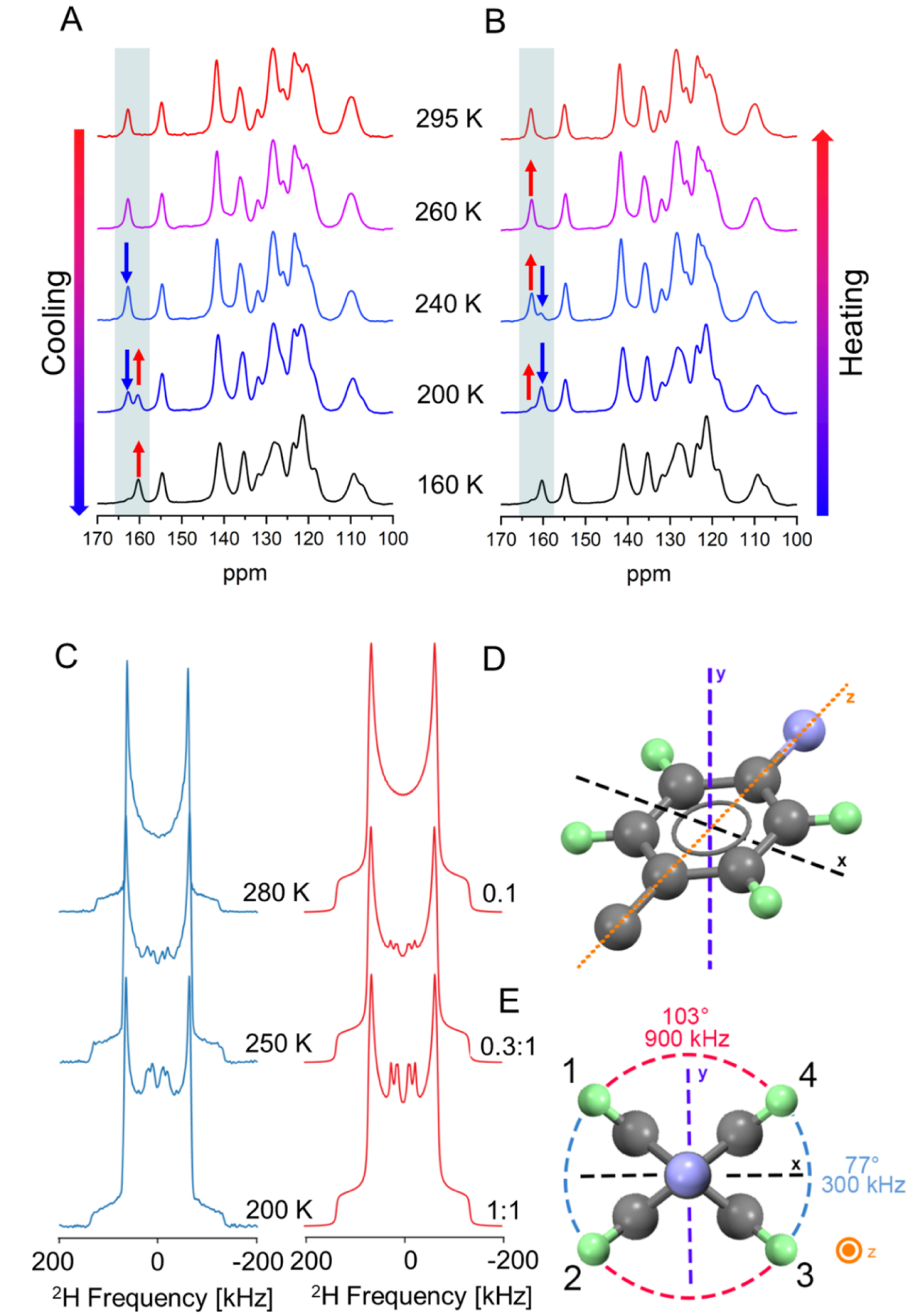
Variable-temperature ^1^H → ^13^C{^1^H} CP/MAS NMR spectra of compound **1** acquired at 14.1 T and ν_rot_ = 14 kHz as the sample was (A) cooled and then (B) heated. (C) ^2^H ssNMR spectra of compound **1** with experimental spectra (blue) as the sample was cooled and ^2^H ssNMR spectral simulations (red) simulated using a relative intensity ratio of the fast-motion-limit dynamics to the slow-motion-limit static ring, indicated at the bottom of the spectra. (D) The frame of reference for the rotational model, with the phenyl ring positioned in the *xz* plane with its 2-fold rotational axis along *z* (the deuterons are indicated in green). (E) A four-site exchange model and jump angles were used to simulate the VT ^2^H ssNMR spectra.

To increase our understanding of the salient transformation at the molecular level, we carried out a detailed study of the solid material by different solid-state NMR techniques. First, we focused on the changes that occur in the carbon atoms by using VT ^1^H → ^13^C CP/MAS experiments on compound **1**, by cooling a polycrystalline sample from 308 to 200 K, and then heating back up to ca. 308 K (Figure S1). There are well-resolved differences in peak positions and intensities over this temperature range between 158 and 165 ppm (Figure 3), a region with peaks corresponding to the 2-thiazole carbon atom. The new signal clearly corroborates the phase transition and confirms the new conformation of the central moiety within the new phase. On cooling the sample (Figure 3A), a 2-thiazole carbon peak is observed at 162.8 ppm; however, at 240 K and below, a second peak is observed at 160.3 ppm. As the temperature decreases, the peaks at 162.8 and 160.3 ppm diminish and grow in intensity, respectively; however, even at 200 K, a very small peak at 162.8 ppm is still observed. Thus, it seems possible that a small amount of form I still exists at that temperature.

By heating the sample from 200 to 308 K (Figure 3B), the opposite occurs i.e., the peaks at 162.8 and 160.3 ppm increase and decrease in intensity, respectively, indicating a return to the original phase. However, the relative intensities of the peaks during the heating process differ from those during cooling—this fact, as well as the presence of a low-intensity peak at 160.3 ppm at 308 K during heating, corroborates the hysteresis of the phase transition described above by DSC measurements (Figure 1C) and VT SCXRD (Figure S2).

To obtain further information about the local mobility of other molecular components before, after, and during the phase transition, we synthesized the deuterated derivative **1**-*d*_4_, which showed a similar salient phase transition as its parent compound (Figure S3). The isotopically enriched compound is amenable to VT ^2^H static ssNMR analysis and provides information on the behavior of the phenyl ring directly attached to the carbazole. For the deuterated compound, the measurements were performed on cooling from 295.5 to 200 K and on heating back to 295.5 K (Figure S4A,B, respectively). A broad Pake-like doublet was observed between room temperature and 280 K (Figure S4A). The line shape can be simulated with *C*_Q_ = 167 kHz and η_Q_ = 0.05, values typical for deuterons involved in rigid sp^2^ C–D bonds. Therefore, no apparent motion-related effects on the spectrum are observed at this temperature, indicating that the phenyl ring is stationary at this temperature range initially explored. As the temperature was further decreased, two features in the spectra were observed: a persistent Pake doublet that gradually grew in breadth, such that at 200 K, it was simulated with *C*_Q_ = 180 kHz and η_Q_ = 0.05, and a set of sharper peaks that gradually emerge, likely from a ^2^H pattern of lower intensity.

The inner peaks (Figure 3C) are counterintuitive because they indicate a motion of the phenyl ring. The Pake doublet that gradually expanded in width suggests the persistence of form I, even at low temperatures. The new set of sharper peaks was attributed to an underlying powder pattern corresponding to form II, in which the phenyl rings undergo temperature-dependent motion. The impact of the motion of the phenylene on the ^2^H NMR spectra can be simulated with a four-site exchange model with two of the deuterons in the phenyl ring related by reflection or 2-fold rotation (Figure 3D,E). At 200 K, the deuteron in site 1 (positioned at 38.5° above the *x*-axis) exchanges positions with sites 2 and 4 via ring flips of 77° and 103°, which occur at frequency rates of 300 kHz and 900 kHz, respectively. The observations of the persistent form I and the emergence of form II are consistent with the single crystal X-ray diffraction and ^13^C VT SSNMR data. There are two possible explanations for the relatively low integrated intensities of the low-temperature signal. First, the rate of ring flipping is on the order of the breadth of the powder pattern, which results in diminished integrated intensity in spectra acquired with the quadrupolar echo pulse sequence.^81^ Second, only a small fraction of the molecules in the low-temperature phase are undergoing ring flips, with the majority remaining stationary, thereby giving rise to a Pake-like doublet similar to that corresponding to the room-temperature phase. The former explanation appears to be more likely, as evidenced by the diminished overall intensities of the spectra (and increased noise) at intermediate temperatures (Figure S4). As the sample is heated from 200 to 295.5 K, the second set of sharp peaks diminishes in intensity, whereas that of the Pake-like doublet increases. However, the second set of peaks persists up to 295.5 K, supporting again the hysteresis in the phase transition that is consistent with the VT ^13^C NMR data, as discussed above.

### Computational Thermodynamics of the Thermosalient Transition

To further scrutinize the mechanism of the thermosalient transition, we employed classical molecular dynamics (MD) simulations. For this purpose, 2 × 2 × 2 supercells containing 16 molecules each were constructed and thermally equilibrated at *T* = 250 K and *T* = 300 K. These temperatures were selected using the X-ray synchrotron data acquired upon heating, starting from 150 K, where form II is still persistent at 250 K due to the hysteresis of the phase transition, as evidenced by the crystallographic parameters compiled in Table S1. All simulations were carried out under constant pressure *p* = 1 atm, and the equilibrium dynamics were simulated for 20 ns (after a 1 ns pre-equilibration run) in each case (for details, see the Materials and Methods section). As seen in Table 1, average densities obtained from MD simulations at thermal equilibrium lie within a 6% absolute error from the values estimated from our XRD analysis, supporting the applicability of the selected Molecular Mechanics (MM) protocol to model the structure-dependent properties of the system under study.

**Table 1:** Comparison of the Density of Forms I and II of Crystalline **1**, Estimated from XRD Experiments (ρ_XRD_) and Derived from 20 ns Trajectories Produced under Isothermal-Isobaric Classical MD (ρ_MD_) with the Dreiding Force Field

Form	*T* (K)	ρ_XRD_ (g/cm^3^)	ρ_MD_ (g/cm^3^)	Error (%)
I	300	1.322	1.258	–4.8
II	250	1.393	1.306	–6.2

With the selected computational method (Supporting Information) we employed a thermodynamic model to analyze forms I and II. At thermal equilibrium, the thermosalient response of crystalline **1** upon cooling implies that the Gibbs energy change of formation of forms I and II (Δ*G*_f1_ and Δ*G*_f2_) at 300 and 250 K must follow the relations:
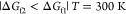
1
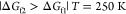
2

This implies that form I is thermodynamically favored over form II at 300 K, and lowering of the temperature triggers the thermosalient event, generating form II, which is now favored over form I. Since Δ*G* = Δ*H* – *T*Δ*S*, let us first focus on the enthalpic component of the transition. Our DSC analyses reveal that, during cooling, the transition from form I to form II is exothermic. Therefore, enthalpies of formation of forms I and II (Δ*H*_f1_ and Δ*H*_f2_) follow:
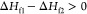
3

We can estimate the enthalpy change of the transition, Δ*H*, starting from the thermodynamic equivalence 4:
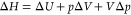
4

Since the thermosalient event occurs at constant atmospheric pressure, the term *V*Δ*p* = 0 kcal/mol. The *p*Δ*V* term can be calculated from the change in molar volumes of the unit cells, obtained either from XRD or MD simulations. The magnitude of the *p*Δ*V* term for forms I and II is *p*Δ*V* < 0.02 kcal/mol, i.e., it is so low that we can neglect its influence on the transition, leading to the approximation in 5:
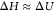
5

Approximation 5 is convenient since a reasonable estimation of Δ*U* can be made by calculating the total electronic potential energy of formation, for each unit cell in its equilibrium geometry, Δ*E*, using a quantum chemical method. For this purpose, we employed density functional theory, DFT, with the *meta*-GGA functional M06-L and the triple-ζ basis TNP.^82^
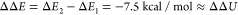
6

The favorable ΔΔ*E* = −7.5 kcal/mol term agrees with a rigidization of the crystal structure by the establishment of stronger and/or more abundant intermolecular contacts. This same energy difference corresponds to the change in *lattice energy* of both solids from the thermosalient event. Regarding the entropy change for the process, the contrast of the structural disorder in form II vs the ordered form I suggests that low-frequency degrees of freedom might activate upon cooling. That is, some components of the total entropy change *increase* upon cooling. Estimating the *T*Δ*S* term requires knowledge of all possible microstates available for forms I and II in equilibrium, and their relative energies, which is generally unfeasible. In crystalline solids, the entropy is determined exclusively by vibrational degrees of freedom, since translational and rotational motions of the constituent units are suppressed by the long-range periodic order. Accordingly, the entropy term in the free energy expression, *T*Δ*S*, can be entirely attributed to vibrational dynamics. This vibrational entropy accounts for all quantized normal modes within the lattice, including low-frequency motions that may resemble hindered rotations. Therefore, the entropy contribution to the free energy can be reduced to *TS* = *TS*_vib_.

The *S*_vib_ term can be estimated computationally using either classical or quantum mechanical approaches. We calculated *S*_vib_ values classically, for 2 × 2 × 2 supercells of forms I and II, using eq 7); all values are contained in Table S2.
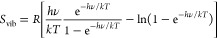
7

**Figure 4:**
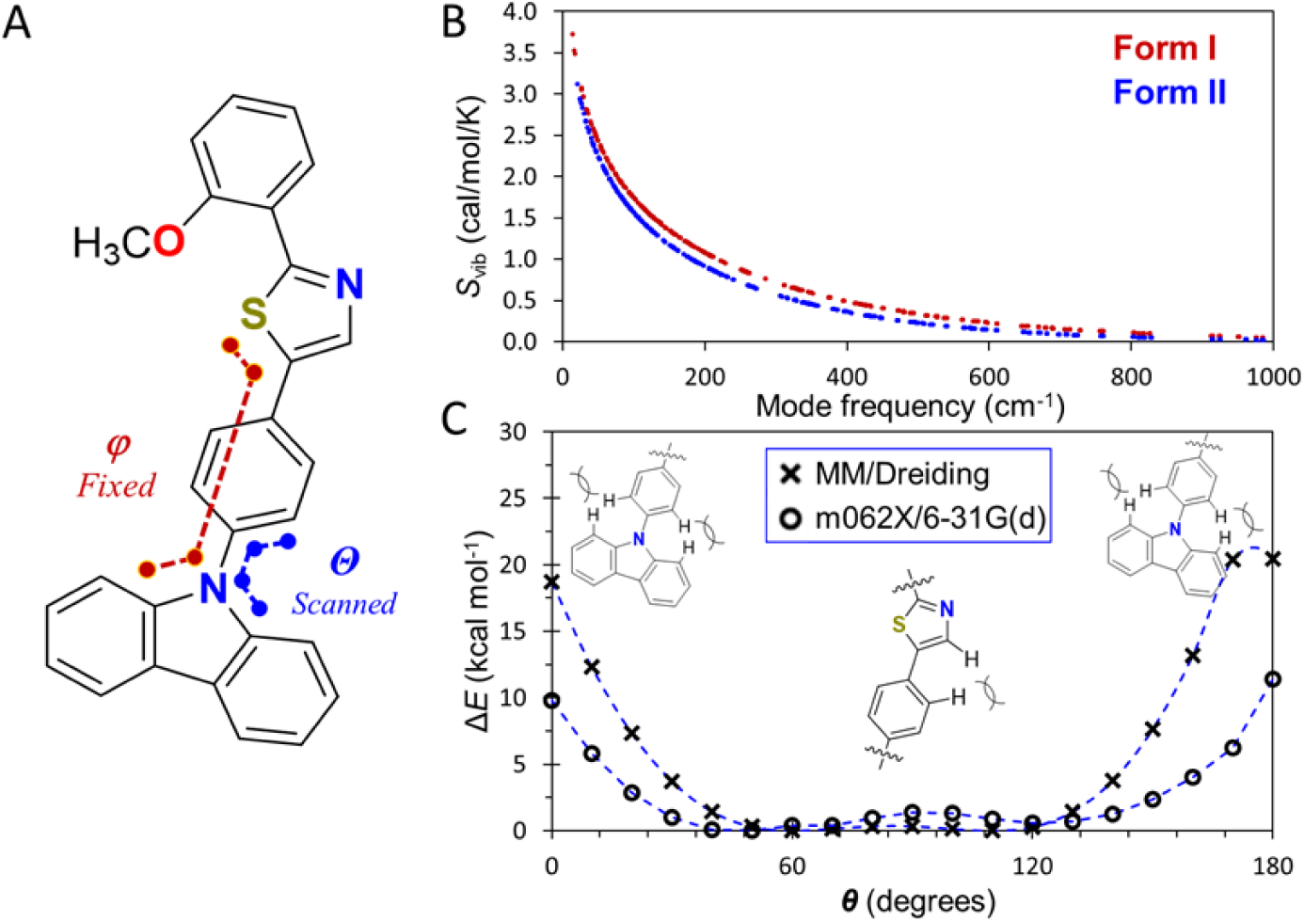
(A) Molecular structure of **1**, highlighting the relevant dihedral angles that were frozen (magenta) and scanned (blue) in our computational analysis. (B) Vibrational contribution to the entropy of formation, *S*_vib_, calculated from harmonic vibrational frequencies using the MM protocol on forms I (red) and II (blue) of compound **1**. (C) Unidimensional rotational scans for the active phenylene ring in molecule **1** calculated using molecular mechanics (crosses) and density functional theory (circles). All the optimized geometries and numerical energy values are included in the Supporting Information.

Equation 8 diverges artifactually at vanishingly low frequencies, and a common approach to alleviate this pitfall is taking a threshold frequency of 50 cm^–1^ as a lower limit to its applicability.^83^ Using this approximation, we show in Figure 4B the entropy profiles for the vibrational modes of forms I and II in the 50 cm^–1^ < Δ*Ê*_MD_ < 1000 cm^–1^ range. Summation over this range yields *S*_vib_ values of 330.3 J/K and 258.1 J/K for forms I and II, respectively, suggesting the intuitive notion that the higher temperature form embodies the most significant entropy term.

While classical thermodynamics provides an average view of the thermosalient phenomenon at hand, it does not reveal the origin neither it characterizes the disorder in the phenylene rings in form I, active in the submegahertz frequency range. To gain molecular-level resolution, we investigated the rotation of the disordered phenylene ring computationally, by decomposing the energy barrier to the hindered rotation of the active ring into two contributions, namely (a) the inherent barrier for rotation of the isolated molecular unit in the gas phase (identical for forms I and II), calculated using DFT, and (b) the environmental component to this barrier, characteristic to each system and calculated using classical MD at constant pressure and temperature. The torsional scans for compound **1** (Figure 4A) calculated using the Dreiding MM protocol and the M062X density functional with the def2TZVP basis set are presented in Figure 4C.

The results in Figure 4C reveal that the MM protocol qualitatively predicts the torsional profile, yielding two minima separated by a shallow barrier and two dominant maxima when the phenylene rings approach coplanarity with the carbazole fragment. However, the steric penalty of the near-coplanar conformations is largely overestimated for the isolated molecular unit. For the ensuing analysis (*vide infra*), DFT-derived values will be employed.

**Figure 5:**
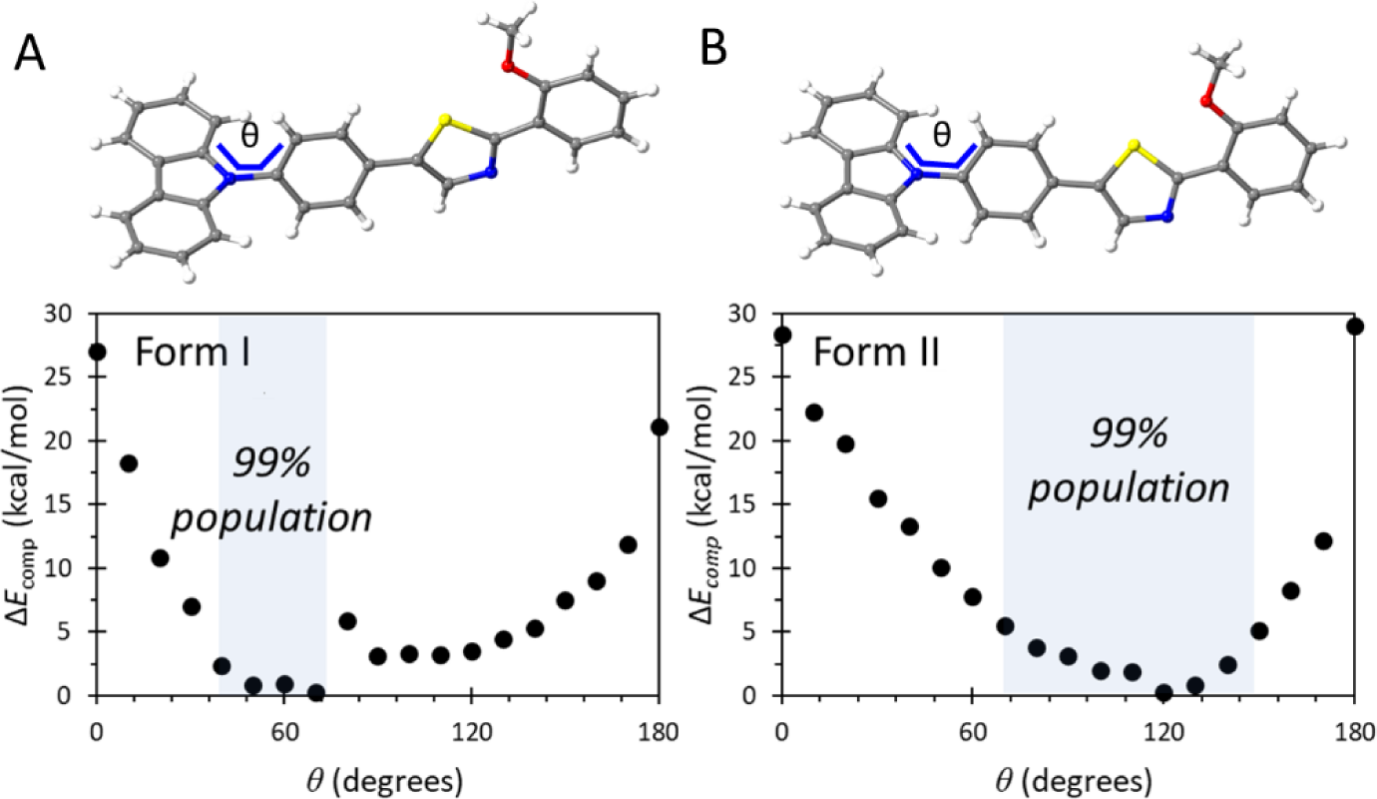
Energy profile and phase transition at high temperature (A) and low temperature (B) of compound **1**.

Regarding the crystal lattice effect on the torsional profile, we scanned the θ angle (Figure 4A) using classical molecular dynamics, imposing a harmonic constraint on the active angle while keeping all other degrees of freedom unconstrained. The MD-derived average energy values, Δ*Ê*_MD_, were employed to estimate a composite energy term (8), Δ*E*_comp_, defined as
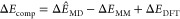
8where the lattice effects are approximated by subtraction of the MM-derived gas-phase potential energy values (Δ*E*_MM_) from the Δ*Ê*_MD_ terms, and the molecular-level effects are accounted for at the DFT level of theory. The resulting torsional profiles are presented in Figure 5A,B.

Figure 5 shows that the low-temperature form II features a relatively shallow potential energy basin, enabling the phenylene ring to explore a torsional range of approximately 80° at the available thermal energy, with a Boltzmann-estimated population of 99% within this space. In contrast, the high-temperature form I presents a narrower well, restricting the accessible torsional space to about 30–40° for 99% of the population. In both phases, the barriers to full 180° rotation remain high, consistent with VT ^2^H ssNMR experiments, which suggest large-amplitude vibrations rather than full phenylene flipping. These findings offer a coherent picture that aligns both the macroscopic thermodynamic observations, where the total entropy is higher in form I, and the microscopic behavior of the system.

Notably, although the total vibrational entropy decreases with cooling, the amplitude of motion along specific internal coordinates, such as the phenylene torsion, can increase due to subtle alterations in intermolecular interactions and crystal packing (Figure S6). This reflects a local enhancement in dynamic freedom along soft, low-frequency modes, driven by the softening of the potential energy surface. Such behavior exemplifies how, in exceptional materials like this one, densification at lower temperatures may simultaneously favor stronger noncovalent contacts and facilitate internal flexibility, permitting increased local motion despite a global reduction in entropy.

## Conclusions

In this report, we describe the synthesis and detailed characterization of a new organic crystal exhibiting a thermosalient effect at low temperature. We provide a detailed account of the mentioned features by VT SCXRD, DSC, nanoindentation, VT ^1^H → ^13^C CP MAS NMR, and VT ^2^H quadrupolar-echo spin ssNMR, as well as computational thermodynamics. The organic crystal undergoes a single-crystal-to-single-crystal transition at low temperature (233–243 K) with low molecular symmetry, resulting in mechanical motion in response to thermal stimuli. This phase transition is observable over multiple cooling and heating cycles with thermal hysteresis. The key features that distinguish this crystal include a discrete thermosalient effect, low molecular symmetry, notable stiffness at room temperature, and the emergence of enhanced local dynamics upon cooling, arising from a softening of the potential energy surface along a low-frequency internal coordinate. While the total vibrational entropy decreases with temperature, the amplitude of motion along specific modes increases—an unusual and previously unreported feature in thermosalient materials. These insights into the entropic and mechanical subtleties of the transition open new avenues for designing dynamic molecular crystals with futuristic functional applications in advanced soft materials, tailored actuators, artificial muscles, or microrobotic systems.

## Materials and Methods

All chemicals were purchased from Sigma-Aldrich and used as received. Solvents were dried using molecular sieves. The purification steps were performed using flash column chromatography using SiO_2_ gel 70–230 nm mesh, hexane, ethyl acetate, and tetrahydrofuran as eluents. ^1^H and ^13^C spectra were recorded in deuterochloroform (CDCl_3_, 7.26 ppm for ^1^H and 77.00 ppm for ^13^C) using Bruker AV 400 and 300 MHz spectrometers. Data are reported in the following order: the chemical shift in parts per million (ppm), multiplicities s (singlet), d (doublet), t (triplet), m (multiplet), coupling constants, *J* (hertz), and integration. FT-Infrared spectra were recorded on a Bruker ATR in a 450–4000 cm^–1^ range. High-resolution mass spectra were recorded on a JEOL AccuTOF JMS-T100LC mass spectrometer by Direct Analysis in Real Time (DART). Melting points were determined using a Fisher-Johns melting points apparatus (uncorrected). X-ray data collection was performed with VT using a Bruker Apex-Duo, Bruker Smart Apex II.

### Synthesis and Crystallization

More details of the synthesis of individual components are shown in Scheme S1.

#### Compound **1**

To an oven-dried thick-walled reaction tube equipped with a stirrer bar 9-(4-ethynylphenyl)-9*H*-carbazole (250 mg, 0.93 mmol, 1.0 equiv), mesylazide (118.0 mg, 0.98 mmol, 1.05 equiv), Copper(I) thiophene-2-carboxylate (CuTC) (17.8 mg, 0.093 mmol, 0.1 equiv), molecular sieves 4 Å (80 mg), and CHCl_3_ (20 mL) were added. After the tube was closed the reaction was stirrer at room temperature for 24 h. Then, under N_2_, Rh_2_(OCO^*t*^Bu)_4_ (11.4 mg, 0.019 mmol, 0.02 equiv) and *O*-methyl 2-methoxybenzothioate (341 mg, 1.9 mmol, 2.0 equiv) were added to the reaction mixture. The reaction mixture was heated at 70 °C for 2 h, cooled to room temperature, and silica gel (Aldrich, 4 g) was added. The reaction mixture was stirred at room temperature for 4 h. After this, the crude material was passed through a short pad of Na_2_SO_4_ (anhydrous) and eluted with ethyl acetate. The filtrate was concentrated under reduced pressure, adsorbed on SiO_2_, and purified by flash column chromatography using hexanes:THF (9:1). The desired product **1** (266 mg, 0.6 mmol, 68%) was obtained as a colorless and translucent crystalline solid. Crystals were obtained by slow evaporation using a DCM/hexane mixture. TLC (10% AcOEt/hexanes, *R*_f_ = 0.17); mp (204–206 °C). ^1^H NMR (500 MHz, CDCl_3_) δ 8.54 (d, *J* = 7.4 Hz, 1H), 8.21 (s, 1H), 8.16 (d, *J* = 7.7 Hz, 2H), 7.88 (d, *J* = 8.2 Hz, 2H), 7.65 (d, *J* = 8.1 Hz, 2H), 7.50–7.41 (m, 5H), 7.34–7.28 (m, 2H), 7.17 (t, *J* = 7.5 Hz, 1H), 7.09 (d, *J* = 8.2 Hz, 1H), 4.11 (s, 3H). ^13^C NMR (126 MHz, CDCl_3_) δ 162.1, 156.6, 140.8, 138.7, 137.8, 136.8, 135.0, 131.7, 130.7, 128.5, 128.1, 127.8, 126.2, 123.7, 121.6, 120.5, 120.3, 111.6, 109.9, 55.9. IR (λ_max_, cm^–1^) ν: 3045, 2938, 2833, 2039, 1880, 1660, 1526, 1596, 1583, 1530, 1499, 1479, 1450, 1413. HRMS (EI^+^) calculated for C_28_H_20_N_2_OS [M + H]^+^ 432.1296, found 432.1294.

#### Compound **1-*d***_**4**_

To an oven-dried thick-walled reaction tube equipped with a stirrer bar 9-(4-ethynylphenyl-2,3,5,6-*d*_4_)-9*H*-carbazole (250 mg, 0.92 mmol, 1.0 equiv), mesylazide (111.6 mg, 0.92 mmol, 1.0 equiv), Copper(I) thiophene-2-carboxylate (CuTC) (17.6 mg, 0.092 mmol, 0.1 equiv), molecular sieves 4 Å (80 mg), and CHCl_3_ (20 mL) were added. After the tube was closed, the reaction was stirred at room temperature for 24 h. Then, under N_2_, Rh_2_(OCO^*t*^Bu)_4_ (11.2 mg, 0.018 mmol, 0.02 equiv) and *O*-methyl 2-methoxybenzothioate (335.8 mg, 1.84 mmol, 2.0 equiv) were added to the reaction mixture. After that, the reaction mixture was heated at 70 °C for 2 h. cooled to room temperature, and silica gel (Aldrich, 4 g) was added. The reaction mixture was stirred at room temperature for 4 h. Then, the crude material was passed through a short pad of Na_2_SO_4_ (anhydrous) and eluted with ethyl acetate. The filtrate was concentrated under reduced pressure, adsorbed on SiO_2_ and purified by flash column chromatography using hexane:THF (9:1). The desired product (175 mg, 0.401 mmol, 43.5%) was obtained as a yellow crystalline solid. Crystals were obtained by slow evaporation using a DCM/hexane mixture. ^1^H NMR (500 MHz, CDCl_3_) δ 8.46 (dd, *J* = 7.8, 1.6 Hz, 1H), 8.17 (d, *J* = 2.8 Hz, 2H), 8.15 (s, 1H), 7.50–7.41 (m, 5H), 7.34–7.27 (m, 2H), 7.17 – 7.11 (m, 1H), 7.08 (d, *J* = 8.3 Hz, 1H), 4.10 (s, 3H). ^13^C NMR (126 MHz, CDCl_3_) δ 162.0, 156.5, 140.8, 138.7, 137.6, 137.0, 136.5, 131.6, 130.5, 128.4, 128.0–127.0 (m), 126.2, 123.7, 121.5, 120.5, 120.3, 111.6, 109.9, 55.9. HRMS (ESI^+^) calculated for C_28_H_17_D_4_N_2_OS [M + H]^+^ 437.16257, found 437.16157.

#### Compound **4**

A bottom flask equipped with a stir bar was charged with methyl 2-methoxybenzoate (1000 mg, 6.0 mmol, 1.0 equiv), Lawesson reagent (2434 mg, 6.0 mmol, 2.0 equiv) and *p*-xylene (30 mL). Then, the reaction mixture was stirred at reflux. After 48 h of reaction, the crude was cooled to room temperature, passed through a silica gel pad, and washed with hexanes. The filtrate was adsorbed on SiO_2_ and purified using hexanes as eluent to obtain a red-orange liquid (689 mg, 3.8 mmol, 63%). TLC (10% AcOEt/hexanes, *R*_f_ = 0.35). ^1^H RMN (400 MHz, CDCl_3_) δ: 7.66 (d, *J* = 7.7 Hz, 1H), 7.39 (t, *J* = 7.9 Hz, 1H), 6.97 – 6.91 (m, 2H), 4.26 (s, 3H), 3.85 (s, 3H). ^13^C RMN (101 MHz, CDCl_3_) δ: 214.9, 156.1, 132.2, 131.6, 129.0, 120.4, 112.0, 59.5, 56.2. HRMS (IE^+^) *m*/*z*: [C_9_H_10_O_2_S]^+^, calculated: 182.0402, found: 182.0403.

## Supporting Information Available

Experimental details: spectroscopic characterization, differential scanning calorimetry (DSC), microscopy, X-ray crystal structure analysis, nanoindentation, solid-state NMR spectroscopy, and molecular mechanics—dynamics calculations (PDF)
The phase transition observed during cooling of block-shaped crystals by using hot-stage microscopy, showing impressive thermosalient behavior with changes in the crystal dimensions (Video S1) (MP4)
The propagation of the phase transition observed in real time using an optical microscope as progression of the phase boundary during the cooling of a crystal to low temperature (Video S2) (MP4)
(CIF)
(CIF)
(CIF)
(CIF)
(CIF)
(CIF)
(CIF)
(CIF)
(CIF)
(CIF)
(CIF)

## Supplementary Material

cm5c00171_si_003.mp4

cm5c00171_si_004.cif

cm5c00171_si_005.cif

cm5c00171_si_006.cif

cm5c00171_si_007.cif

cm5c00171_si_008.cif

cm5c00171_si_009.cif

cm5c00171_si_010.cif

cm5c00171_si_011.cif

cm5c00171_si_012.cif

cm5c00171_si_013.cif

cm5c00171_si_014.cif

## References

[ref1] Berg H. C. Dynamic properties of bacterial flagellar motors. Nature 1974, 249, 77–79. 10.1038/249077a0. 4598032

[ref2] Miyata M.; Robinson R. C.; Uyeda T. Q. P.; Fukumori Y.; Fukushima S.-I.; Haruta S.; Homma M.; Inaba K.; Ito M.; Kaito C.; Kato K.; Kenri T.; Kinosita Y.; Kojima S.; Minamino T.; Mori H.; Nakamura S.; Nakane D.; Nakayama K.; Nishiyama M.; Shibata S.; Shimabukuro K.; Tamakoshi M.; Taoka A.; Tashiro Y.; Tulum I.; Wada H.; Wakabayashi K.-I. Tree of motility – A proposed history of motility systems in the tree of life. Genes Cells 2020, 25, 6–21. 10.1111/gtc.12737. 31957229 PMC7004002

[ref3] Centore R.; Jazbinsek M.; Tuzi A.; Roviello A.; Capobianco A.; Peluso A. A series of compounds forming polar crystals and showing single-crystal-to-single-crystal transitions between polar phases. CrystEngComm 2012, 14, 2645–2653. 10.1039/c2ce06352b.

[ref4] Skoko Ž.; Zamir S.; Naumov P.; Bernstein J. The thermosalient phenomenon. “Jumping crystals” and crystal chemistry of the anticholinergic agent oxitropium bromide. J. Am. Chem. Soc. 2010, 132, 14191–14202. 10.1021/ja105508b. 20860383

[ref5] George G. C.; Kruse S. J.; Forbes T. Z.; Hutchins K. M. Off-the-shelf thermosalience of anthracene-9-thiocarboxamide. Chem. Commun. 2024, 60, 7697–7700. 10.1039/D4CC01765J. 38828748

[ref6] Takeda T.; Ozawa M.; Akutagawa T. Jumping crystal of a hydrogen-bonded organic framework induced by the collective molecular motion of a twisted π system. Angew. Chem. Int. Ed. 2019, 58, 10345–10352. 10.1002/anie.201905075. 31106500

[ref7] Chen Y.; Zhang J.; Zhang J.; Wan X. Directional crystal jumping controlled by chirality. J. Am. Chem. Soc. 2024, 146, 9679–9687. 10.1021/jacs.3c13416. 38478888

[ref8] Rath B. B.; Gallo G.; Dinnebier R. E.; Vittal J. J. Reversible thermosalience in a one-dimensional coordination polymer preceded by anisotropic thermal expansion and the shape memory effect. J. Am. Chem. Soc. 2021, 143, 2088–2096. 10.1021/jacs.0c12363. 33476147

[ref9] Omoto K.; Nakae T.; Nishio M.; Yamanoi Y.; Kasai H.; Nishibori E.; Mashimo T.; Seki T.; Ito H.; Nakamura K.; Kobayashi N.; Nakayama N.; Goto H.; Nishihara H. Thermosalience in macrocycle-based soft crystals via anisotropic deformation of disilanyl architecture. J. Am. Chem. Soc. 2020, 142, 12651–12657. 10.1021/jacs.0c03643. 32571018

[ref10] Uddin M. A.; Martín R.; Gámez-Valenzuela S.; Echeverri M.; Delgado M. C. R.; Puebla E. G.; Monge A.; Gómez-Lor B. Giant thermosalient effect in a molecular single crystal: dynamic transformations and mechanistic insights. J. Am. Chem. Soc. 2024, 146, 27690–27700. 10.1021/jacs.4c09222. 39315726 PMC11467902

[ref11] Kato K.; Seki T.; Ito H. (9-Isocyanoanthracene)gold(I) complexes exhibiting two modes of crystal jumps by different structure change mechanisms. Inorg. Chem. 2021, 60, 10849–10856. 10.1021/acs.inorgchem.1c00881. 33886301

[ref12] Chen Y.; Li J.; Gong J. Jumping crystal based on an organic charge transfer complex with reversible on/off switching of luminescence by external thermal stimuli. ACS Mater. Lett. 2021, 3, 275–281. 10.1021/acsmaterialslett.0c00575.

[ref13] Takazawa K.; Inoue J.; Matsushita Y. Repeatable actuations of organic single crystal fibers driven by thermosalient-phase-transition-induced buckling. Small 2022, 18 (41), 2204500 10.1002/smll.202204500. 36084217

[ref14] Takazawa K.; Inoue J. I.; Mitsuishi K.; Yoshida Y.; Kishida H.; Tinnemans P.; Engelkamp H.; Christianen P. Phase-transition-induced jumping, bending, and wriggling of single crystal nanofibers of coronene. Sci. Rep. 2021, 11 (1), 3175 10.1038/s41598-021-82703-5. 33542387 PMC7862269

[ref15] Miura Y.; Takeda T.; Yoshioka N.; Akutagawa T. Thermosalient effect of 5-fluorobenzoyl-4-(4-methoxyphenyl)ethynyl-1-methylimidazole without phase transition. Cryst. Growth Des. 2022, 22, 5904–5911. 10.1021/acs.cgd.2c00547.

[ref16] Hu X.; Xiao Y.; Qi L.; Bai Y.; Sun Y.; Ye Y.; Xie C. An attempt to design thermosalient crystals by co-crystallization: the twisted angle between aromatic rings. Crystals 2023, 13, 701 10.3390/cryst13040701.

[ref17] Angeloski A.; Price J. R.; Ennis C.; Smith K.; McDonagh A. M.; Dowd A.; Thomas P.; Cortie M.; Appadoo D.; Bhadbhade M. Thermosalience revealed on the atomic scale: rapid synchrotron techniques uncover molecular motion preceding crystal jumping. Cryst. Growth Des. 2022, 22, 1951–1959. 10.1021/acs.cgd.1c01495.

[ref18] Naumov P.; Sahoo S. C.; Zakharov B. A.; Boldyreva E. V. Dynamic single crystals: kinematic analysis of photoinduced crystal jumping (the photosalient effect). Angew. Chem. Int. Ed. 2013, 52, 9990–9995. 10.1002/anie.201303757. 23873664

[ref19] Commins P.; Natarajan A.; Tsai C.-K.; Khan S. I.; Nath N. K.; Naumov P.; Garcia-Garibay M. A. Structure–reactivity correlations and mechanistic understanding of the photorearrangement and photosalient effect of α-santonin and its derivatives in solutions, crystals, and nanocrystalline suspensions. Cryst. Growth Des. 2015, 15, 1983–1990. 10.1021/acs.cgd.5b00135.

[ref20] Sahoo S. C.; Nath N. K.; Zhang L.; Semreen M. H.; Al-Tel T. H.; Naumov P. Actuation based on thermo/photosalient effect: a biogenic smart hybrid driven by light and heat. RSC Adv. 2014, 4, 7640–7647. 10.1039/C3RA46688D.

[ref21] Medishetty R.; Husain A.; Bai Z.; Runčevski T.; Dinnebier R. E.; Naumov P.; Vittal J. J. Single crystals popping under UV light: a photosalient effect triggered by a [2 + 2] cycloaddition reaction. Angew. Chem., Int. Ed. 2014, 53, 5907–5911. 10.1002/anie.201402040. 24664890

[ref22] Medishetty R.; Sahoo S. C.; Mulijanto C. E.; Naumov P.; Vittal J. J. Photosalient behavior of photoreactive crystals. Chem. Mater. 2015, 27, 1821–1829. 10.1021/acs.chemmater.5b00021.

[ref23] Hatano E.; Morimoto M.; Imai T.; Hyodo K.; Fujimoto A.; Nishimura R.; Sekine A.; Yasuda N.; Yokojima S.; Nakamura S.; Uchida K. Photosalient phenomena that mimic impatiens are observed in hollow crystals of diarylethene with a perfluorocyclohexene ring. Angew. Chem. Int. Ed. 2017, 56, 12576–12580. 10.1002/anie.201706684. 28834074

[ref24] Yadava K.; Gallo G.; Bette S.; Mulijanto C. E.; Karothu D. P.; Park I. H.; Medishetty R.; Naumov P.; Dinnebier R. E.; Vittal J. J. Extraordinary anisotropic thermal expansion in photosalient crystals. IUCrJ 2020, 7, 83–89. 10.1107/S2052252519014581. PMC6949593 31949907

[ref25] Dutta B.; Sinha C.; Mir M. H. The sunlight-driven photosalient effect of a 1D coordination polymer and the release of an elusive cyclobutane derivative. Chem. Commun. 2019, 55, 11049–11051. 10.1039/C9CC06016B. 31453613

[ref26] Hasebe S.; Hagiwara Y.; Komiya J.; Ryu M.; Fujisawa H.; Morikawa J.; Katayama T.; Yamanaka D.; Furube A.; Sato H.; Asahi T.; Koshima H. Photothermally driven high-speed crystal actuation and its simulation. J. Am. Chem. Soc. 2021, 143, 8866–8877. 10.1021/jacs.1c03588. 34096298

[ref27] Tahir I.; Ahmed E.; Karothu D. P.; Fsehaye F.; Halabi J. M.; Naumov P. Intersectional effects of crystal features on the actuation performance of dynamic molecular crystals. J. Am. Chem. Soc. 2024, 146, 30174–30182. 10.1021/jacs.4c08320. 39392877 PMC11544611

[ref28] Etter M. C.; Siedle A. R. Solid-state rearrangement of (phenylazophenyl)palladium hexafluoroacetylacetonate. J. Am. Chem. Soc. 1983, 105, 641–643. 10.1021/ja00341a065.

[ref29] Steiner T.; Hinrichs W.; Saenger W.; Gigg R. ‘Jumping crystals’: X-ray structures of the three crystalline phases of (±)-3,4-di-O-acetyl-1,2,5,6-tetra-*O*-benzyl-myo-inositol. Acta Crystallogr. 1993, B49, 708–718. 10.1107/S0108768193000795.

[ref30] Panda M. K.; Runčevski T.; Sahoo S. C.; Belik A. A.; Nath N. K.; Dinnebier R. E.; Naumov P. Colossal positive and negative thermal expansion and thermosalient effect in a pentamorphic organometallic martensite. Nat. Commun. 2014, 5 (1), 4811 10.1038/ncomms5811. 25185949

[ref31] Hagiwara H.; Konomura S. Thermosalience coupled to abrupt spin crossover with dynamic ligand motion in an iron(II) molecular crystal. CrystEngcomm 2022, 24, 4224–4234. 10.1039/D2CE00501H.

[ref32] Karothu D. P.; Naumov P. Thermosalience of 1,2,4,5-tetrachlorobenzene. Isr. J. Chem. 2021, 61, 557–562. 10.1002/ijch.202100056.

[ref33] Vicente A. I.; Joseph A.; Ferreiro L. P.; Carvalho M. D.; Rodrigues V. H. N.; Duttine M.; Diogo H. P.; Minas da Piedade M. E.; Calhorda M. J.; Martinho P. N. Dynamic spin interchange in a tridentate Fe(III) Schiff-base compound. Chem. Sci. 2016, 7, 4251–4258. 10.1039/C5SC04577K. 30155072 PMC6013817

[ref34] Kikuchi Y.; Matsumoto S. Chlorinated diketopyrrolopyrrole dye exhibits a three-step polymorphic transition with thermosalient effects. CrystEngComm 2021, 23, 5882–5890. 10.1039/D1CE00454A.

[ref35] Hean D.; Alde L. G.; Wolf M. O. Photosalient and thermosalient crystalline hemithioindigo-anthracene based isomeric photoswitches. J. Mater. Chem. C 2021, 9, 6789–6795. 10.1039/D1TC01358K.

[ref36] Colin-Molina A.; Karothu D. P.; Jellen M. J.; Toscano R. A.; García-Garibay M. A.; Naumov P.; Rodríguez-Molina B. Thermosalient amphidynamic molecular machines: motion at the molecular and macroscopic scales. Matter 2019, 1, 1033–1046. 10.1016/j.matt.2019.06.018.

[ref37] Sahoo S. C.; Sinha S. B.; Kiran M. S. R. N.; Ramamurty U.; Dericioglu A. F.; Reddy C. M.; Naumov P. Kinematic and mechanical profile of the self-actuation of thermosalient crystal twins of 1,2,4,5-tetrabromobenzene: a molecular crystalline analogue of a bimetallic strip. J. Am. Chem. Soc. 2013, 135, 13843–13850. 10.1021/ja4056323. 23895677

[ref38] Takahashi Y.; Kondo T.; Yokokura S.; Takehisa M.; Harada J.; Inabe T.; Matsushita M. M.; Awaga K. Electric and thermosalient properties of a charge-transfer complex exhibiting a minor valence instability transition. Cryst. Growth Des. 2020, 20, 4758–4763. 10.1021/acs.cgd.0c00516.

[ref39] Seki T.; Mashimo T.; Ito H. Anisotropic strain release in a thermosalient crystal: correlation between the microscopic orientation of molecular rearrangements and the macroscopic mechanical motion. Chem. Sci. 2019, 10, 4185–4191. 10.1039/C8SC05563G. 31057747 PMC6471989

[ref40] Karothu D. P.; Weston J.; Desta I. T.; Naumov P. Shape-memory and self-healing effects in mechanosalient molecular crystals. J. Am. Chem. Soc. 2016, 138, 13298–13306. 10.1021/jacs.6b07406. 27618207

[ref41] So H.-S.; Minami T.; Jindo T.; Matsumoto S. Thermosalient effect of two polymorphs of a diketopyrrolopyrrole dye with different crystal systems and molecular arrangements. CrystEngComm 2018, 20, 5317–5320. 10.1039/C8CE00940F.

[ref42] Zakharov B. A.; Michalchuk A. L.; Morrison C. A.; Boldyreva E. V. Anisotropic lattice softening near the structural phase transition in the thermosalient crystal 1,2,4,5-tetrabromobenzene. Phys. Chem. Chem. Phys. 2018, 20, 8523–8532. 10.1039/C7CP08609A. 29537423

[ref43] Tamboli M. I.; Karothu D. P.; Shashidhar M. S.; Gonnade R. G.; Naumov P. Effect of crystal packing on the thermosalient effect of the pincer-type diester naphthalene-2,3-diyl-bis(4-fluorobenzoate): a new class II thermosalient solid. Chem. Eur. J. 2018, 24, 4133–4139. 10.1002/chem.201705586. 29315895

[ref44] Lončarić I.; Popović J.; Despoja V.; Burazer S.; Grgičević I.; Popović D.; Skoko Ž. Reversible thermosalient effect of *N*′-2-propylidene-4-hydroxybenzohydrazide accompanied by an immense negative compressibility: structural and theoretical arguments aiming toward the elucidation of jumping phenomenon. Cryst. Growth Des. 2017, 17, 4445–4453. 10.1021/acs.cgd.7b00785.

[ref45] Mittapalli S.; Perumalla D.; Nangia A. Mechanochemical synthesis of *N*-salicylideneaniline: thermosalient effect of polymorphic crystals. IUCrJ 2017, 4, 243–250. 10.1107/S2052252517004043. PMC5414398 28512571

[ref46] Werny M. J.; Vittal J. J. Regulating thermosalient behaviour in three polymorphs. IUCrJ 2017, 4, 202–203. 10.1107/S2052252517005577. PMC5414393 28512566

[ref47] Nakawawa Y.; Morimoto M.; Yasuda N.; Hyodo K.; Yokojima S.; Nakamura S.; Uchida K. Photosalient effect of diarylethene crystals of thiazoyl and thienyl derivatives. Eur. J. Chem. 2019, 25, 7874–7880. 10.1002/chem.201900811. 30934138

[ref48] Panda M. K.; Runčevski T.; Husain A.; Dinnebier R. E.; Naumov P. Perpetually self-propelling chiral single crystals. J. Am. Chem. Soc. 2015, 137, 1895–1902. 10.1021/ja5111927. 25581716

[ref49] Naumov P.; Chizhik S.; Panda M. K.; Nath N. K.; Boldyreva E. Mechanically responsive molecular crystals. Chem. Rev. 2015, 115, 12440–12490. 10.1021/acs.chemrev.5b00398. 26535606

[ref50] Catalano L.; Naumov P. Exploiting rotational motion in molecular crystals. CrystEngComm 2018, 20, 5872–5883. 10.1039/C8CE00420J.

[ref51] Naumov P.; Karothu P.; Ahmed E.; Catalano L.; Commins P.; Halabi J. M.; Al-Handawi M. B.; Li L. The rise of the dynamic crystals. J. Am. Chem. Soc. 2020, 142, 13256–13272. 10.1021/jacs.0c05440. 32559073

[ref52] Commins P.; Desta I. T.; Karothu D. P.; Panda M. K.; Naumov P. Crystals on the move: mechanical effects in dynamic solids. Chem. Commun. 2016, 52, 13941–13954. 10.1039/C6CC06235K. 27711296

[ref53] Duan Y.; Semin S.; Tinnemans P.; Cuppen H.; Xu J.; Rasing T. Robust thermoelastic microactuator based on an organic molecular crystal. Nat. Commun. 2019, 10 (1), 4573 10.1038/s41467-019-12601-y. 31594954 PMC6783412

[ref54] Li L.; Commins P.; Al-Handawi M. B.; Karothu D. P.; Halabi J. M.; Schramm S.; Weston J.; Rezgui R.; Naumov P. Martensitic organic crystals as soft actuators. Chem. Sci. 2019, 10, 7327–7332. 10.1039/C9SC02444A. 31768232 PMC6837004

[ref55] Khalil A.; Ahmed E.; Naumov P. Metal-coated thermosalient crystals as electrical fuses. Chem. Commun. 2017, 53, 8470–8473. 10.1039/C7CC04251E. 28703252

[ref56] Dharmarwardana M.; Pakhira S.; Welch R. P.; Caicedo-Narvaez C.; Luzuriaga M. A.; Arimilli B. S.; McCandless G. T.; Fahimi B.; Mendoza-Cortes J. L.; Gassensmith J. J. Rapidly reversible organic crystalline switch for conversion of heat into mechanical energy. J. Am. Chem. Soc. 2021, 143, 5951–5957. 10.1021/jacs.1c01549. 33822596

[ref57] Lin J.; Guo Z.; Zhang K.; Zhao P.; Wu S.; Xu J.; Gong J.; Bao Y. Mechanical motion and modulation of thermal-actuation properties in a robust organic molecular crystal actuator. Adv. Funct. Mater. 2022, 32, 2203004 10.1002/adfm.202203004.

[ref58] Davies D. W.; Seo B.; Park S. K.; Shiring S. B.; Chung H.; Kafle P.; Yuan D.; Strzalka J. W.; Weber R.; Zhu X.; Savoie B. M.; Diao Y. Unraveling two distinct polymorph transition mechanisms in one n-type single crystal for dynamic electronics. Nat. Commun. 2023, 14 (1), 1304 10.1038/s41467-023-36871-9. 36944642 PMC10030468

[ref59] Hou I. C.-Y.; Li L.; Zhang H.; Naumov P. Smart molecular crystal switches. Smart Mol. 2024, 2, e20230031 10.1002/smo.20230031. PMC12118287 40625528

[ref60] Halabi J. M.; Séguy I.; Salvagnac L.; Leïchlé T.; Saya D.; Mathieu F.; Duployer B.; Karothu D. P.; Nicu L.; Naumov P. Microelectromechanical devices driven by thermosalient effects. Cell Rep. Phys. Sci. 2022, 3, 101133 10.1016/j.xcrp.2022.101133.

[ref61] Pantuso E.; Ahmed E.; Fontananova E.; Brunetti A.; Tahir I.; Karothu D. P.; Alnaji N. A.; Dushaq G.; Rasras M.; Naumov P.; Di Profio G. Smart dynamic hybrid membranes with self-cleaning capability. Nat. Commun. 2023, 14 (1), 5751 10.1038/s41467-023-41446-9. 37717049 PMC10505219

[ref62] Hussain M.; Jull E. I. L.; Mandle R. J.; Raistrick T.; Hine P. J.; Gleeson H. F. Liquid crystal elastomers for biological applications. Nanomaterials 2021, 11, 813 10.3390/nano11030813. 33810173 PMC8005174

[ref63] Lan L.; Li L.; Di Q.; Yang X.; Liu X.; Naumov P.; Zhang H. organic single-crystal actuators and waveguides that operate at low temperatures. Adv. Mater. 2022, 34, 2200471 10.1002/adma.202200471. 35104918

[ref64] Xiao Y.-Y.; Jiang Z.-C.; Zhao Y. Liquid crystal polymer-based soft robots. Adv. Intell. Syst. 2020, 2, 2000148 10.1002/aisy.202000148.

[ref65] Zhu C.; Lu Y.; Jiang L.; Yu Y. Liquid crystal soft actuators and robots toward mixed reality. Adv. Funct. Mater. 2021, 31, 2009835 10.1002/adfm.202009835.

[ref66] Ghasemlou S.; Li X.; Galimberti D. R.; Nikitin T.; Fausto R.; Xu J.; Holleman S.; Rasing T.; Cuppen H. M. Identifying and controlling the order parameter for ultrafast photoinduced phase transitions in thermosalient materials. Proc. Natl. Acad. Sci. U. S. A. 2024, 121, e2408366121 10.1073/pnas.2408366121. 39499639 PMC11573639

[ref67] Pond R. C.; Ma X.; Chai Y. W.; Hirth J. P. Topological modelling of martensitic transformations. In Dislocations in solids; Elsevier, 2007, Vol. 13.

[ref68] Park S. K.; Sun H.; Chung H.; Patel B. B.; Zhang F.; Davies D. W.; Woods T. J.; Zhao K.; Diao Y. Super- and ferroelastic organic semiconductors for ultraflexible single-crystal electronics. Angew. Chem. Int. Ed. 2020, 59, 13004–13012. 10.1002/anie.202004083. 32342626

[ref69] Yao Z.-S.; Mito M.; Kamachi T.; Shiota Y.; Yoshizawa K.; Azuma N.; Miyazaki Y.; Takahashi K.; Zhang K.; Nakanishi T.; Kang S.; Kanegawa S.; Sato O. Molecular motor-driven abrupt anisotropic shape change in a single crystal of a Ni complex. Nat. Chem. 2014, 6, 1079–1083. 10.1038/nchem.2092. 25411886

[ref70] Ko J.-H.; Lee K.-S.; Sahoo S. C.; Naumov P. Isomorphous phase transition of 1,2,4,5-tetrabromobenzene jumping crystals studied by Brillouin light scattering. Solid State Commun. 2013, 173, 46–50. 10.1016/j.ssc.2013.08.021.

[ref71] Ahmed E.; Karothu P.; Slimani A.; Halabi J. M.; Tahir I.; Canales K. Q.; Naumov P. Ultrafast, light, soft martensitic materials. Adv. Funct. Mater. 2022, 32, 2112117 10.1002/adfm.202112117.

[ref72] Panda M. K.; Etter M.; Dinnebier R. E.; Naumov P. Acoustic emission from organic martensites. Angew. Chem. Int. Ed. 2017, 56, 8104–8109. 10.1002/anie.201702359. 28544194

[ref73] Catalano L.; Sharma R.; Karothu D. P.; Saccone M.; Elishav O.; Chen C.; Juneja N.; Volpi M.; Jouclas R.; Chen H.-Y.; Liu J.; Liu G.; Gopi E.; Ruzié C.; Klimis N.; Kennedy A. R.; Vanderlick T. K.; McCulloch I.; Ruggiero M. T.; Naumov P.; Schweicher G.; Yaffe O.; Geerts Y. H. Toward on-demand polymorphic transitions of organic crystals via side chain and lattice dynamics engineering. J. Am. Chem. Soc. 2024, 146, 31911–31919. 10.1021/jacs.4c11289. 39514686 PMC11583316

[ref74] Juneja N.; Hastings J.; Stoll W. B.; Brennessel W. W.; Zarrella S.; Sornberger P.; Catalano L.; Korter T. M.; Ruggiero M. T. Fundamentally intertwined: anharmonic intermolecular interactions dictate both thermal expansion and terahertz lattice dynamics in molecular crystals. Chem. Commun. 2024, 60, 12169–12172. 10.1039/D4CC03307H. 39297177

[ref75] Li M.; Pal A.; Aghakhani A.; Pena-Francesch A.; Sitti M. Soft actuators for real-world applications. Nat. Rev. Mater. 2022, 7, 235–249. 10.1038/s41578-021-00389-7. 35474944 PMC7612659

[ref76] Colin-Molina A.; Jellen M. J.; Rodríguez-Hernández J.; Cifuentes-Quintal E.; Barroso J.; Toscano R. A.; Merino G.; Rodríguez-Molina B. Hydrogen-bonded crystalline molecular machines with ultrafast rotation and displacive phase transitions. Chem. Eur. J. 2020, 26, 11727–11733. 10.1002/chem.202001156. 32243632

[ref77] Colin-Molina A.; Velázquez-Chávez D.; Jellen M. J.; Rodríguez-Cortés L. A.; Cifuentes-Quintal M. E.; Merino G.; Rodríguez-Molina B. Dynamic characterization of crystalline fluorophores with conformationally flexible tetrahydrocarbazole frameworks. CrystEngComm 2020, 22, 3789–3796. 10.1039/D0CE00423E.

[ref78] Navarro-Huerta A.; Jellen M. J.; Arcudía J.; Teat S. J.; Toscano R. A.; Merino G.; Rodríguez-Molina B. Tailoring the cavities of hydrogen-bonded amphidynamic crystals using weak contacts: towards faster molecular machines. Chem. Sci. 2021, 12, 2181–2188. 10.1039/D0SC05899H. PMC8179257 34163983

[ref79] Belmonte-Vázquez J. L.; Hernández-Morales E. A.; Hernández F.; García-González M. C.; Miranda L. D.; Crespo-Otero R.; Rodríguez-Molina B. Asymmetric dual-state emitters featuring thiazole acceptors. Eur. J. Org. Chem. 2022, 2022, e202200372 10.1002/ejoc.202200372.

[ref80] Deposition numbers 2406302 (form I – 300K), 2406299 (form I – 250 K), 2406298 (form II – 200 K), 2406297 (form II – 150 K), 2406303 (form II – 200(2) K), 2406301 (form II – 250 K), 2406300 (form I – 300 K), 2406296 (form I – 350 K), 2406385 (form I – 290 K), 2406386 (form II – 200 K), and 2406424 (form II – 290 K), contain the supplementary crystallographic data for this paper. These data are provided free of charge by the joint Cambridge Crystallographic Data Centre and Fachinformationszentrum Karlsruhe Access Structures service.

[ref81] Larsen F. H. Simulations of molecular dynamics in solid-state NMR spectra of spin-1 nuclei including effects of CSA- and EFG-terms up to second order. Solid State Nucl. Magn. Reson. 2007, 31, 100–114. 10.1016/j.ssnmr.2007.02.004. 17418539

[ref82] Delley B. Ground-state enthalpies: evaluation of electronic structure approaches with emphasis on the density functional method. J. Phys. Chem. A 2006, 110, 13632–13639. 10.1021/jp0653611. 17165892

[ref83] Pracht P.; Grimme S. Calculation of absolute molecular entropies and heat capacities made simple. Chem. Sci. 2021, 12, 6551–6568. 10.1039/D1SC00621E. 34040731 PMC8139639

