# Supplementary material for: Low-Temperature Dynamics at Nano- and Macroscales: Organic Crystal That Exhibits Low-Temperature Molecular Motion and the Thermosalient Effect
Source: Chem Mater. 2025 May 1;37(9):3373–83. doi: 10.1021/acs.chemmater.5c00171 (PMC12079796; doi:10.1021/acs.chemmater.5c00171)
Supplement: Supplementary file 1 — cm5c00171_si_001.pdf [file cm5c00171_si_001.pdf]

SUPPORTING INFORMATION

Low-Temperature Dynamics at Nano- and Macro-Scales: Organic Crystal that Exhibits Low-Temperature Molecular Motion and Thermosalient Effect

José L. Belmonte-Vázquez,[1,2] Durga Prasad Karothu,[3] Carl. H. Fleischer III,[4,5] Dazaet Galicia-Badillo,[1] Mauricio Maldonado-Domínguez,[2] Robert W. Schurko,[4,5] Liang Li,[3,7] Panče Naumov,*[3,6,8,9] Braulio Rodríguez-Molina*[1]

[1]*Instituto de Química (IQ), Universidad Nacional Autónoma de México (UNAM), Circuito Exterior s/n, Ciudad Universitaria, Coyoacán, Ciudad de México 04510, México*

[2]*Departamento de Química Orgánica, Facultad de Química (FQ), Universidad Nacional Autónoma de México (UNAM), Ciudad Universitaria, Ciudad de México 04510, México*

[3]*Smart Materials Lab, New York University Abu Dhabi, PO Box 129188, Abu Dhabi, UAE*

[4]*Department of Chemistry & Biochemistry, Florida State University, Tallahassee, FL 32306*

[5]*National High Magnetic Field Laboratory, Tallahassee, FL 32310*

[6]*Center for Smart Engineering Materials, New York University Abu Dhabi, PO Box 129188, Abu Dhabi, UAE*

[7]*Novel Materials Development Lab, Sorbonne University Abu Dhab, PO Box 38044, Abu Dhabi, UAE*

[8]*Research Center for Environment and Materials, Macedonian Academy of Sciences and Arts, Bul. Krste Misirkov 2, MK–1000 Skopje, Macedonia*

[9]*Molecular Design Institute, Department of Chemistry, New York University, 100 Washington Square East, New York, NY 10003, USA*

**Corresponding authors: pance.naumov@nyu.edu (P.N.); brodriguez@iquimica.unam.mx (B. R.-M.)*

Materials and Methods

Synthesis and crystallization

The syntheses of compounds **2**, **2-*d₄***, **3**, and **4** were carried out using the methodologies previously reported by our research group.[1,2]

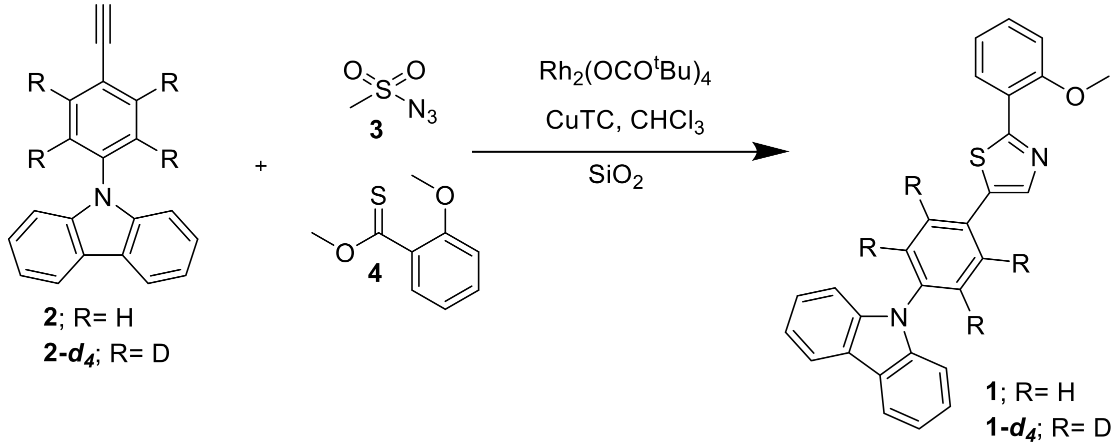

Scheme S1. Synthetic route to prepare compounds **1** and **1-*d₄***.

Differential scanning calorimetry. Differential scanning calorimetry (DSC) experiments were performed on a TA DSC-Q2000 instrument by using weighed samples (~ 1000–2000 mg) placed in aluminum crucibles without crippling the lid and heated from room temperature to the selected temperature at different rates of heating and cooling.

Microscopy. The phase transition of form I crystals during heating and cooling was observed by using optical microscope (Q32634 Q-imaging microscope, Linkam) equipped with a temperature-controlled heating/cooling stage (THMS600-PS).

Single crystal X-ray diffraction. Single crystal X-ray diffraction data for forms I and II of compound **1** and **1-*d₄*** were collected using XRD. Data were obtained at variable temperatures either on a Bruker D8 diffractometer equipped with a PHOTON II CPAD detector with synchrotron radiation (λ = 0.7288 Å) on beamline 12.2.1 at Advanced Light Source or from a Bruker Smart APEX II CCD with graphite monochromatic MoK_α radiation (λ = 0.71073 Å). Cell refinement was carried out using SAINT V8.38A. Structure solution, final refinement, and data output were carried out using SHELX-2014 through direct methods.

Alternatively, a Bruker APEX DUO diffractometer equipped with a Photon II detector and a Cobra cooling device (Oxford Cryosystems) was employed. Monochromated MoK_α radiation (λ = 0.71073 Å) was used. Data collection, integration, scaling, and

absorption corrections were carried out with the Bruker Apex 3 software.[3] The Bruker SAINT software package was used to integrate diffraction frames.[4] The diffraction data were corrected for absorption effects using the SADABS program.[5] The structure determination and refinement, using the OLEX2 interface,[6] were performed by using the full matrix least-squares method based on F^2 against all reflections with SHELXL-2014/7.[7] PLATON program used to verify the final CIF for any missed symmetry.[8] PLATON[8] and PARST[9] programs were used for geometrical calculations. OLEX26 and POV-Ray[10] were used to generate graphics containing the structures. Additional details of the data collection and structural refinement parameters are provided in supplementary information tables S1 and S5.

Nanoindentation. Crystals of form I was used for nanoindentation at ambient conditions. The indentation experiments were performed by using Agilent G200 nanoindenter equipped with an XP head and using a Berkovich diamond indenter. The experiments were performed by using the continuous stiffness method to a selected depth with a strain rate of 0.05 s^{-1}, an amplitude of 2 nm, and a frequency of 45 Hz. Prior to nanoindentation experiments of the samples, calibration of the indenter was performed by using Corning 7980 silica reference sample (Nanomechanics S1495-25).[11,12] The modulus was measured between 200 and 500 nm for a total of 16 clean indents. The value of the Poisson's ratio was assumed to be 0.30.

Solid-State NMR Spectroscopy. Solid-state NMR (SSNMR) spectra were acquired at 14.1 T and 18.8 T at the National High Magnetic Field Laboratory (Tallahassee, Florida) using Bruker NEO consoles and Oxford wide-bore magnets, where the relevant Larmor frequencies at 14.1 T are $\nu_0(^1H)$ = 600.1 MHz and $\nu_0(^{13}C)$ = 150.895 MHz and at 18.8 T are $\nu_0(^1H)$ = 799.744 MHz and $\nu_0(^2H)$ = 122.765 MHz. Static NMR experiments were conducted at 18.8 T using a home-built 5 mm HX static probe. Samples were packed into 5 mm polychlorotrifluoroethylene (PCTFE or Kel-F) sample holders with Viton O-rings designed at the NHMFL and machined by Shenzhen Rapid Direct Co., Ltd. MAS NMR experiments were conducted at 14.1 T using a home-built 3.2 mm HXY MAS probe with samples packed into 3.2 mm o.d. zirconia rotors.

$^1H \rightarrow ^{13}C\{^1H\}$ CP/MAS SSNMR. The $^1H \rightarrow ^{13}C\{^1H\}$ ramped CP/MAS pulse sequence[13,14,15,16,17] was used to acquire the ^{13}C SSNMR spectra at 14.1 T, with ^1H π/2 pulses of 2.5 μs, contact times of 1 ms, Hartman Hahn matching fields of $\nu_1(^{13}C)$ = 50 kHz and $\nu_2(^1H)$ = 50 kHz, SPINAL-64 ^1H decoupling (ν_2 = 100 kHz) and a MAS rate of ν_{rot} = 14 kHz. ^{13}C chemical shifts were referenced to TMS at $\delta_{iso}(^{13}C)$ = 0.0 ppm using the carboxyl peak of ^{13}C-labeled a-glycine at $\delta_{iso}(^{13}C)$ = 176.5 ppm as a secondary reference.[18] Variable-temperature (VT) $^1H \rightarrow ^{13}C$ CP/MAS experiments

were conducted over a temperature range from 308 K to 200 K (first by cooling, and then by heating, in order to detect phase transitions and any possible hysteresis effects). The temperature was calibrated using lead nitrate under MAS conditions to obtain a linear relationship between temperature and MAS rate.[19] The temperature was allowed to equilibrate for a minimum of five minutes at each temperature. A summary of the $^{1}H \rightarrow ^{13}C$ CP/MAS experimental parameters is given in **Table S3**.

^{2}H SSNMR Spectroscopy. $^{2}H\{^{1}H\}$ SSNMR spectra were acquired under static conditions at 18.8 T using a quadrupolar Carr-Purcell Meiboom-Gill (QCPMG)[20] pulse sequence of the form $\pi/2 - \tau - \pi/2 - \tau -$ acq, with $\pi/2$ pulse widths of 3 μs ($\nu_1(^{2}H)$ = 83.3 kHz), recycle delays of 90 to 1080 s, and a continuous wave (CW) decoupling field of $\nu_2(^{1}H)$ = 50 kHz. ^{2}H chemical shifts were referenced externally to TMS via the resonance of D_2O(l) at $\delta_{iso}(^{2}H)$ = 4.74 ppm as a secondary reference. VT $^{2}H\{^{1}H\}$ experiments were conducted over a temperature range from 295.5 to 200 K, first by cooling, followed by heating. A summary of the $^{2}H\{^{1}H\}$ experimental parameters is given in Supporting Table S4.

Molecular mechanics (MM) and dynamics (MD). All MM calculations and MD simulations were carried out within the Materials Studio (MS) 8 suite of programs.[21] The Forcite code implemented in MS was used in all cases. We employed the Dreiding force field (FF) for energy evaluations,[22] and the QEq_neutral_1.0 scheme to generate atomic charges with a convergence limit of 2×10^{-5} kcal/mol.[23] Electrostatic and Van der Waals interactions were calculated using atom-based summations, with a cubic spline-based truncation and a cutoff value of 18.5 Å. For H-bond interactions we used a 4.5 Å cutoff.

For both forms I and II of crystalline **1**, supercells of 2×2×1 size were built and optimized in three steps: (1) all H-atoms with heavy atoms and cell parameters fixed, then, (2) heavy atoms were released and, finally, (3) a full unconstrained optimization was performed with based on the FF setup, using the Smart algorithm implemented in Forcite,[21] which is a cascade of the steepest descent, ABNR, and quasi-Newton methods. Convergence thresholds for energies and forces were set to 2×10^{-5} kcal/mol and 1×10^{-3} kcal/(mol·Å), respectively. Hessian matrices were diagonalized upon optimization, with all eigenvalues positive, confirming local minima for both forms I and II.

MM-optimized geometries were used as starting points for molecular dynamics. The MD setup employed the NPT thermodynamic ensemble, where the pressure and temperature were kept at 1 atm and temperatures 300 K and 250 K were employed for forms I and II, respectively, using the Nose thermostat with a Q ratio of 0.01,[24] and the Berendsen barostat with a decay constant of 0.1 ps.[25] The time step was 1 fs in all cases. The MD protocol consisted of a 1000 ps equilibration run, followed by

a 10 ns production run. A second 10 ns production run, (yielding total simulation times of 20 ns) confirmed that average energies converged under this scheme.

Density functional theory (DFT) calculations

DFT calculations of periodic systems were carried out using Dmol3,[26] with the M06-L functional and the TNP all-electron triple-zeta numerical basis.[27,28] The self-consistent field energies reported were obtained using the M06-L/TNP method with a convergence threshold set to 10^{-6} Ha (6.2×10^{-4} kcal/mol), *in vacuo*. Single-molecule calculations were carried out with the Gaussian 16 software,[29] using the hybrid M06-2X functional and the gaussian basis set def2TZVP,[30,31] with an energy convergence threshold set to 10^{-8} Ha (6.2×10^{-6} kcal/mol).

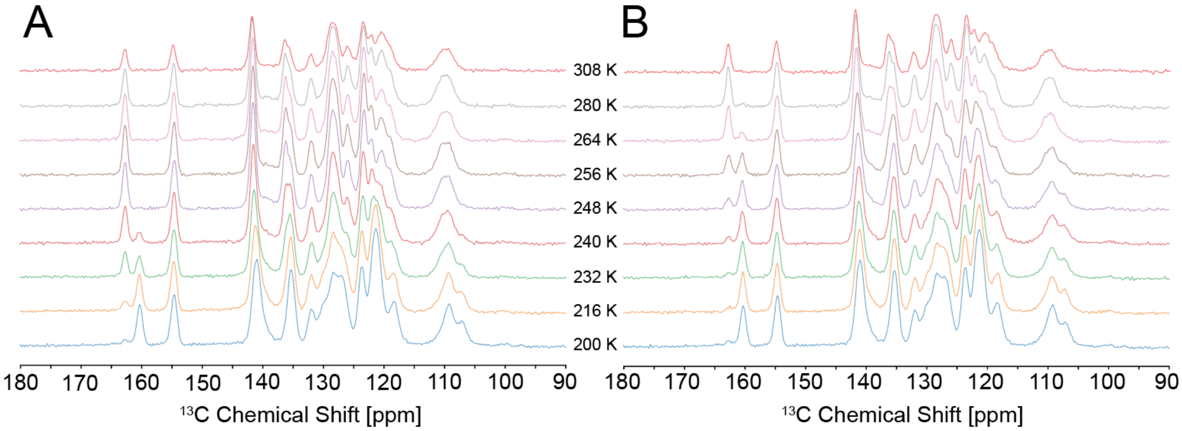

Figure S1. Variable-temperature $^{1}H\rightarrow{}^{13}C\{^{1}H\}$ CP/MAS NMR spectra of compound **1** acquired at 14.1 T and ν_{rot} = 14 kHz as the sample was (A) cooled and then (B) heated.

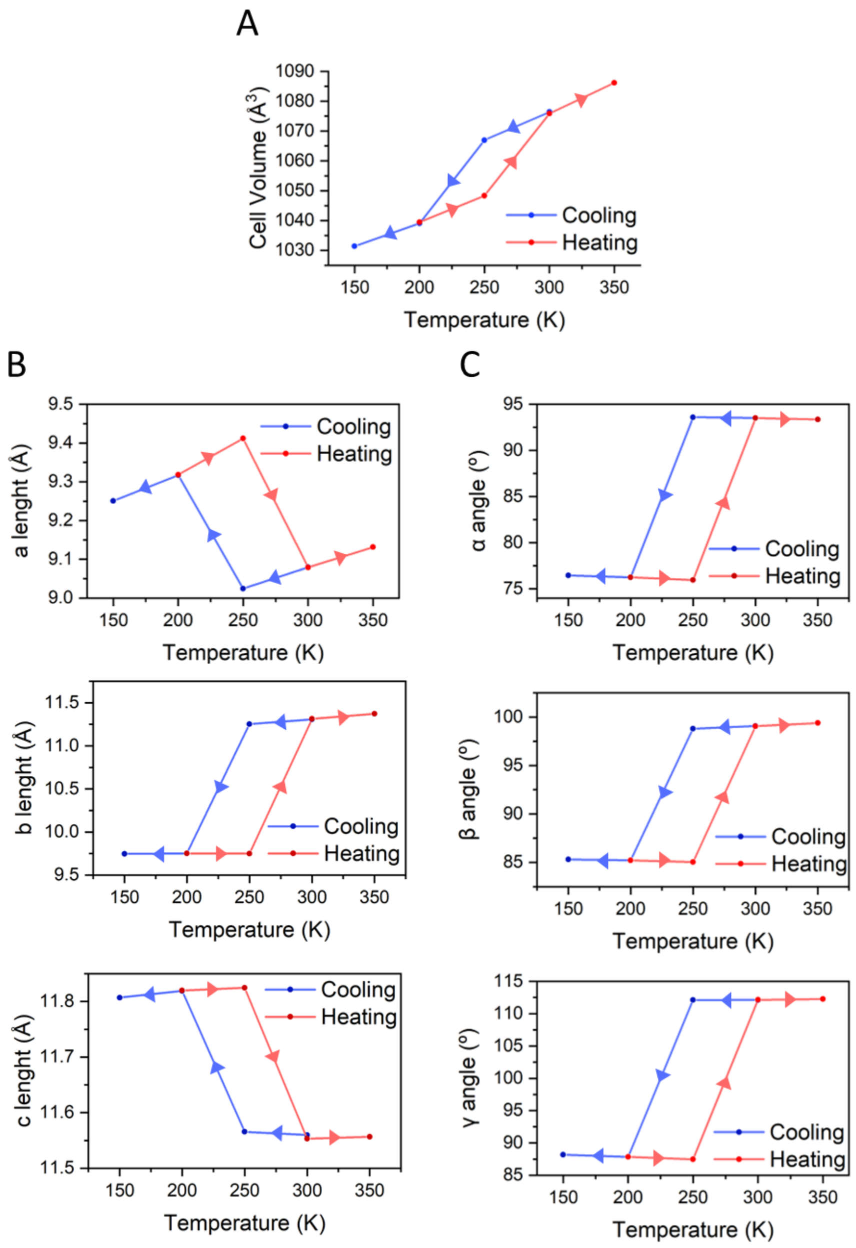

Figure S2. Parameters such as cell volume (A), axis length (B), and angles (C) variation at different temperatures while cooling or heating the sample. Hysteresis is present and associated with the cooling or heating process.

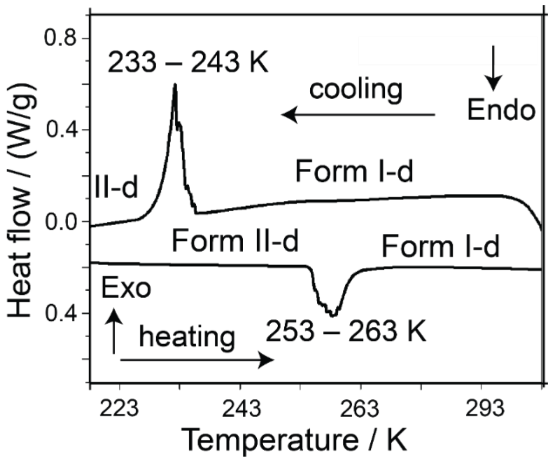

Figure S3. The DSC analysis of the deuterated compound **1-*d*$_4$** shows a similar reversible phase transition to that observed for compound **1** at low temperature.

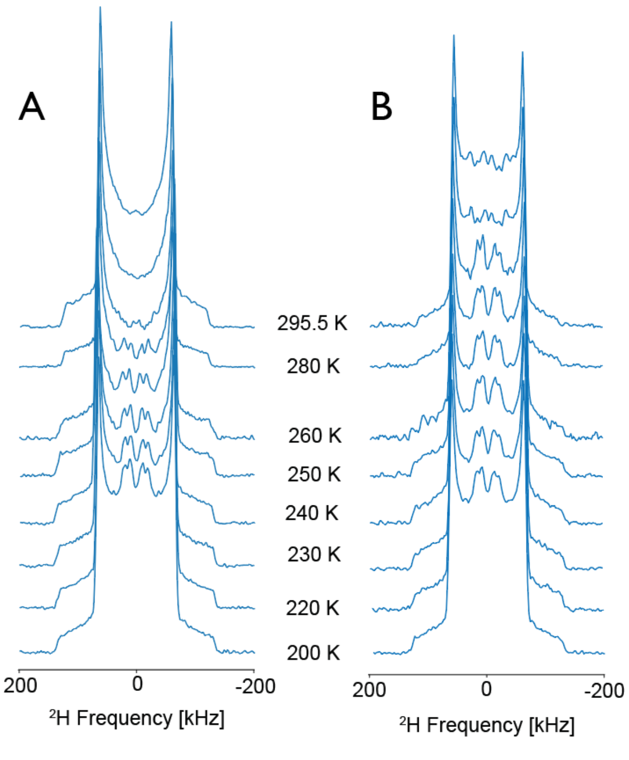

Figure S4. Variable-temperature ^{2}H{^{1}H} static quadrupolar-echo spectra of compound **1** acquired at 18.8 T as the sample was (A) cooled and then (B) heated.

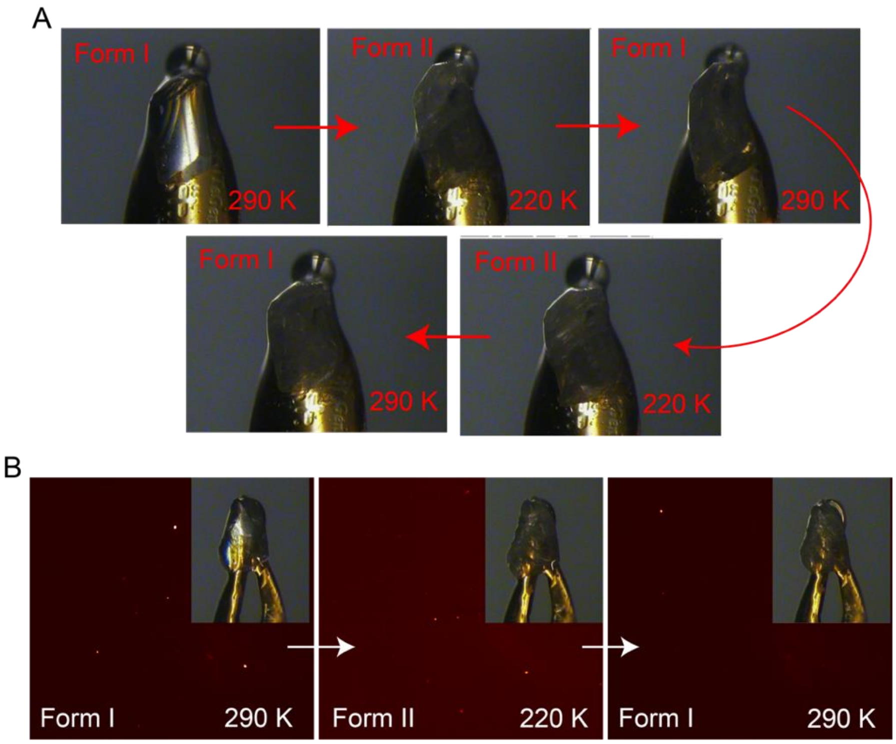

Figure S5. (A) Reversible single-crystal-to-single-crystal transition of form I crystals. A single crystal was used for in situ X-ray diffraction experiments. The form I crystal, mounted on the diffractometer, was first cooled to induce the transition from form I to form II, then heated to reverse the transition from form II back to form I, and subsequently cooled again to repeat the transition from form I to form II. (B) Diffraction quality during phase transition. Note: During the phase transition from form I to form II, the crystal's transparency was slightly reduced and also defects and cracks were observed. These defects became more pronounced with further cooling and heating cycles. After 2 to 3 cycles, the crystal's integrity as a single entity was compromised, eventually breaking into smaller crystalline fragments. Nevertheless, these smaller crystals continued to exhibit jumping behavior.

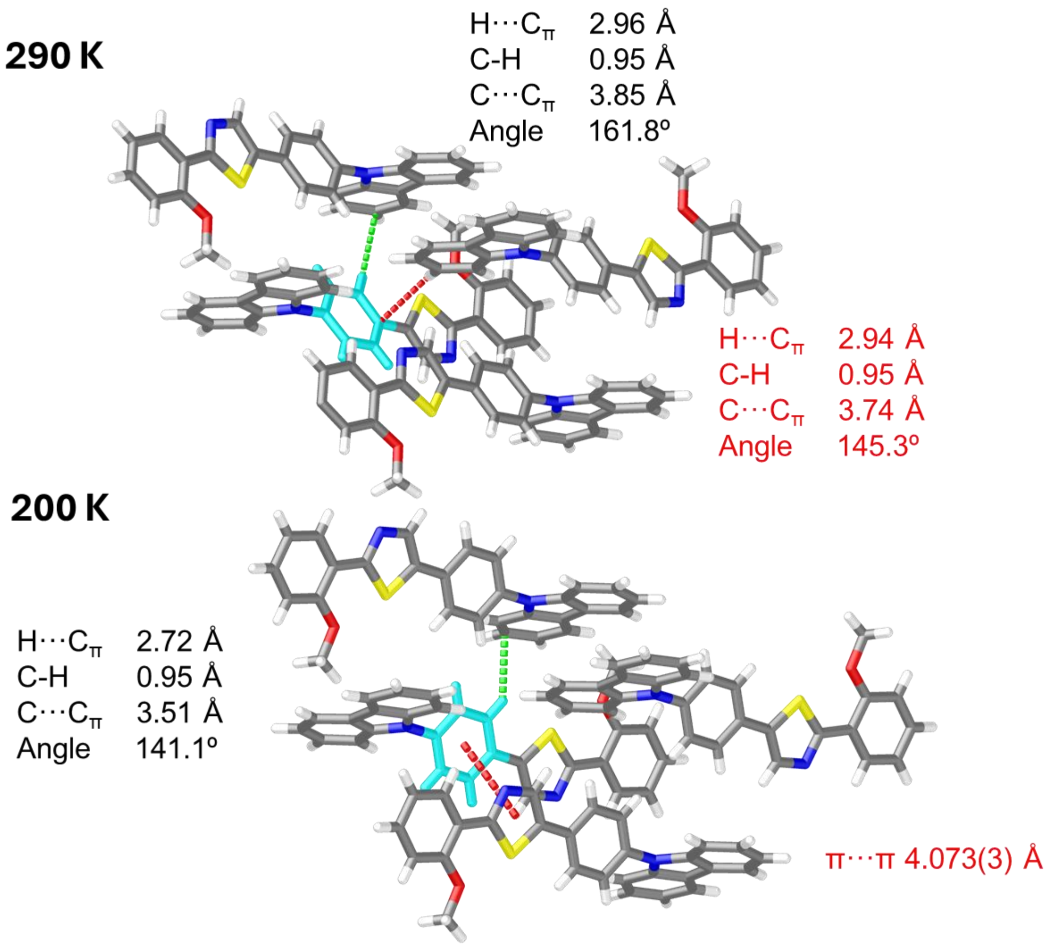

Figure S6. Supramolecular interactions of mobile phenylene (cyan highlighted), at high and low temperature.

Table S1. Relevant crystallographic parameters for **1** at high (Form I, 300 K) and low (Form II, 200 K) temperatures

Radiation/Å	Synchrotron / 0.7288								MoK_{α} 0.71073		
	**Cooling**				**Heating**				**R.T.**	**Cooling**	**Heating**
	**Form I**	**Form I**	**Form II**	**Form II**	**Form II**	**Form II**	**Form I**	**Form I**	**Form I**	**Form II**	**Form II**
**CCDC**	**2406302**	**2406299**	**2406298**	**2406297**	**2406303**	**2406301**	**2406300**	**2406296**	2406385	2406386	2406424
T/K	300 (2)	250	200	150	200(2)	250	300	350	290	200	290
Crystal system	Triclinic										
Space group	$P\bar{1}$										
Z	2										
Z'	1										
a/Å	9.0798(10)	9.0244(8)	9.3167(9)	9.2508(10)	9.3185(9)	9.4117(10)	9.0790(9)	9.1315(10)	9.0482(7)	9.3165(13)	9.0383(18)
b/Å	11.3099(12)	11.2518(10)	9.7504(9)	9.7467(11)	9.7517(9)	9.7494(10)	11.3135(11)	11.3726(12)	11.2757(9)	9.7354(12)	11.265(2)
c/Å	11.5596(12)	11.5655(11)	11.8192(11)	11.8070(13)	11.8199(11)	11.8240(12)	11.5529(11)	11.5567(12)	11.5271(9)	11.7977(15)	11.502(2)
α/°	93.518(4)	93.600(3)	76.242(3)	76.458(4)	76.244(3)	75.955(4)	93.509(4)	93.363(4)	93.629(3)	76.334(4)	93.570(6)
β/°	99.069(4)	98.790(4)	85.217(3)	85.318(4)	85.207(3)	85.046(4)	99.049(4)	99.391(4)	99.053(2)	85.051(4)	99.044(6)
γ/°	112.133(4)	111.983(3)	87.851(4)	88.153(4)	87.849(3)	87.451(4)	112.155(3)	112.270(4)	112.116(2)	87.674(4)	112.215(6)
V/Å^3	1076.4(2)	1066.93(17)	1039.07(17)	1031.4(2)	1039.46(17)	1048.28(19)	1075.90(18)	1086.2(2)	1066.25(15)	1035.7(2)	1061.2(4)
μ/mm^{-1}	0.183	0.185	0.190	0.191	0.190	0.188	0.183	0.182	0.176	0.181	0.177
Reflections Collected	35754	35405	34229	33524	38809	39516	33957	29456	11795	13903	16204
Independent Reflections	6583 [*R*(int) = 0.0374]	6535 [*R*(int) = 0.0359]	6364 [*R*(int) = 0.0366]	6311 [*R*(int) = 0.0384]	7946 [*R*(int) = 0.0365]	8011 [*R*(int) = 0.0375]	6043 [*R*(int) = 0.0377]	4808 [*R*(int) = 0.0382]	3689 [R_{int} = 0.0273, R_{sigma} = 0.0297]	3588 [R_{int} = 0.0545, R_{sigma} = 0.0463]	3595 [R_{int} = 0.0658, R_{sigma} = 0.0526]
Goodness of fit on F^2	1.043	1.051	1.037	1.045	1.024	1.057	1.049	1.066	1.058	1.035	1.080
Final *R* indices [I>2σ(I)]	R_1 = 0.0512, wR_2 = 0.1457	R_1 = 0.0495, wR_2 = 0.1385	R_1 = 0.0368, wR_2 = 0.0965	R_1 = 0.0347, wR_2 = 0.0937	R_1 = 0.0380, wR_2 = 0.1034	R_1 = 0.0403, wR_2 = 0.1110	R_1 = 0.0508, wR_2 = 0.1467	R_1 = 0.0471, wR_2 = 0.1298	R_1= 0.0480, wR_2 = 0.1132	R_1= 0.0484, wR_2 = 0.1104	R_1 = 0.0791, wR_2 = 0.1534
R índices (all data)	R_1 = 0.0601, wR_2 = 0.1553	R_1 = 0.0558, wR_2 = 0.1448	R_1 = 0.0411, wR_2 = 0.1007	R_1 = 0.0391, wR_2 = 0.0975	R_1 = 0.0446, wR_2 = 0.1091	R_1 = 0.0505, wR_2 = 0.1193	R_1 = 0.0647, wR_2 = 0.1606	R_1 = 0.0579, wR_2 = 0.1394	R_1 = 0.0614, wR_2 = 0.1188	R_1 = 0.0649, wR_2 = 0.1184	R_1 = 0.1098, wR_2= 0.1640

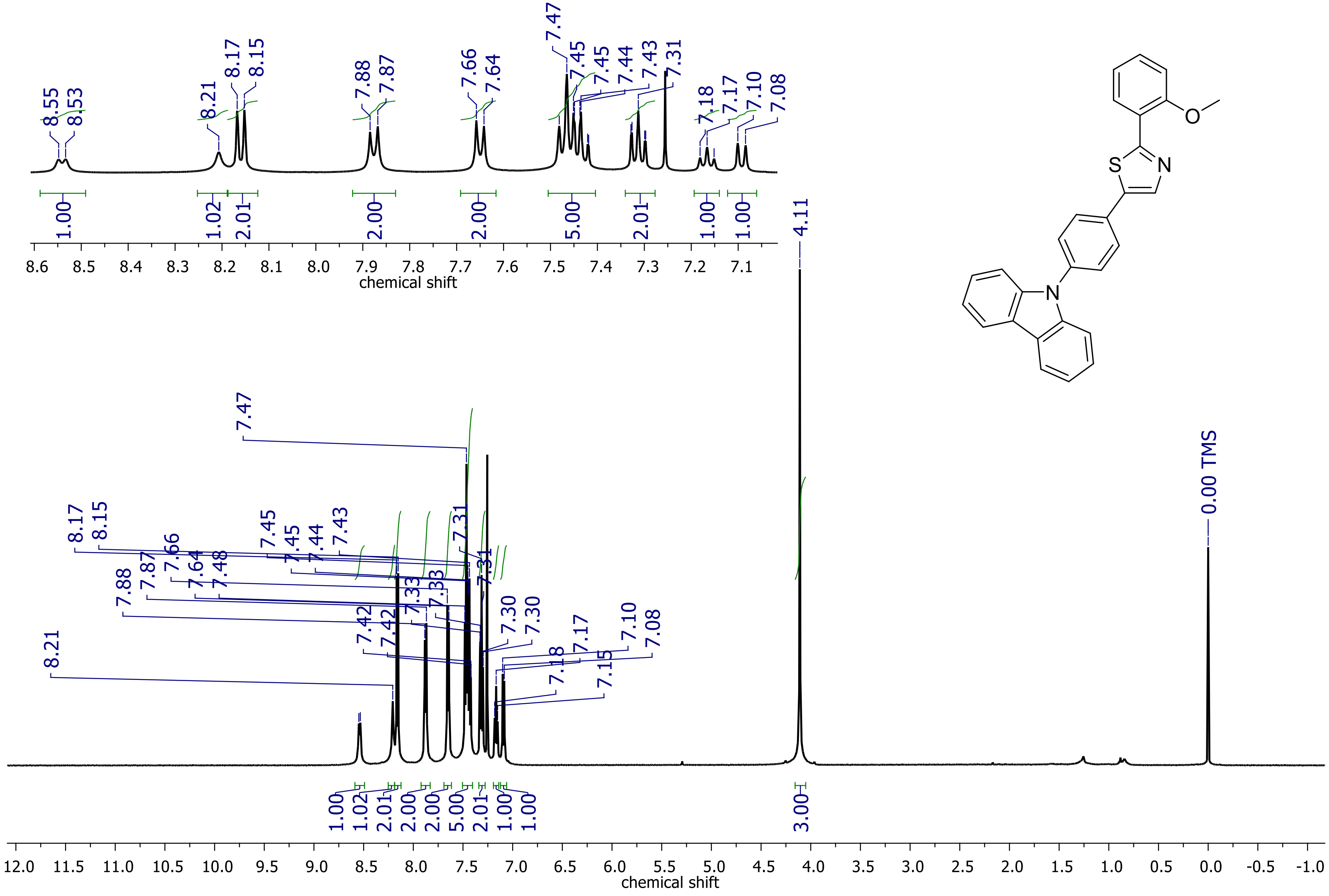

Figure S7. ^{1}H NMR of compound **1** in $CDCl_3$ at 500 MHz.

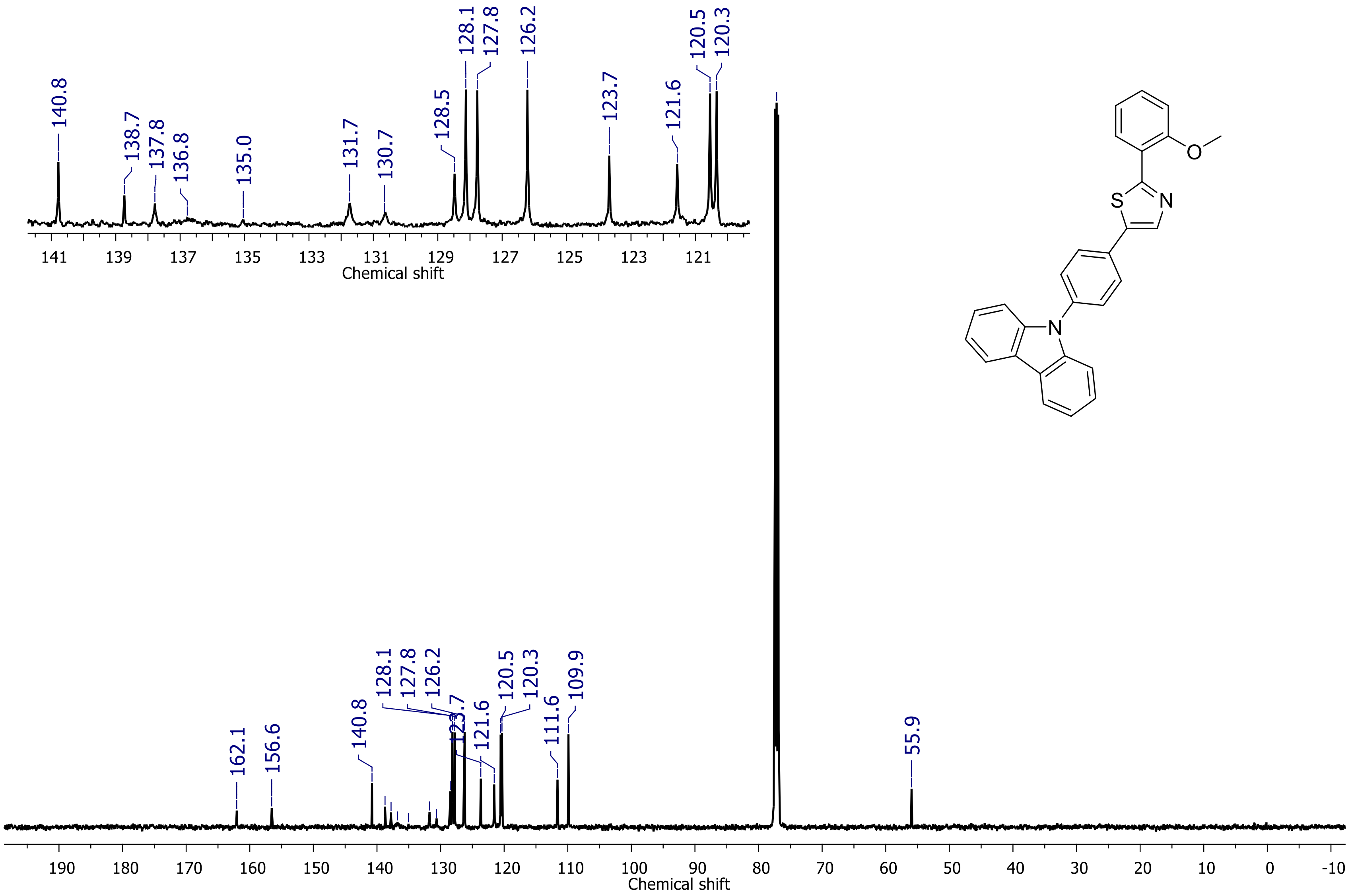

Figure S8. ^{13}C NMR of compound **1** in $CDCl_3$ at 126 MHz.

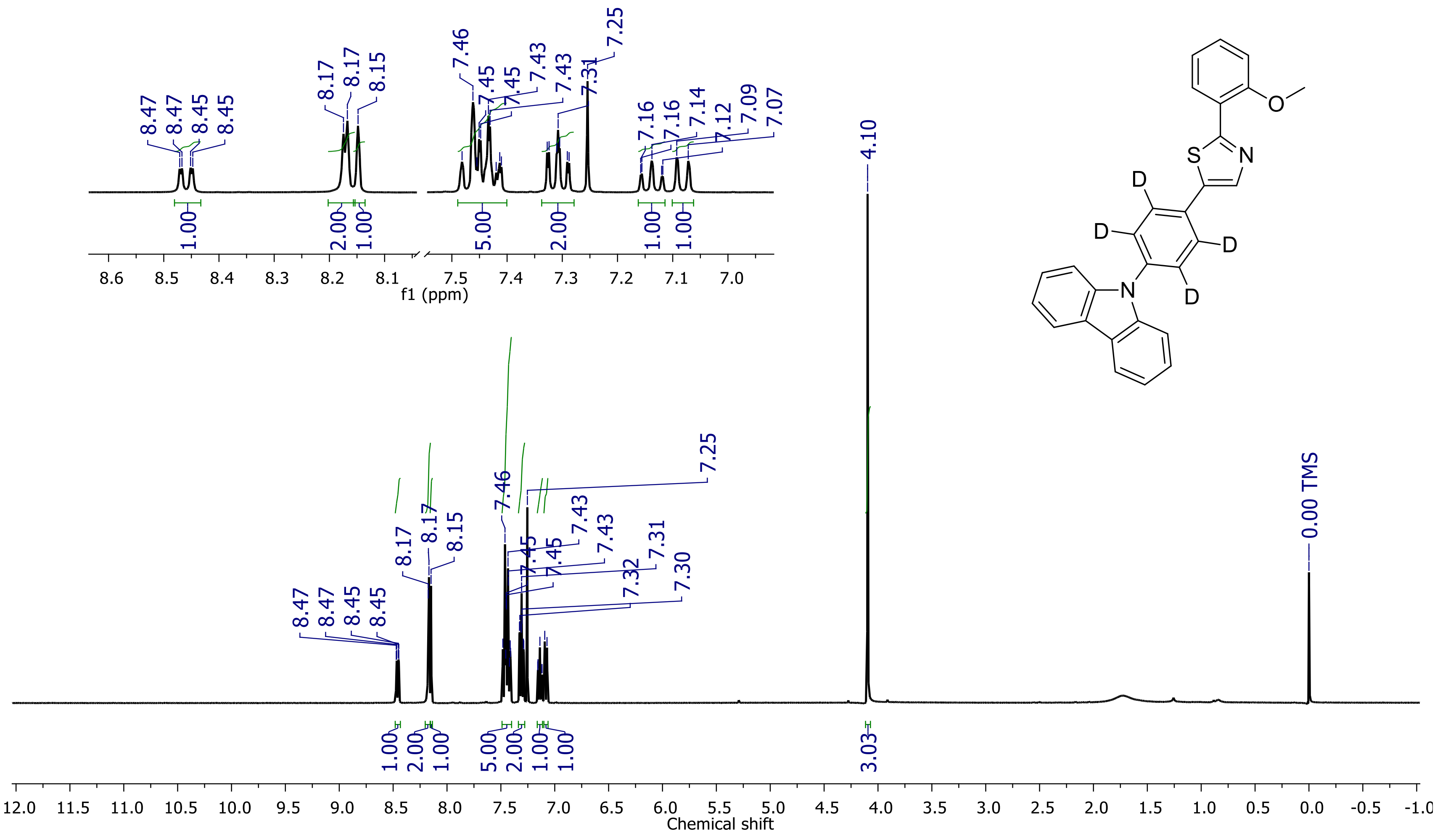

Figure S9. ^{1}H NMR of compound **1-*d*$_4$** in $CDCl_3$ at 500 MHz.

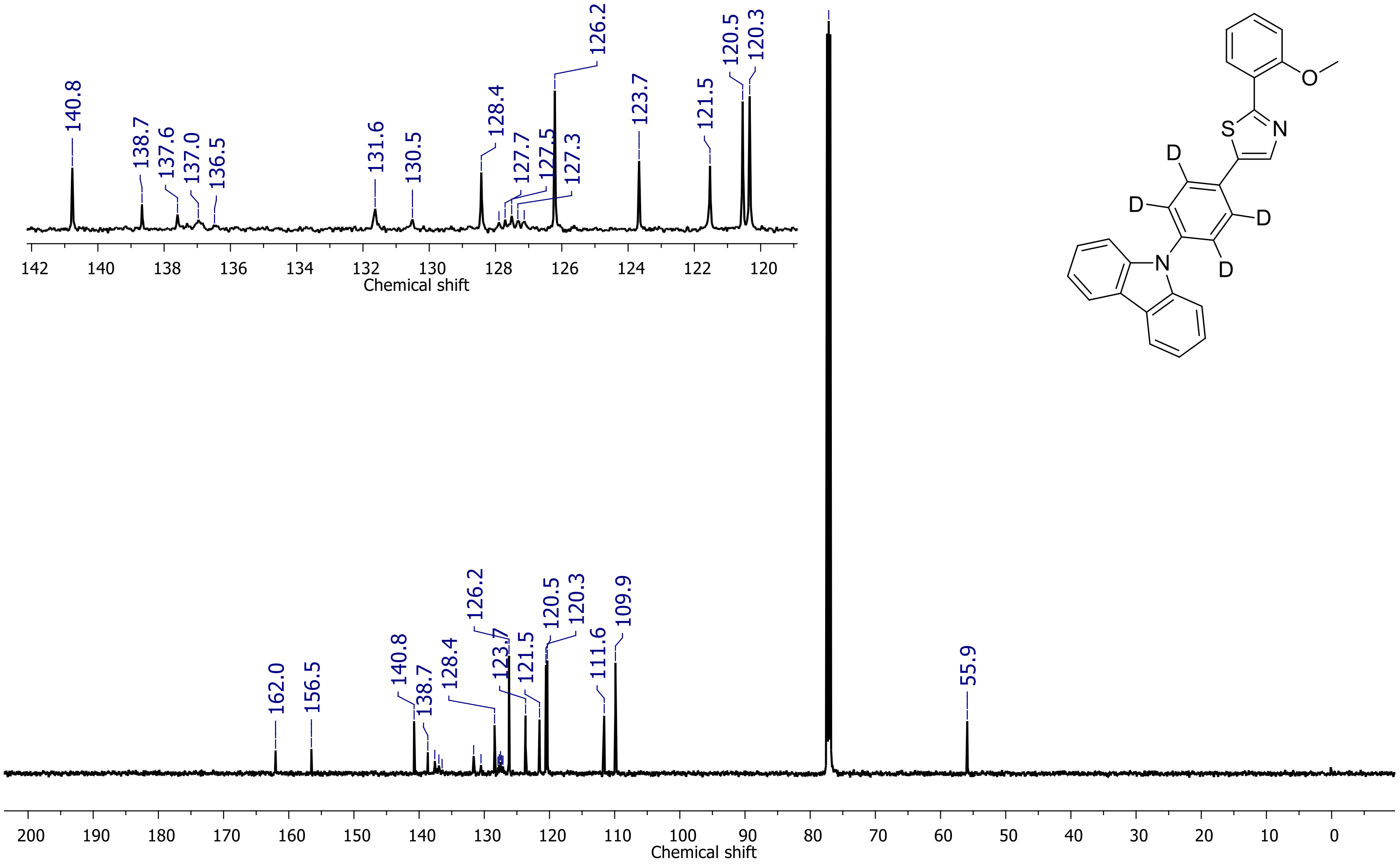

Figure S10. ^{13}C NMR of compound **1-*d*$_4$** in $CDCl_3$ at 126 MHz.

Table S2. Harmonic vibrational frequencies, and their corresponding vibrational entropy values (S_{vib}), for forms I and II of crystalline compound **1**. Frequencies were calculated by the diagonalization of the hessian matrix of potential energy second derivatives vs atomic displacements. Potential energies were calculated classically, employing the Dreiding forcefield, with the QEq equilibration scheme for neutral compounds.

HIGH TEMPERATURE FORM			LOW TEMPERATURE FORM		
Wavenumber	Frequency	S_{vib}	Wavenumber	Frequency	S_{vib}
cm^{-1}	$x10^{12}$ s^{-1}	J/K	cm^{-1}	$x10^{12}$ s^{-1}	J/K
35.5	1.06	2.77	33.5	1.00	2.65
36.4	1.09	2.75	34.6	1.04	2.62
36.4	1.09	2.75	36.0	1.08	2.57
40.0	1.20	2.65	37.4	1.12	2.54
41.0	1.23	2.63	37.7	1.13	2.53
41.4	1.24	2.62	37.8	1.13	2.53
41.5	1.25	2.61	38.4	1.15	2.51
43.3	1.30	2.57	40.2	1.21	2.47
44.1	1.32	2.56	40.5	1.22	2.46
45.3	1.36	2.53	41.2	1.23	2.44
48.1	1.44	2.47	43.0	1.29	2.40
48.9	1.47	2.45	45.9	1.38	2.33
49.1	1.47	2.45	46.0	1.38	2.33
50.6	1.52	2.42	46.6	1.40	2.32
50.7	1.52	2.42	47.0	1.41	2.31
53.4	1.60	2.36	47.9	1.44	2.29
53.9	1.62	2.36	47.9	1.44	2.29
54.8	1.64	2.34	48.3	1.45	2.28
55.5	1.67	2.33	51.7	1.55	2.22
56.3	1.69	2.31	53.6	1.61	2.18
56.7	1.70	2.30	54.7	1.64	2.16
57.6	1.73	2.29	59.0	1.77	2.08
58.5	1.76	2.27	59.4	1.78	2.08
58.8	1.76	2.27	61.4	1.84	2.05
59.0	1.77	2.27	63.0	1.89	2.02
59.1	1.77	2.26	63.6	1.91	2.01
59.9	1.80	2.25	64.7	1.94	1.99
60.1	1.80	2.25	64.7	1.94	1.99
61.7	1.85	2.22	67.2	2.02	1.95
65.2	1.96	2.17	67.3	2.02	1.95
65.6	1.97	2.16	67.5	2.03	1.95
66.1	1.98	2.15	68.5	2.06	1.94
67.7	2.03	2.13	69.7	2.09	1.92
68.8	2.06	2.11	71.1	2.13	1.90
69.5	2.09	2.10	71.4	2.14	1.90
69.9	2.10	2.10	73.6	2.21	1.87

71.4	2.14	2.08	75.2	2.26	1.84
73.8	2.21	2.04	77.1	2.31	1.82
74.9	2.25	2.03	78.3	2.35	1.80
75.5	2.26	2.02	78.5	2.35	1.80
75.6	2.27	2.02	78.6	2.36	1.80
76.0	2.28	2.01	80.1	2.40	1.78
77.2	2.32	2.00	80.5	2.41	1.78
78.3	2.35	1.99	81.2	2.44	1.77
79.0	2.37	1.98	81.4	2.44	1.77
79.4	2.38	1.97	83.0	2.49	1.75
80.0	2.40	1.96	85.1	2.55	1.72
80.8	2.42	1.95	85.6	2.57	1.72
81.6	2.45	1.94	86.1	2.58	1.71
83.8	2.51	1.92	86.9	2.61	1.70
84.1	2.52	1.91	88.2	2.65	1.69
84.2	2.53	1.91	90.8	2.72	1.66
86.0	2.58	1.89	94.7	2.84	1.62
86.7	2.60	1.88	97.1	2.91	1.59
87.0	2.61	1.88	97.4	2.92	1.59
89.1	2.67	1.86	98.1	2.94	1.58
89.3	2.68	1.85	98.7	2.96	1.58
90.3	2.71	1.84	100.5	3.01	1.56
91.8	2.75	1.83	102.2	3.07	1.54
94.5	2.84	1.80	103.3	3.10	1.53
94.6	2.84	1.80	105.7	3.17	1.51
94.8	2.84	1.80	107.3	3.22	1.50
96.3	2.89	1.78	107.8	3.23	1.49
97.2	2.92	1.77	108.6	3.26	1.49
97.9	2.94	1.76	108.8	3.26	1.48
102.5	3.07	1.72	108.9	3.27	1.48
103.4	3.10	1.71	110.2	3.31	1.47
105.5	3.17	1.69	111.9	3.36	1.46
106.6	3.20	1.68	112.6	3.38	1.45
109.8	3.29	1.65	113.4	3.40	1.44
111.1	3.33	1.64	113.5	3.40	1.44
112.4	3.37	1.63	113.7	3.41	1.44
113.6	3.41	1.62	114.5	3.43	1.43
113.8	3.41	1.62	115.2	3.46	1.43
117.1	3.51	1.59	115.5	3.47	1.43
117.9	3.54	1.58	119.3	3.58	1.39
118.1	3.54	1.58	121.0	3.63	1.38
118.4	3.55	1.58	121.5	3.65	1.38
119.0	3.57	1.57	121.8	3.65	1.38
120.1	3.60	1.56	124.4	3.73	1.35
122.0	3.66	1.55	124.7	3.74	1.35
122.4	3.67	1.55	125.0	3.75	1.35

122.7	3.68	1.54	125.3	3.76	1.35
123.1	3.69	1.54	125.6	3.77	1.35
124.5	3.73	1.53	126.2	3.79	1.34
124.9	3.75	1.53	127.0	3.81	1.33
128.4	3.85	1.50	127.8	3.83	1.33
131.1	3.93	1.48	131.0	3.93	1.31
132.1	3.96	1.47	132.2	3.97	1.30
132.9	3.99	1.47	132.4	3.97	1.30
133.0	3.99	1.47	132.6	3.98	1.29
134.9	4.05	1.45	133.9	4.02	1.28
137.5	4.12	1.43	134.1	4.02	1.28
138.4	4.15	1.43	134.6	4.04	1.28
140.6	4.22	1.41	135.0	4.05	1.28
141.9	4.26	1.40	135.5	4.06	1.27
143.4	4.30	1.39	137.1	4.11	1.26
145.6	4.37	1.38	141.7	4.25	1.23
146.5	4.40	1.37	142.3	4.27	1.23
149.2	4.48	1.35	144.1	4.32	1.22
149.4	4.48	1.35	144.7	4.34	1.21
150.3	4.51	1.35	147.7	4.43	1.19
150.6	4.52	1.35	152.0	4.56	1.16
152.2	4.57	1.34	152.1	4.56	1.16
153.5	4.60	1.33	154.2	4.63	1.15
153.7	4.61	1.33	156.0	4.68	1.14
155.7	4.67	1.31	157.3	4.72	1.13
156.7	4.70	1.31	159.1	4.77	1.12
156.8	4.70	1.31	159.3	4.78	1.12
159.4	4.78	1.29	160.4	4.81	1.11
160.9	4.83	1.28	160.6	4.82	1.11
162.5	4.88	1.27	160.6	4.82	1.11
162.8	4.88	1.27	163.1	4.89	1.10
163.6	4.91	1.27	164.7	4.94	1.09
163.8	4.91	1.27	165.5	4.97	1.08
164.3	4.93	1.26	166.5	4.99	1.08
169.5	5.09	1.23	168.0	5.04	1.07
174.7	5.24	1.21	176.7	5.30	1.02
176.3	5.29	1.20	178.3	5.35	1.02
177.8	5.34	1.19	179.5	5.38	1.01
178.0	5.34	1.19	185.8	5.57	0.98
178.7	5.36	1.18	186.9	5.61	0.97
181.1	5.43	1.17	189.1	5.67	0.96
184.4	5.53	1.15	189.4	5.68	0.96
184.5	5.54	1.15	191.7	5.75	0.95
184.7	5.54	1.15	194.6	5.84	0.94
186.7	5.60	1.14	194.7	5.84	0.94
187.2	5.62	1.14	200.4	6.01	0.91

189.5	5.68	1.13	201.3	6.04	0.91
191.7	5.75	1.12	201.5	6.04	0.91
192.0	5.76	1.12	206.5	6.20	0.88
192.7	5.78	1.11	210.4	6.31	0.87
196.7	5.90	1.09	213.0	6.39	0.86
199.9	6.00	1.08	213.2	6.40	0.86
203.6	6.11	1.06	213.7	6.41	0.85
203.7	6.11	1.06	215.4	6.46	0.85
205.0	6.15	1.06	215.5	6.47	0.85
206.6	6.20	1.05	215.9	6.48	0.84
206.7	6.20	1.05	217.0	6.51	0.84
206.9	6.21	1.05	220.4	6.61	0.83
207.4	6.22	1.04	221.7	6.65	0.82
209.1	6.27	1.04	222.3	6.67	0.82
211.2	6.33	1.03	223.1	6.69	0.82
211.3	6.34	1.03	224.6	6.74	0.81
211.4	6.34	1.03	224.8	6.74	0.81
211.9	6.36	1.03	228.4	6.85	0.79
213.8	6.41	1.02	230.0	6.90	0.79
217.9	6.54	1.00	230.6	6.92	0.79
220.3	6.61	0.99	231.9	6.96	0.78
227.9	6.84	0.96	235.4	7.06	0.77
229.7	6.89	0.95	236.9	7.11	0.76
241.3	7.24	0.91	238.6	7.16	0.76
242.3	7.27	0.90	240.4	7.21	0.75
244.0	7.32	0.90	242.4	7.27	0.74
245.9	7.38	0.89	242.7	7.28	0.74
253.6	7.61	0.86	243.4	7.30	0.74
253.8	7.61	0.86	244.6	7.34	0.74
254.3	7.63	0.86	261.3	7.84	0.68
255.1	7.65	0.86	261.8	7.85	0.68
256.3	7.69	0.85	262.0	7.86	0.68
258.2	7.75	0.85	262.1	7.86	0.68
259.2	7.78	0.84	262.3	7.87	0.68
260.1	7.80	0.84	262.6	7.88	0.68
260.7	7.82	0.84	262.9	7.89	0.68
260.7	7.82	0.84	263.0	7.89	0.68
261.3	7.84	0.84	267.5	8.02	0.66
262.4	7.87	0.83	269.7	8.09	0.65
263.8	7.91	0.83	270.0	8.10	0.65
264.3	7.93	0.83	270.2	8.11	0.65
264.8	7.94	0.83	271.6	8.15	0.65
265.2	7.96	0.82	272.8	8.18	0.65
265.9	7.98	0.82	273.1	8.19	0.64
269.6	8.09	0.81	275.4	8.26	0.64
294.1	8.82	0.73	294.4	8.83	0.58

294.2	8.83	0.73	294.8	8.84	0.58
299.4	8.98	0.72	301.1	9.03	0.57
301.1	9.03	0.71	304.7	9.14	0.56
305.8	9.17	0.70	313.4	9.40	0.54
307.1	9.21	0.70	315.1	9.45	0.53
313.0	9.39	0.68	315.9	9.48	0.53
313.8	9.42	0.68	320.8	9.63	0.52
323.1	9.69	0.66	324.6	9.74	0.51
324.8	9.74	0.65	328.1	9.84	0.50
325.2	9.75	0.65	328.5	9.86	0.50
326.0	9.78	0.65	328.5	9.86	0.50
326.3	9.79	0.65	328.6	9.86	0.50
326.3	9.79	0.65	329.7	9.89	0.50
327.9	9.84	0.64	329.7	9.89	0.50
329.0	9.87	0.64	329.8	9.90	0.50
329.7	9.89	0.64	330.1	9.90	0.50
330.3	9.91	0.64	331.1	9.93	0.49
330.3	9.91	0.64	333.6	10.01	0.49
330.7	9.92	0.64	335.2	10.05	0.48
330.8	9.92	0.64	339.2	10.18	0.48
331.5	9.94	0.63	340.2	10.21	0.47
332.5	9.97	0.63	340.3	10.21	0.47
332.9	9.99	0.63	341.7	10.25	0.47
334.3	10.03	0.63	341.9	10.26	0.47
337.2	10.12	0.62	343.3	10.30	0.47
337.8	10.13	0.62	343.7	10.31	0.47
338.2	10.15	0.62	346.4	10.39	0.46
338.5	10.15	0.62	347.8	10.43	0.46
339.6	10.19	0.62	349.6	10.49	0.45
340.8	10.22	0.61	350.8	10.52	0.45
342.1	10.26	0.61	351.9	10.56	0.45
345.3	10.36	0.60	354.1	10.62	0.44
347.7	10.43	0.60	354.9	10.65	0.44
348.4	10.45	0.59	355.1	10.65	0.44
349.0	10.47	0.59	356.7	10.70	0.44
349.4	10.48	0.59	358.4	10.75	0.44
349.5	10.49	0.59	359.9	10.80	0.43
352.1	10.56	0.59	360.4	10.81	0.43
354.6	10.64	0.58	362.0	10.86	0.43
373.4	11.20	0.54	384.7	11.54	0.39
373.4	11.20	0.54	385.7	11.57	0.39
373.7	11.21	0.54	386.1	11.58	0.38
374.9	11.25	0.54	386.4	11.59	0.38
375.9	11.28	0.54	386.9	11.61	0.38
376.8	11.30	0.53	387.7	11.63	0.38
377.3	11.32	0.53	387.8	11.63	0.38

378.6	11.36	0.53	389.0	11.67	0.38
410.3	12.31	0.47	389.2	11.68	0.38
410.4	12.31	0.47	389.7	11.69	0.38
410.9	12.33	0.47	391.6	11.75	0.37
411.7	12.35	0.47	393.7	11.81	0.37
411.9	12.36	0.47	402.4	12.07	0.36
412.0	12.36	0.47	405.1	12.15	0.35
413.9	12.42	0.46	405.1	12.15	0.35
414.0	12.42	0.46	407.6	12.23	0.35
420.0	12.60	0.45	407.8	12.23	0.35
421.2	12.64	0.45	408.9	12.27	0.35
421.6	12.65	0.45	414.1	12.42	0.34
422.0	12.66	0.45	414.1	12.42	0.34
422.6	12.68	0.45	417.3	12.52	0.33
423.2	12.70	0.45	421.9	12.66	0.33
423.9	12.72	0.45	422.4	12.67	0.33
424.8	12.74	0.44	424.2	12.73	0.32
425.0	12.75	0.44	428.3	12.85	0.32
425.6	12.77	0.44	428.4	12.85	0.32
425.8	12.77	0.44	428.9	12.87	0.32
426.2	12.78	0.44	429.4	12.88	0.32
427.4	12.82	0.44	430.4	12.91	0.31
428.1	12.84	0.44	431.8	12.95	0.31
430.0	12.90	0.44	432.1	12.96	0.31
430.7	12.92	0.43	435.8	13.07	0.31
433.0	12.99	0.43	435.8	13.07	0.31
433.7	13.01	0.43	436.0	13.08	0.31
434.6	13.04	0.43	438.7	13.16	0.30
434.7	13.04	0.43	438.9	13.17	0.30
435.0	13.05	0.43	439.1	13.17	0.30
435.1	13.05	0.43	439.1	13.17	0.30
435.3	13.06	0.43	439.4	13.18	0.30
436.9	13.11	0.42	439.4	13.18	0.30
453.0	13.59	0.40	450.5	13.51	0.29
453.2	13.60	0.40	450.8	13.52	0.29
453.3	13.60	0.40	451.3	13.54	0.29
453.4	13.60	0.40	451.8	13.55	0.29
454.0	13.62	0.40	453.6	13.61	0.28
454.1	13.62	0.40	454.4	13.63	0.28
454.7	13.64	0.40	454.7	13.64	0.28
454.7	13.64	0.40	455.0	13.65	0.28
473.2	14.20	0.37	472.4	14.17	0.26
473.3	14.20	0.37	472.4	14.17	0.26
473.3	14.20	0.37	472.5	14.18	0.26
473.3	14.20	0.37	472.6	14.18	0.26
473.7	14.21	0.37	472.7	14.18	0.26

473.8	14.22	0.37	472.7	14.18	0.26
474.1	14.22	0.37	472.9	14.19	0.26
474.4	14.23	0.37	473.0	14.19	0.26
481.4	14.44	0.36	488.8	14.67	0.24
481.6	14.45	0.36	489.1	14.67	0.24
481.6	14.45	0.36	489.5	14.68	0.24
482.0	14.46	0.36	490.0	14.70	0.24
482.2	14.47	0.36	490.1	14.70	0.24
482.6	14.48	0.36	490.8	14.72	0.24
482.7	14.48	0.36	490.9	14.73	0.24
483.2	14.49	0.36	491.5	14.74	0.24
486.9	14.61	0.35	498.4	14.95	0.23
487.1	14.61	0.35	499.2	14.98	0.23
487.5	14.63	0.35	499.7	14.99	0.23
488.4	14.65	0.35	499.9	15.00	0.23
488.4	14.65	0.35	499.9	15.00	0.23
488.9	14.67	0.35	500.0	15.00	0.23
490.4	14.71	0.35	500.5	15.01	0.23
494.3	14.83	0.34	500.5	15.01	0.23
512.7	15.38	0.32	513.3	15.40	0.22
513.3	15.40	0.32	513.7	15.41	0.22
513.6	15.41	0.32	513.9	15.42	0.22
514.0	15.42	0.32	514.2	15.43	0.21
514.1	15.42	0.32	514.4	15.43	0.21
514.2	15.43	0.32	514.4	15.43	0.21
514.7	15.44	0.32	514.8	15.44	0.21
515.2	15.46	0.32	515.0	15.45	0.21
528.1	15.84	0.30	515.9	15.48	0.21
528.6	15.86	0.30	516.1	15.48	0.21
528.8	15.86	0.30	517.1	15.51	0.21
528.8	15.86	0.30	517.1	15.51	0.21
528.9	15.87	0.30	517.7	15.53	0.21
529.7	15.89	0.30	518.8	15.56	0.21
531.2	15.93	0.30	520.4	15.61	0.21
531.4	15.94	0.30	520.6	15.62	0.21
535.8	16.07	0.29	540.6	16.22	0.19
536.4	16.09	0.29	541.0	16.23	0.19
536.5	16.10	0.29	542.2	16.27	0.19
536.6	16.10	0.29	542.7	16.28	0.19
537.9	16.14	0.29	545.6	16.37	0.19
538.4	16.15	0.29	546.0	16.38	0.19
538.5	16.15	0.29	548.2	16.45	0.18
540.5	16.21	0.29	549.1	16.47	0.18
563.0	16.89	0.26	567.1	17.01	0.17
563.1	16.89	0.26	567.2	17.01	0.17
564.0	16.92	0.26	567.3	17.02	0.17

564.1	16.92	0.26	567.4	17.02	0.17
564.2	16.92	0.26	567.5	17.02	0.17
564.2	16.93	0.26	567.6	17.03	0.17
564.7	16.94	0.26	568.1	17.04	0.17
564.7	16.94	0.26	569.0	17.07	0.17
575.8	17.27	0.25	576.4	17.29	0.16
577.3	17.32	0.25	577.3	17.32	0.16
577.5	17.32	0.25	577.7	17.33	0.16
577.9	17.34	0.25	578.5	17.35	0.16
578.5	17.36	0.25	578.5	17.35	0.16
579.5	17.39	0.25	578.7	17.36	0.16
581.1	17.43	0.25	578.9	17.37	0.16
581.6	17.45	0.25	579.3	17.38	0.16
585.9	17.58	0.24	579.7	17.39	0.16
586.1	17.58	0.24	580.9	17.43	0.16
586.3	17.59	0.24	581.1	17.43	0.16
586.6	17.60	0.24	581.4	17.44	0.16
587.3	17.62	0.24	585.7	17.57	0.15
587.3	17.62	0.24	588.8	17.66	0.15
587.5	17.62	0.24	589.0	17.67	0.15
587.7	17.63	0.24	589.1	17.67	0.15
596.8	17.90	0.23	590.0	17.70	0.15
597.0	17.91	0.23	592.0	17.76	0.15
597.2	17.92	0.23	592.0	17.76	0.15
598.1	17.94	0.23	593.4	17.80	0.15
600.8	18.02	0.23	594.8	17.84	0.15
601.2	18.04	0.23	595.2	17.86	0.15
601.3	18.04	0.23	597.0	17.91	0.15
601.9	18.06	0.23	597.1	17.91	0.15
610.8	18.32	0.22	613.0	18.39	0.14
611.3	18.34	0.22	613.4	18.40	0.14
611.7	18.35	0.22	613.6	18.41	0.14
612.0	18.36	0.22	613.7	18.41	0.14
612.2	18.36	0.22	614.1	18.42	0.14
612.4	18.37	0.22	614.1	18.42	0.14
612.5	18.38	0.22	614.3	18.43	0.14
613.2	18.40	0.22	614.3	18.43	0.14
645.1	19.35	0.19	647.6	19.43	0.12
645.7	19.37	0.19	648.0	19.44	0.12
646.1	19.38	0.19	648.1	19.44	0.12
646.6	19.40	0.19	648.2	19.45	0.12
646.9	19.41	0.19	648.3	19.45	0.12
647.2	19.42	0.19	648.5	19.45	0.12
647.6	19.43	0.19	649.1	19.47	0.12
647.7	19.43	0.19	650.1	19.50	0.11
648.0	19.44	0.19	659.1	19.77	0.11

649.0	19.47	0.19	659.4	19.78	0.11
649.1	19.47	0.19	659.7	19.79	0.11
650.2	19.50	0.19	659.8	19.79	0.11
652.3	19.57	0.19	661.2	19.84	0.11
652.5	19.57	0.19	661.4	19.84	0.11
653.0	19.59	0.19	661.5	19.85	0.11
653.8	19.61	0.19	661.7	19.85	0.11
670.5	20.11	0.18	672.4	20.17	0.10
670.7	20.12	0.17	672.5	20.18	0.10
670.8	20.12	0.17	672.8	20.18	0.10
670.8	20.12	0.17	672.9	20.19	0.10
671.0	20.13	0.17	673.9	20.22	0.10
671.3	20.14	0.17	674.1	20.22	0.10
672.0	20.16	0.17	674.4	20.23	0.10
672.3	20.17	0.17	674.5	20.23	0.10
680.3	20.41	0.17	680.9	20.43	0.10
680.4	20.41	0.17	681.2	20.44	0.10
680.5	20.41	0.17	681.2	20.44	0.10
680.6	20.42	0.17	681.4	20.44	0.10
680.8	20.42	0.17	681.4	20.44	0.10
680.8	20.42	0.17	681.5	20.44	0.10
681.1	20.43	0.17	681.7	20.45	0.10
681.1	20.43	0.17	681.8	20.45	0.10
690.7	20.72	0.16	696.4	20.89	0.09
690.8	20.72	0.16	696.7	20.90	0.09
690.8	20.73	0.16	697.5	20.92	0.09
691.2	20.73	0.16	697.8	20.93	0.09
691.3	20.74	0.16	698.0	20.94	0.09
691.9	20.76	0.16	698.0	20.94	0.09
692.1	20.76	0.16	698.3	20.95	0.09
692.2	20.77	0.16	698.3	20.95	0.09
707.5	21.22	0.15	708.2	21.25	0.09
707.8	21.23	0.15	708.5	21.26	0.09
707.8	21.24	0.15	709.4	21.28	0.09
707.9	21.24	0.15	709.7	21.29	0.09
708.3	21.25	0.15	709.8	21.30	0.09
708.4	21.25	0.15	710.4	21.31	0.09
708.5	21.26	0.15	711.0	21.33	0.09
708.8	21.26	0.15	711.9	21.36	0.09
713.8	21.41	0.15	717.9	21.54	0.08
714.1	21.42	0.15	718.0	21.54	0.08
714.2	21.43	0.15	718.4	21.55	0.08
714.6	21.44	0.15	718.8	21.56	0.08
714.7	21.44	0.15	719.2	21.58	0.08
714.9	21.45	0.15	719.3	21.58	0.08
714.9	21.45	0.15	719.4	21.58	0.08

715.1	21.45	0.15	719.5	21.58	0.08
729.0	21.87	0.14	724.2	21.73	0.08
730.2	21.91	0.14	724.9	21.75	0.08
730.3	21.91	0.14	725.0	21.75	0.08
730.5	21.91	0.14	726.1	21.78	0.08
730.7	21.92	0.14	726.5	21.80	0.08
730.8	21.92	0.14	727.1	21.81	0.08
731.0	21.93	0.14	727.6	21.83	0.08
731.3	21.94	0.14	728.5	21.86	0.08
755.3	22.66	0.13	757.8	22.73	0.07
756.2	22.69	0.13	758.6	22.76	0.07
756.3	22.69	0.13	758.7	22.76	0.07
756.4	22.69	0.13	758.7	22.76	0.07
756.6	22.70	0.13	759.4	22.78	0.07
756.6	22.70	0.13	759.7	22.79	0.07
756.9	22.71	0.13	759.7	22.79	0.07
757.5	22.72	0.13	759.7	22.79	0.07
757.9	22.74	0.12	759.8	22.79	0.07
758.2	22.75	0.12	760.3	22.81	0.07
758.4	22.75	0.12	760.3	22.81	0.07
758.5	22.75	0.12	760.6	22.82	0.07
758.8	22.76	0.12	760.8	22.83	0.07
759.4	22.78	0.12	760.9	22.83	0.07
759.8	22.79	0.12	761.0	22.83	0.07
759.8	22.79	0.12	761.6	22.85	0.07
803.9	24.12	0.10	803.2	24.10	0.06
804.0	24.12	0.10	803.5	24.10	0.06
804.5	24.13	0.10	803.6	24.11	0.06
804.5	24.14	0.10	803.7	24.11	0.06
804.6	24.14	0.10	803.8	24.11	0.06
804.9	24.15	0.10	803.8	24.12	0.06
805.1	24.15	0.10	803.9	24.12	0.06
805.4	24.16	0.10	804.2	24.13	0.06
810.2	24.31	0.10	811.7	24.35	0.05
811.0	24.33	0.10	811.8	24.35	0.05
811.0	24.33	0.10	812.1	24.36	0.05
811.0	24.33	0.10	812.7	24.38	0.05
811.1	24.33	0.10	812.8	24.38	0.05
811.4	24.34	0.10	813.4	24.40	0.05
812.2	24.37	0.10	814.0	24.42	0.05
812.8	24.38	0.10	814.2	24.43	0.05
816.4	24.49	0.10	820.3	24.61	0.05
816.9	24.51	0.10	820.6	24.62	0.05
817.0	24.51	0.10	820.9	24.63	0.05
817.2	24.51	0.10	821.0	24.63	0.05
817.7	24.53	0.10	821.1	24.63	0.05

817.7	24.53	0.10	821.2	24.64	0.05
817.8	24.53	0.10	821.4	24.64	0.05
818.3	24.55	0.10	821.4	24.64	0.05
825.4	24.76	0.10	826.5	24.79	0.05
825.7	24.77	0.10	827.3	24.82	0.05
826.1	24.78	0.10	827.9	24.84	0.05
826.5	24.79	0.10	828.7	24.86	0.05
826.7	24.80	0.10	830.4	24.91	0.05
826.8	24.80	0.10	830.6	24.92	0.05
827.7	24.83	0.10	831.3	24.94	0.05
827.8	24.83	0.10	831.6	24.95	0.05
912.1	27.36	0.07	913.5	27.41	0.03
912.2	27.37	0.07	913.7	27.41	0.03
912.5	27.37	0.07	913.8	27.41	0.03
912.7	27.38	0.07	913.9	27.42	0.03
912.7	27.38	0.07	914.1	27.42	0.03
912.8	27.38	0.07	914.2	27.42	0.03
912.8	27.39	0.07	914.4	27.43	0.03
912.9	27.39	0.07	914.6	27.44	0.03
914.3	27.43	0.07	914.6	27.44	0.03
914.4	27.43	0.07	914.9	27.45	0.03
914.6	27.44	0.07	915.2	27.46	0.03
914.7	27.44	0.07	915.2	27.46	0.03
914.8	27.44	0.07	915.7	27.47	0.03
914.9	27.45	0.07	915.9	27.48	0.03
915.3	27.46	0.07	916.3	27.49	0.03
915.5	27.47	0.07	916.3	27.49	0.03
925.4	27.76	0.06	928.7	27.86	0.03
925.7	27.77	0.06	928.8	27.86	0.03
925.8	27.77	0.06	928.9	27.87	0.03
925.9	27.78	0.06	929.0	27.87	0.03
926.0	27.78	0.06	929.3	27.88	0.03
926.3	27.79	0.06	929.6	27.89	0.03
926.3	27.79	0.06	929.8	27.89	0.03
926.3	27.79	0.06	930.0	27.90	0.03
953.0	28.59	0.06	961.3	28.84	0.03
953.4	28.60	0.06	962.2	28.87	0.03
953.8	28.61	0.06	962.2	28.87	0.03
954.0	28.62	0.06	962.5	28.88	0.03
954.8	28.64	0.06	962.7	28.88	0.03
955.0	28.65	0.06	962.9	28.89	0.03
955.8	28.68	0.06	963.1	28.89	0.03
956.1	28.68	0.06	963.3	28.90	0.03
973.8	29.22	0.05	976.5	29.29	0.02
974.0	29.22	0.05	976.6	29.30	0.02
974.2	29.23	0.05	976.7	29.30	0.02

974.6	29.24	0.05	976.7	29.30	0.02
975.4	29.26	0.05	976.7	29.30	0.02
975.5	29.26	0.05	976.8	29.30	0.02
975.6	29.27	0.05	977.1	29.31	0.02
975.8	29.27	0.05	977.4	29.32	0.02
984.1	29.52	0.05	986.3	29.59	0.02
984.2	29.52	0.05	986.4	29.59	0.02
984.3	29.53	0.05	986.5	29.59	0.02
984.4	29.53	0.05	986.7	29.60	0.02
984.5	29.54	0.05	986.8	29.61	0.02
984.6	29.54	0.05	986.9	29.61	0.02
985.6	29.57	0.05	987.0	29.61	0.02
985.7	29.57	0.05	987.1	29.61	0.02
1009.5	30.28	0.05	1013.7	30.41	0.02
1010.0	30.30	0.05	1013.9	30.42	0.02
1010.4	30.31	0.05	1013.9	30.42	0.02
1010.4	30.31	0.05	1014.1	30.42	0.02
1010.4	30.31	0.05	1014.2	30.43	0.02
1010.6	30.32	0.05	1014.3	30.43	0.02
1010.7	30.32	0.05	1014.6	30.44	0.02
1010.7	30.32	0.05	1014.7	30.44	0.02
1037.1	31.11	0.04	1037.2	31.11	0.02
1037.2	31.12	0.04	1037.2	31.12	0.02
1037.3	31.12	0.04	1037.4	31.12	0.02
1037.7	31.13	0.04	1037.5	31.13	0.02
1038.3	31.15	0.04	1037.6	31.13	0.02
1038.4	31.15	0.04	1037.6	31.13	0.02
1039.1	31.17	0.04	1038.7	31.16	0.02
1039.1	31.17	0.04	1038.9	31.17	0.02
1044.7	31.34	0.04	1042.8	31.28	0.02
1045.1	31.35	0.04	1043.2	31.29	0.02
1045.3	31.36	0.04	1043.3	31.30	0.02
1045.7	31.37	0.04	1044.0	31.32	0.02
1045.7	31.37	0.04	1044.1	31.32	0.02
1046.0	31.38	0.04	1044.6	31.34	0.02
1046.0	31.38	0.04	1044.6	31.34	0.02
1046.4	31.39	0.04	1045.3	31.36	0.02
1047.0	31.41	0.04	1048.4	31.45	0.02
1048.6	31.46	0.04	1048.9	31.47	0.02
1051.0	31.53	0.04	1049.0	31.47	0.02
1052.4	31.57	0.04	1049.1	31.47	0.02
1053.1	31.59	0.04	1049.2	31.47	0.02
1053.3	31.60	0.04	1049.3	31.48	0.02
1053.7	31.61	0.04	1049.5	31.49	0.02
1054.3	31.63	0.04	1049.6	31.49	0.02
1054.5	31.64	0.04	1053.3	31.60	0.02

1055.2	31.66	0.04	1056.5	31.70	0.02
1055.5	31.66	0.04	1056.7	31.70	0.02
1056.8	31.70	0.04	1057.1	31.71	0.02
1058.5	31.75	0.04	1058.0	31.74	0.02
1059.8	31.79	0.04	1059.5	31.78	0.02
1059.8	31.79	0.04	1059.6	31.79	0.02
1060.1	31.80	0.04	1061.6	31.85	0.02
1066.8	32.00	0.04	1069.1	32.07	0.02
1068.0	32.04	0.04	1069.4	32.08	0.02
1068.0	32.04	0.04	1070.0	32.10	0.02
1068.1	32.04	0.04	1071.7	32.15	0.01
1068.4	32.05	0.04	1079.6	32.39	0.01
1070.2	32.11	0.04	1080.4	32.41	0.01
1071.1	32.13	0.04	1080.7	32.42	0.01
1072.2	32.17	0.04	1080.9	32.43	0.01
1103.0	33.09	0.03	1105.1	33.15	0.01
1103.1	33.09	0.03	1105.3	33.16	0.01
1103.1	33.09	0.03	1105.3	33.16	0.01
1103.1	33.09	0.03	1105.4	33.16	0.01
1103.2	33.10	0.03	1105.5	33.16	0.01
1103.2	33.10	0.03	1105.5	33.16	0.01
1103.5	33.10	0.03	1105.5	33.16	0.01
1103.5	33.11	0.03	1105.6	33.17	0.01
1106.6	33.20	0.03	1107.7	33.23	0.01
1106.6	33.20	0.03	1107.8	33.23	0.01
1106.9	33.21	0.03	1108.1	33.24	0.01
1107.0	33.21	0.03	1108.3	33.25	0.01
1107.3	33.22	0.03	1108.7	33.26	0.01
1107.3	33.22	0.03	1109.0	33.27	0.01
1107.8	33.23	0.03	1109.2	33.28	0.01
1108.4	33.25	0.03	1109.3	33.28	0.01
1123.5	33.70	0.03	1119.2	33.58	0.01
1123.8	33.71	0.03	1119.3	33.58	0.01
1124.8	33.74	0.03	1119.4	33.58	0.01
1124.8	33.74	0.03	1119.5	33.59	0.01
1124.8	33.75	0.03	1119.7	33.59	0.01
1125.1	33.75	0.03	1119.8	33.59	0.01
1125.2	33.76	0.03	1119.8	33.59	0.01
1125.2	33.76	0.03	1119.8	33.59	0.01
1125.4	33.76	0.03	1123.4	33.70	0.01
1125.5	33.77	0.03	1123.8	33.71	0.01
1126.1	33.78	0.03	1123.9	33.72	0.01
1126.1	33.78	0.03	1124.1	33.72	0.01
1126.1	33.78	0.03	1124.3	33.73	0.01
1126.4	33.79	0.03	1124.4	33.73	0.01
1126.8	33.80	0.03	1124.5	33.73	0.01

1126.8	33.80	0.03	1124.5	33.74	0.01
1142.1	34.26	0.03	1131.9	33.96	0.01
1142.2	34.27	0.03	1131.9	33.96	0.01
1142.3	34.27	0.03	1132.0	33.96	0.01
1143.1	34.29	0.03	1132.2	33.97	0.01
1143.3	34.30	0.03	1132.2	33.97	0.01
1143.5	34.31	0.03	1132.2	33.97	0.01
1143.6	34.31	0.03	1132.7	33.98	0.01
1143.7	34.31	0.03	1132.8	33.98	0.01
1147.1	34.41	0.03	1145.0	34.35	0.01
1147.3	34.42	0.03	1145.0	34.35	0.01
1147.8	34.43	0.03	1145.0	34.35	0.01
1147.9	34.44	0.03	1145.2	34.36	0.01
1148.1	34.44	0.03	1145.7	34.37	0.01
1148.2	34.45	0.03	1146.1	34.38	0.01
1148.3	34.45	0.03	1146.2	34.39	0.01
1148.5	34.45	0.03	1146.6	34.40	0.01
1169.0	35.07	0.02	1175.0	35.25	0.01
1169.4	35.08	0.02	1175.2	35.25	0.01
1169.5	35.08	0.02	1175.2	35.26	0.01
1169.5	35.09	0.02	1175.2	35.26	0.01
1169.6	35.09	0.02	1175.4	35.26	0.01
1169.7	35.09	0.02	1175.6	35.27	0.01
1169.8	35.09	0.02	1175.6	35.27	0.01
1170.3	35.11	0.02	1175.7	35.27	0.01
1186.7	35.60	0.02	1183.1	35.49	0.01
1186.8	35.60	0.02	1183.7	35.51	0.01
1186.8	35.60	0.02	1184.2	35.53	0.01
1187.2	35.61	0.02	1184.4	35.53	0.01
1187.3	35.62	0.02	1184.5	35.54	0.01
1187.4	35.62	0.02	1184.6	35.54	0.01
1187.7	35.63	0.02	1184.7	35.54	0.01
1187.8	35.63	0.02	1184.8	35.54	0.01
1191.7	35.75	0.02	1194.3	35.83	0.01
1191.8	35.75	0.02	1194.4	35.83	0.01
1191.9	35.76	0.02	1194.5	35.84	0.01
1192.1	35.76	0.02	1194.7	35.84	0.01
1192.4	35.77	0.02	1195.1	35.85	0.01
1192.5	35.78	0.02	1195.2	35.86	0.01
1192.9	35.79	0.02	1195.4	35.86	0.01
1193.5	35.80	0.02	1195.4	35.86	0.01
1212.4	36.37	0.02	1208.4	36.25	0.01
1212.5	36.37	0.02	1208.4	36.25	0.01
1212.6	36.38	0.02	1208.7	36.26	0.01
1212.7	36.38	0.02	1208.8	36.27	0.01
1213.0	36.39	0.02	1208.9	36.27	0.01

1213.1	36.39	0.02	1209.0	36.27	0.01
1213.4	36.40	0.02	1209.5	36.28	0.01
1214.8	36.44	0.02	1209.8	36.30	0.01
1219.1	36.57	0.02	1226.2	36.79	0.01
1219.8	36.59	0.02	1226.5	36.80	0.01
1221.5	36.65	0.02	1228.4	36.85	0.01
1221.7	36.65	0.02	1229.1	36.87	0.01
1222.0	36.66	0.02	1229.7	36.89	0.01
1222.1	36.66	0.02	1230.3	36.91	0.01
1223.5	36.70	0.02	1232.0	36.96	0.01
1223.6	36.71	0.02	1232.5	36.98	0.01
1235.1	37.05	0.02	1234.0	37.02	0.01
1235.2	37.05	0.02	1234.1	37.02	0.01
1235.2	37.05	0.02	1234.3	37.03	0.01
1235.3	37.06	0.02	1235.3	37.06	0.01
1235.4	37.06	0.02	1235.9	37.08	0.01
1235.4	37.06	0.02	1236.1	37.08	0.01
1235.5	37.06	0.02	1236.2	37.09	0.01
1235.8	37.07	0.02	1237.5	37.12	0.01
1241.1	37.23	0.02	1244.6	37.34	0.01
1241.7	37.25	0.02	1244.8	37.35	0.01
1241.7	37.25	0.02	1245.1	37.35	0.01
1242.2	37.27	0.02	1245.4	37.36	0.01
1242.7	37.28	0.02	1245.4	37.36	0.01
1242.8	37.28	0.02	1245.5	37.36	0.01
1243.8	37.32	0.02	1245.5	37.37	0.01
1243.9	37.32	0.02	1245.6	37.37	0.01
1243.9	37.32	0.02	1251.5	37.55	0.01
1244.2	37.33	0.02	1251.9	37.56	0.01
1244.6	37.34	0.02	1253.0	37.59	0.01
1244.9	37.35	0.02	1253.1	37.59	0.01
1246.8	37.40	0.02	1253.9	37.62	0.01
1246.8	37.40	0.02	1254.0	37.62	0.01
1247.5	37.43	0.02	1255.5	37.66	0.01
1247.8	37.43	0.02	1256.1	37.68	0.01
1274.7	38.24	0.02	1278.0	38.34	0.01
1274.7	38.24	0.02	1278.2	38.35	0.01
1274.9	38.25	0.02	1278.2	38.35	0.01
1274.9	38.25	0.02	1278.3	38.35	0.01
1274.9	38.25	0.02	1278.3	38.35	0.01
1275.0	38.25	0.02	1278.3	38.35	0.01
1275.1	38.25	0.02	1278.5	38.36	0.01
1275.1	38.25	0.02	1278.7	38.36	0.01
1299.8	38.99	0.01	1297.5	38.93	0.00
1299.9	39.00	0.01	1297.7	38.93	0.00
1300.0	39.00	0.01	1297.8	38.93	0.00

1300.2	39.01	0.01	1297.8	38.94	0.00
1300.2	39.01	0.01	1298.3	38.95	0.00
1300.3	39.01	0.01	1298.5	38.96	0.00
1300.3	39.01	0.01	1298.7	38.96	0.00
1300.5	39.02	0.01	1298.9	38.97	0.00
1307.8	39.23	0.01	1310.1	39.30	0.00
1308.3	39.25	0.01	1310.5	39.31	0.00
1308.4	39.25	0.01	1310.6	39.32	0.00
1308.6	39.26	0.01	1310.6	39.32	0.00
1308.6	39.26	0.01	1310.7	39.32	0.00
1308.7	39.26	0.01	1310.7	39.32	0.00
1308.7	39.26	0.01	1310.9	39.33	0.00
1308.9	39.27	0.01	1311.4	39.34	0.00
1318.3	39.55	0.01	1323.5	39.70	0.00
1318.5	39.56	0.01	1323.5	39.70	0.00
1318.6	39.56	0.01	1323.8	39.71	0.00
1318.6	39.56	0.01	1323.8	39.71	0.00
1318.7	39.56	0.01	1323.9	39.72	0.00
1318.8	39.56	0.01	1324.0	39.72	0.00
1319.0	39.57	0.01	1324.1	39.72	0.00
1319.1	39.57	0.01	1324.7	39.74	0.00
1350.8	40.53	0.01	1347.7	40.43	0.00
1350.9	40.53	0.01	1348.2	40.45	0.00
1351.1	40.53	0.01	1348.4	40.45	0.00
1351.2	40.54	0.01	1348.4	40.45	0.00
1351.7	40.55	0.01	1348.5	40.46	0.00
1351.8	40.55	0.01	1348.6	40.46	0.00
1351.9	40.56	0.01	1348.6	40.46	0.00
1351.9	40.56	0.01	1348.6	40.46	0.00
1360.2	40.81	0.01	1370.8	41.12	0.00
1360.8	40.82	0.01	1370.9	41.13	0.00
1361.3	40.84	0.01	1370.9	41.13	0.00
1361.6	40.85	0.01	1370.9	41.13	0.00
1363.9	40.92	0.01	1371.9	41.16	0.00
1364.5	40.94	0.01	1372.0	41.16	0.00
1364.6	40.94	0.01	1372.3	41.17	0.00
1365.6	40.97	0.01	1372.5	41.17	0.00
1368.8	41.06	0.01	1378.2	41.35	0.00
1370.0	41.10	0.01	1378.4	41.35	0.00
1370.6	41.12	0.01	1378.6	41.36	0.00
1371.0	41.13	0.01	1379.1	41.37	0.00
1372.5	41.18	0.01	1379.5	41.39	0.00
1372.6	41.18	0.01	1379.6	41.39	0.00
1372.8	41.18	0.01	1380.0	41.40	0.00
1373.2	41.20	0.01	1380.4	41.41	0.00
1390.5	41.72	0.01	1383.9	41.52	0.00

1390.6	41.72	0.01	1384.3	41.53	0.00
1390.7	41.72	0.01	1384.3	41.53	0.00
1390.7	41.72	0.01	1384.4	41.53	0.00
1390.7	41.72	0.01	1384.5	41.53	0.00
1390.8	41.72	0.01	1384.6	41.54	0.00
1391.0	41.73	0.01	1384.9	41.55	0.00
1391.2	41.73	0.01	1385.5	41.57	0.00
1400.6	42.02	0.01	1396.2	41.89	0.00
1401.3	42.04	0.01	1396.5	41.89	0.00
1401.4	42.04	0.01	1396.6	41.90	0.00
1401.4	42.04	0.01	1397.1	41.91	0.00
1401.5	42.05	0.01	1398.4	41.95	0.00
1401.7	42.05	0.01	1398.6	41.96	0.00
1401.9	42.06	0.01	1400.7	42.02	0.00
1401.9	42.06	0.01	1400.9	42.03	0.00
1409.3	42.28	0.01	1410.7	42.32	0.00
1409.4	42.28	0.01	1411.2	42.34	0.00
1409.4	42.28	0.01	1411.3	42.34	0.00
1409.6	42.29	0.01	1411.4	42.34	0.00
1409.9	42.30	0.01	1412.3	42.37	0.00
1410.2	42.31	0.01	1412.5	42.38	0.00
1410.7	42.32	0.01	1412.7	42.38	0.00
1410.8	42.32	0.01	1412.9	42.39	0.00
1412.4	42.37	0.01	1413.3	42.40	0.00
1412.5	42.38	0.01	1413.5	42.40	0.00
1412.7	42.38	0.01	1413.9	42.42	0.00
1412.8	42.38	0.01	1414.1	42.42	0.00
1413.0	42.39	0.01	1414.2	42.43	0.00
1413.3	42.40	0.01	1414.4	42.43	0.00
1413.3	42.40	0.01	1415.0	42.45	0.00
1413.5	42.40	0.01	1415.4	42.46	0.00
1418.1	42.54	0.01	1420.2	42.61	0.00
1418.3	42.55	0.01	1420.3	42.61	0.00
1418.4	42.55	0.01	1420.3	42.61	0.00
1418.6	42.56	0.01	1420.4	42.61	0.00
1418.7	42.56	0.01	1420.5	42.61	0.00
1419.1	42.57	0.01	1421.0	42.63	0.00
1419.2	42.58	0.01	1421.3	42.64	0.00
1419.8	42.59	0.01	1422.0	42.66	0.00
1437.9	43.14	0.01	1442.2	43.27	0.00
1438.6	43.16	0.01	1442.6	43.28	0.00
1438.7	43.16	0.01	1442.8	43.28	0.00
1438.9	43.17	0.01	1443.0	43.29	0.00
1438.9	43.17	0.01	1443.2	43.30	0.00
1439.3	43.18	0.01	1443.3	43.30	0.00
1440.1	43.20	0.01	1444.7	43.34	0.00

1440.4	43.21	0.01	1445.0	43.35	0.00
1453.1	43.59	0.01	1449.8	43.49	0.00
1453.5	43.60	0.01	1450.1	43.50	0.00
1453.6	43.61	0.01	1450.3	43.51	0.00
1453.7	43.61	0.01	1450.4	43.51	0.00
1453.9	43.62	0.01	1450.6	43.52	0.00
1454.3	43.63	0.01	1450.6	43.52	0.00
1454.4	43.63	0.01	1450.8	43.52	0.00
1455.1	43.65	0.01	1451.2	43.54	0.00
1455.7	43.67	0.01	1459.9	43.80	0.00
1456.0	43.68	0.01	1460.4	43.81	0.00
1456.8	43.70	0.01	1460.4	43.81	0.00
1456.8	43.70	0.01	1461.0	43.83	0.00
1456.9	43.71	0.01	1461.2	43.84	0.00
1456.9	43.71	0.01	1461.5	43.85	0.00
1457.8	43.73	0.01	1461.8	43.85	0.00
1458.9	43.77	0.01	1462.5	43.88	0.00
1459.4	43.78	0.01	1463.0	43.89	0.00
1459.6	43.79	0.01	1464.0	43.92	0.00
1460.6	43.82	0.01	1464.0	43.92	0.00
1460.8	43.82	0.01	1464.3	43.93	0.00
1460.9	43.83	0.01	1464.4	43.93	0.00
1461.1	43.83	0.01	1464.6	43.94	0.00
1463.2	43.89	0.01	1464.7	43.94	0.00
1463.4	43.90	0.01	1464.8	43.95	0.00
1463.5	43.91	0.01	1465.7	43.97	0.00
1463.9	43.92	0.01	1465.8	43.97	0.00
1464.1	43.92	0.01	1466.0	43.98	0.00
1464.3	43.93	0.01	1466.7	44.00	0.00
1464.7	43.94	0.01	1472.8	44.18	0.00
1464.7	43.94	0.01	1472.9	44.19	0.00
1465.0	43.95	0.01	1473.0	44.19	0.00
1465.5	43.96	0.01	1473.1	44.19	0.00
1470.3	44.11	0.01	1473.1	44.19	0.00
1471.8	44.15	0.01	1473.9	44.22	0.00
1472.0	44.16	0.01	1474.0	44.22	0.00
1472.2	44.17	0.01	1474.5	44.24	0.00
1472.5	44.17	0.01	1474.8	44.24	0.00
1473.8	44.21	0.01	1475.1	44.25	0.00
1474.6	44.24	0.01	1475.4	44.26	0.00
1475.2	44.25	0.01	1475.6	44.27	0.00
1477.4	44.32	0.01	1475.7	44.27	0.00
1478.2	44.35	0.01	1475.7	44.27	0.00
1478.2	44.35	0.01	1476.6	44.30	0.00
1478.6	44.36	0.01	1476.8	44.30	0.00
1480.3	44.41	0.01	1477.0	44.31	0.00

1480.3	44.41	0.01	1477.7	44.33	0.00
1481.1	44.43	0.01	1477.8	44.34	0.00
1481.3	44.44	0.01	1477.9	44.34	0.00
1491.0	44.73	0.01	1496.0	44.88	0.00
1491.0	44.73	0.01	1496.2	44.89	0.00
1491.1	44.73	0.01	1496.4	44.89	0.00
1491.1	44.73	0.01	1496.4	44.89	0.00
1491.1	44.73	0.01	1496.4	44.89	0.00
1491.2	44.74	0.01	1496.4	44.89	0.00
1491.3	44.74	0.01	1496.8	44.90	0.00
1491.7	44.75	0.01	1496.9	44.91	0.00
1544.6	46.34	0.01	1547.6	46.43	0.00
1545.0	46.35	0.01	1547.7	46.43	0.00
1545.0	46.35	0.01	1547.9	46.44	0.00
1545.2	46.36	0.01	1547.9	46.44	0.00
1545.5	46.36	0.01	1547.9	46.44	0.00
1545.6	46.37	0.01	1547.9	46.44	0.00
1545.6	46.37	0.01	1547.9	46.44	0.00
1545.8	46.37	0.01	1548.0	46.44	0.00
1590.8	47.72	0.00	1594.6	47.84	0.00
1591.5	47.75	0.00	1594.6	47.84	0.00
1591.6	47.75	0.00	1594.7	47.84	0.00
1591.7	47.75	0.00	1594.7	47.84	0.00
1591.8	47.75	0.00	1594.9	47.85	0.00
1591.9	47.76	0.00	1594.9	47.85	0.00
1592.1	47.76	0.00	1595.0	47.85	0.00
1592.3	47.77	0.00	1595.2	47.86	0.00
1595.6	47.87	0.00	1599.3	47.98	0.00
1595.7	47.87	0.00	1599.4	47.98	0.00
1595.7	47.87	0.00	1599.4	47.98	0.00
1595.8	47.87	0.00	1599.7	47.99	0.00
1595.9	47.88	0.00	1599.7	47.99	0.00
1596.1	47.88	0.00	1599.7	47.99	0.00
1596.2	47.89	0.00	1599.8	47.99	0.00
1596.6	47.90	0.00	1599.9	48.00	0.00
1617.9	48.54	0.00	1620.5	48.62	0.00
1617.9	48.54	0.00	1620.5	48.62	0.00
1617.9	48.54	0.00	1620.5	48.62	0.00
1617.9	48.54	0.00	1620.7	48.62	0.00
1618.0	48.54	0.00	1620.7	48.62	0.00
1618.0	48.54	0.00	1620.8	48.62	0.00
1618.0	48.54	0.00	1620.9	48.63	0.00
1618.1	48.54	0.00	1621.0	48.63	0.00
1636.8	49.10	0.00	1645.4	49.36	0.00
1636.9	49.11	0.00	1645.6	49.37	0.00
1637.0	49.11	0.00	1645.6	49.37	0.00

1637.3	49.12	0.00	1645.7	49.37	0.00
1637.3	49.12	0.00	1645.8	49.37	0.00
1637.3	49.12	0.00	1645.8	49.37	0.00
1637.5	49.12	0.00	1646.2	49.39	0.00
1637.5	49.12	0.00	1646.2	49.39	0.00
1650.6	49.52	0.00	1651.5	49.54	0.00
1650.8	49.52	0.00	1651.6	49.55	0.00
1650.9	49.53	0.00	1651.9	49.56	0.00
1651.1	49.53	0.00	1652.2	49.57	0.00
1651.1	49.53	0.00	1652.8	49.58	0.00
1651.1	49.53	0.00	1652.8	49.59	0.00
1651.2	49.54	0.00	1652.9	49.59	0.00
1651.3	49.54	0.00	1653.1	49.59	0.00
1653.0	49.59	0.00	1668.2	50.04	0.00
1653.1	49.59	0.00	1668.8	50.06	0.00
1653.2	49.60	0.00	1668.9	50.07	0.00
1653.2	49.60	0.00	1669.0	50.07	0.00
1653.2	49.60	0.00	1669.2	50.08	0.00
1653.3	49.60	0.00	1669.3	50.08	0.00
1653.5	49.61	0.00	1669.3	50.08	0.00
1653.5	49.61	0.00	1669.6	50.09	0.00
1658.1	49.74	0.00	1669.9	50.10	0.00
1658.1	49.74	0.00	1670.0	50.10	0.00
1658.6	49.76	0.00	1670.2	50.11	0.00
1658.9	49.77	0.00	1670.3	50.11	0.00
1658.9	49.77	0.00	1670.3	50.11	0.00
1658.9	49.77	0.00	1670.7	50.12	0.00
1658.9	49.77	0.00	1670.8	50.12	0.00
1659.0	49.77	0.00	1670.8	50.12	0.00
1669.7	50.09	0.00	1678.4	50.35	0.00
1669.9	50.10	0.00	1678.5	50.35	0.00
1669.9	50.10	0.00	1678.5	50.35	0.00
1670.0	50.10	0.00	1678.6	50.36	0.00
1670.2	50.11	0.00	1678.7	50.36	0.00
1670.3	50.11	0.00	1678.8	50.36	0.00
1670.4	50.11	0.00	1678.8	50.36	0.00
1670.6	50.12	0.00	1678.9	50.37	0.00
1684.5	50.54	0.00	1687.4	50.62	0.00
1684.7	50.54	0.00	1687.6	50.63	0.00
1684.8	50.54	0.00	1687.6	50.63	0.00
1685.2	50.55	0.00	1688.0	50.64	0.00
1685.2	50.56	0.00	1688.1	50.64	0.00
1685.3	50.56	0.00	1688.1	50.64	0.00
1685.3	50.56	0.00	1688.2	50.65	0.00
1685.6	50.57	0.00	1688.2	50.65	0.00
1696.4	50.89	0.00	1698.5	50.95	0.00

1696.5	50.90	0.00	1698.5	50.95	0.00
1697.0	50.91	0.00	1698.6	50.96	0.00
1697.1	50.91	0.00	1698.6	50.96	0.00
1697.6	50.93	0.00	1699.8	50.99	0.00
1697.6	50.93	0.00	1699.9	51.00	0.00
1697.9	50.94	0.00	1700.1	51.00	0.00
1697.9	50.94	0.00	1700.2	51.00	0.00
1708.1	51.24	0.00	1712.8	51.38	0.00
1708.5	51.25	0.00	1712.9	51.39	0.00
1708.5	51.25	0.00	1713.0	51.39	0.00
1708.6	51.26	0.00	1713.1	51.39	0.00
1709.0	51.27	0.00	1714.1	51.42	0.00
1709.2	51.28	0.00	1714.2	51.43	0.00
1709.7	51.29	0.00	1714.3	51.43	0.00
1709.9	51.30	0.00	1714.4	51.43	0.00
1740.9	52.23	0.00	1742.2	52.27	0.00
1740.9	52.23	0.00	1742.3	52.27	0.00
1741.0	52.23	0.00	1742.4	52.27	0.00
1741.0	52.23	0.00	1742.4	52.27	0.00
1741.4	52.24	0.00	1742.7	52.28	0.00
1741.7	52.25	0.00	1742.9	52.29	0.00
1741.7	52.25	0.00	1743.1	52.29	0.00
1742.0	52.26	0.00	1743.3	52.30	0.00
1766.7	53.00	0.00	1768.5	53.05	0.00
1767.3	53.02	0.00	1768.6	53.06	0.00
1767.6	53.03	0.00	1768.6	53.06	0.00
1767.7	53.03	0.00	1769.1	53.07	0.00
1767.7	53.03	0.00	1769.2	53.08	0.00
1768.3	53.05	0.00	1769.3	53.08	0.00
1768.4	53.05	0.00	1769.4	53.08	0.00
1768.7	53.06	0.00	1769.5	53.09	0.00
1776.0	53.28	0.00	1775.2	53.25	0.00
1776.1	53.28	0.00	1775.3	53.26	0.00
1776.4	53.29	0.00	1775.3	53.26	0.00
1776.6	53.30	0.00	1775.3	53.26	0.00
1776.6	53.30	0.00	1775.4	53.26	0.00
1776.8	53.30	0.00	1775.4	53.26	0.00
1777.0	53.31	0.00	1775.5	53.26	0.00
1777.0	53.31	0.00	1775.5	53.26	0.00
1789.6	53.69	0.00	1790.3	53.71	0.00
1789.9	53.70	0.00	1790.4	53.71	0.00
1789.9	53.70	0.00	1790.8	53.72	0.00
1790.1	53.70	0.00	1790.8	53.72	0.00
1790.1	53.70	0.00	1790.8	53.72	0.00
1790.2	53.70	0.00	1791.0	53.73	0.00
1790.2	53.71	0.00	1791.2	53.74	0.00

1790.3	53.71	0.00	1791.3	53.74	0.00
1790.5	53.71	0.00	1791.4	53.74	0.00
1790.6	53.72	0.00	1791.4	53.74	0.00
1790.7	53.72	0.00	1791.4	53.74	0.00
1790.8	53.72	0.00	1791.5	53.74	0.00
1790.8	53.72	0.00	1791.6	53.75	0.00
1791.0	53.73	0.00	1791.6	53.75	0.00
1791.0	53.73	0.00	1791.8	53.75	0.00
1791.3	53.74	0.00	1791.9	53.76	0.00
1835.1	55.05	0.00	1835.9	55.08	0.00
1835.1	55.05	0.00	1836.0	55.08	0.00
1835.2	55.06	0.00	1836.1	55.08	0.00
1835.3	55.06	0.00	1836.1	55.08	0.00
1835.3	55.06	0.00	1836.1	55.08	0.00
1835.3	55.06	0.00	1836.2	55.09	0.00
1835.5	55.06	0.00	1836.2	55.09	0.00
1835.5	55.06	0.00	1836.2	55.09	0.00
1861.0	55.83	0.00	1862.2	55.87	0.00
1861.0	55.83	0.00	1862.2	55.87	0.00
1861.1	55.83	0.00	1862.3	55.87	0.00
1861.1	55.83	0.00	1862.3	55.87	0.00
1861.2	55.83	0.00	1862.4	55.87	0.00
1861.3	55.84	0.00	1862.4	55.87	0.00
1861.3	55.84	0.00	1862.5	55.87	0.00
1861.4	55.84	0.00	1862.5	55.87	0.00
1865.4	55.96	0.00	1867.1	56.01	0.00
1865.5	55.97	0.00	1867.1	56.01	0.00
1865.6	55.97	0.00	1867.2	56.02	0.00
1865.8	55.97	0.00	1867.2	56.02	0.00
1865.9	55.98	0.00	1867.5	56.03	0.00
1865.9	55.98	0.00	1867.6	56.03	0.00
1866.0	55.98	0.00	1867.7	56.03	0.00
1866.2	55.99	0.00	1867.8	56.03	0.00
1868.2	56.04	0.00	1869.3	56.08	0.00
1868.3	56.05	0.00	1869.4	56.08	0.00
1868.3	56.05	0.00	1869.5	56.09	0.00
1868.3	56.05	0.00	1869.7	56.09	0.00
1868.4	56.05	0.00	1869.7	56.09	0.00
1868.4	56.05	0.00	1869.9	56.10	0.00
1868.4	56.05	0.00	1869.9	56.10	0.00
1868.5	56.06	0.00	1869.9	56.10	0.00
1872.8	56.18	0.00	1878.9	56.37	0.00
1872.8	56.18	0.00	1879.0	56.37	0.00
1872.9	56.19	0.00	1879.5	56.39	0.00
1873.0	56.19	0.00	1879.6	56.39	0.00
1873.1	56.19	0.00	1879.9	56.40	0.00

1873.4	56.20	0.00	1880.0	56.40	0.00
1873.4	56.20	0.00	1880.3	56.41	0.00
1873.4	56.20	0.00	1880.4	56.41	0.00
1883.2	56.50	0.00	1884.5	56.54	0.00
1883.3	56.50	0.00	1884.7	56.54	0.00
1883.4	56.50	0.00	1884.8	56.54	0.00
1883.7	56.51	0.00	1884.9	56.55	0.00
1883.7	56.51	0.00	1884.9	56.55	0.00
1883.8	56.51	0.00	1885.1	56.55	0.00
1883.8	56.51	0.00	1885.1	56.55	0.00
1883.9	56.52	0.00	1885.2	56.56	0.00
1919.1	57.57	0.00	1920.4	57.61	0.00
1919.3	57.58	0.00	1920.5	57.61	0.00
1919.6	57.59	0.00	1920.7	57.62	0.00
1919.6	57.59	0.00	1920.9	57.63	0.00
1919.6	57.59	0.00	1920.9	57.63	0.00
1919.9	57.60	0.00	1921.0	57.63	0.00
1919.9	57.60	0.00	1921.0	57.63	0.00
1920.0	57.60	0.00	1921.1	57.63	0.00
1927.2	57.81	0.00	1929.7	57.89	0.00
1927.2	57.82	0.00	1929.9	57.90	0.00
1927.2	57.82	0.00	1929.9	57.90	0.00
1927.3	57.82	0.00	1929.9	57.90	0.00
1927.4	57.82	0.00	1929.9	57.90	0.00
1927.5	57.83	0.00	1930.0	57.90	0.00
1927.6	57.83	0.00	1930.0	57.90	0.00
1927.7	57.83	0.00	1930.2	57.91	0.00
1935.5	58.07	0.00	1946.6	58.40	0.00
1935.7	58.07	0.00	1946.7	58.40	0.00
1935.7	58.07	0.00	1946.9	58.41	0.00
1935.8	58.07	0.00	1947.2	58.42	0.00
1935.9	58.08	0.00	1947.3	58.42	0.00
1936.0	58.08	0.00	1947.4	58.42	0.00
1936.1	58.08	0.00	1947.6	58.43	0.00
1936.4	58.09	0.00	1947.6	58.43	0.00
1950.7	58.52	0.00	1957.3	58.72	0.00
1950.7	58.52	0.00	1957.4	58.72	0.00
1950.8	58.53	0.00	1957.4	58.72	0.00
1950.8	58.53	0.00	1957.5	58.72	0.00
1951.0	58.53	0.00	1957.5	58.73	0.00
1951.1	58.53	0.00	1957.6	58.73	0.00
1951.2	58.53	0.00	1957.6	58.73	0.00
1951.2	58.54	0.00	1957.6	58.73	0.00
1954.3	58.63	0.00	1961.1	58.83	0.00
1954.4	58.63	0.00	1961.3	58.84	0.00
1954.5	58.63	0.00	1961.4	58.84	0.00

1954.5	58.63	0.00	1961.4	58.84	0.00
1954.5	58.64	0.00	1961.4	58.84	0.00
1954.7	58.64	0.00	1961.4	58.84	0.00
1954.8	58.64	0.00	1961.5	58.84	0.00
1954.8	58.64	0.00	1961.6	58.85	0.00
1962.5	58.87	0.00	1964.3	58.93	0.00
1962.6	58.88	0.00	1964.4	58.93	0.00
1962.6	58.88	0.00	1964.6	58.94	0.00
1962.6	58.88	0.00	1964.7	58.94	0.00
1962.7	58.88	0.00	1964.8	58.94	0.00
1962.7	58.88	0.00	1964.8	58.94	0.00
1962.8	58.88	0.00	1964.9	58.95	0.00
1962.8	58.88	0.00	1964.9	58.95	0.00
1996.6	59.90	0.00	1997.3	59.92	0.00
1996.7	59.90	0.00	1997.4	59.92	0.00
1996.7	59.90	0.00	1997.5	59.92	0.00
1996.8	59.90	0.00	1997.5	59.92	0.00
1996.8	59.90	0.00	1997.5	59.93	0.00
1996.8	59.90	0.00	1997.6	59.93	0.00
1996.8	59.91	0.00	1997.6	59.93	0.00
1997.0	59.91	0.00	1997.7	59.93	0.00
2005.8	60.17	0.00	2014.9	60.45	0.00
2005.8	60.17	0.00	2015.0	60.45	0.00
2005.9	60.18	0.00	2015.1	60.45	0.00
2006.1	60.18	0.00	2015.2	60.46	0.00
2006.2	60.18	0.00	2015.3	60.46	0.00
2006.2	60.19	0.00	2015.3	60.46	0.00
2006.2	60.19	0.00	2015.3	60.46	0.00
2006.4	60.19	0.00	2015.4	60.46	0.00
2033.4	61.00	0.00	2047.9	61.44	0.00
2033.5	61.01	0.00	2047.9	61.44	0.00
2033.6	61.01	0.00	2048.0	61.44	0.00
2033.6	61.01	0.00	2048.0	61.44	0.00
2033.6	61.01	0.00	2048.1	61.44	0.00
2033.8	61.01	0.00	2048.1	61.44	0.00
2033.8	61.01	0.00	2048.1	61.44	0.00
2033.8	61.01	0.00	2048.1	61.44	0.00
2922.4	87.67	0.00	2930.3	87.91	0.00
2922.4	87.67	0.00	2930.4	87.91	0.00
2922.5	87.68	0.00	2930.5	87.91	0.00
2922.8	87.68	0.00	2930.5	87.91	0.00
2922.9	87.69	0.00	2930.6	87.92	0.00
2922.9	87.69	0.00	2930.6	87.92	0.00
2922.9	87.69	0.00	2930.6	87.92	0.00
2922.9	87.69	0.00	2930.7	87.92	0.00
2998.2	89.95	0.00	2998.4	89.95	0.00

2998.3	89.95	0.00	2998.5	89.95	0.00
2998.3	89.95	0.00	2998.8	89.96	0.00
2998.6	89.96	0.00	2998.9	89.97	0.00
2998.6	89.96	0.00	2998.9	89.97	0.00
2998.8	89.96	0.00	2999.0	89.97	0.00
2998.8	89.96	0.00	2999.1	89.97	0.00
2998.8	89.96	0.00	2999.1	89.97	0.00
3000.0	90.00	0.00	2999.5	89.99	0.00
3000.2	90.00	0.00	2999.6	89.99	0.00
3000.2	90.01	0.00	3000.0	90.00	0.00
3000.2	90.01	0.00	3000.1	90.00	0.00
3000.5	90.02	0.00	3000.2	90.00	0.00
3000.7	90.02	0.00	3000.2	90.01	0.00
3000.8	90.02	0.00	3000.2	90.01	0.00
3000.8	90.02	0.00	3000.2	90.01	0.00
3001.6	90.05	0.00	3001.7	90.05	0.00
3001.9	90.06	0.00	3001.9	90.06	0.00
3001.9	90.06	0.00	3002.0	90.06	0.00
3002.3	90.07	0.00	3002.1	90.06	0.00
3002.3	90.07	0.00	3002.5	90.07	0.00
3002.7	90.08	0.00	3002.5	90.07	0.00
3002.9	90.09	0.00	3002.6	90.08	0.00
3003.0	90.09	0.00	3002.6	90.08	0.00
3004.4	90.13	0.00	3003.0	90.09	0.00
3004.7	90.14	0.00	3003.1	90.09	0.00
3004.9	90.15	0.00	3003.1	90.09	0.00
3004.9	90.15	0.00	3003.4	90.10	0.00
3005.0	90.15	0.00	3003.8	90.11	0.00
3005.3	90.16	0.00	3003.9	90.12	0.00
3005.4	90.16	0.00	3003.9	90.12	0.00
3005.7	90.17	0.00	3004.0	90.12	0.00
3007.1	90.21	0.00	3008.0	90.24	0.00
3007.3	90.22	0.00	3008.0	90.24	0.00
3007.4	90.22	0.00	3008.0	90.24	0.00
3007.9	90.24	0.00	3008.1	90.24	0.00
3008.1	90.24	0.00	3008.1	90.24	0.00
3008.4	90.25	0.00	3008.2	90.25	0.00
3008.5	90.25	0.00	3008.3	90.25	0.00
3008.6	90.26	0.00	3008.3	90.25	0.00
3009.1	90.27	0.00	3008.9	90.27	0.00
3009.2	90.28	0.00	3009.1	90.27	0.00
3009.2	90.28	0.00	3009.2	90.28	0.00
3009.5	90.29	0.00	3009.2	90.28	0.00
3009.6	90.29	0.00	3010.3	90.31	0.00
3009.8	90.29	0.00	3010.4	90.31	0.00
3009.8	90.29	0.00	3010.5	90.32	0.00

3009.8	90.29	0.00	3010.6	90.32	0.00
3012.1	90.36	0.00	3011.1	90.33	0.00
3012.1	90.36	0.00	3011.1	90.33	0.00
3012.1	90.36	0.00	3011.2	90.33	0.00
3012.2	90.37	0.00	3011.2	90.34	0.00
3012.2	90.37	0.00	3012.0	90.36	0.00
3012.2	90.37	0.00	3012.0	90.36	0.00
3012.3	90.37	0.00	3012.1	90.36	0.00
3012.4	90.37	0.00	3012.2	90.37	0.00
3016.1	90.48	0.00	3012.3	90.37	0.00
3016.5	90.49	0.00	3012.3	90.37	0.00
3016.7	90.50	0.00	3012.3	90.37	0.00
3016.8	90.50	0.00	3012.4	90.37	0.00
3016.8	90.50	0.00	3012.4	90.37	0.00
3016.8	90.50	0.00	3012.5	90.37	0.00
3016.9	90.51	0.00	3012.6	90.38	0.00
3017.0	90.51	0.00	3012.6	90.38	0.00
3017.4	90.52	0.00	3013.9	90.42	0.00
3017.8	90.53	0.00	3013.9	90.42	0.00
3017.8	90.53	0.00	3014.1	90.42	0.00
3017.8	90.53	0.00	3014.1	90.42	0.00
3017.9	90.54	0.00	3015.2	90.46	0.00
3017.9	90.54	0.00	3015.4	90.46	0.00
3017.9	90.54	0.00	3015.5	90.46	0.00
3018.0	90.54	0.00	3015.6	90.47	0.00
3018.4	90.55	0.00	3016.8	90.50	0.00
3018.5	90.55	0.00	3016.8	90.50	0.00
3018.6	90.56	0.00	3016.9	90.51	0.00
3018.7	90.56	0.00	3016.9	90.51	0.00
3018.8	90.56	0.00	3016.9	90.51	0.00
3018.8	90.57	0.00	3016.9	90.51	0.00
3019.6	90.59	0.00	3017.0	90.51	0.00
3019.9	90.60	0.00	3017.0	90.51	0.00
3021.1	90.63	0.00	3022.7	90.68	0.00
3021.1	90.63	0.00	3022.8	90.68	0.00
3021.4	90.64	0.00	3023.1	90.69	0.00
3021.4	90.64	0.00	3023.3	90.70	0.00
3021.6	90.65	0.00	3023.3	90.70	0.00
3021.6	90.65	0.00	3023.4	90.70	0.00
3021.6	90.65	0.00	3023.5	90.71	0.00
3021.7	90.65	0.00	3023.6	90.71	0.00
3021.8	90.65	0.00	3029.2	90.88	0.00
3021.8	90.65	0.00	3029.4	90.88	0.00
3022.2	90.67	0.00	3029.5	90.88	0.00
3022.4	90.67	0.00	3029.6	90.89	0.00
3022.5	90.67	0.00	3030.0	90.90	0.00

3022.7	90.68	0.00	3030.6	90.92	0.00
3022.7	90.68	0.00	3030.7	90.92	0.00
3023.0	90.69	0.00	3030.8	90.92	0.00
3023.4	90.70	0.00	3032.2	90.97	0.00
3023.7	90.71	0.00	3032.3	90.97	0.00
3024.0	90.72	0.00	3032.4	90.97	0.00
3024.0	90.72	0.00	3032.5	90.97	0.00
3024.0	90.72	0.00	3032.5	90.98	0.00
3024.0	90.72	0.00	3032.7	90.98	0.00
3024.0	90.72	0.00	3033.0	90.99	0.00
3024.3	90.73	0.00	3033.1	90.99	0.00
3024.5	90.73	0.00	3033.9	91.02	0.00
3024.5	90.74	0.00	3034.1	91.02	0.00
3024.8	90.74	0.00	3034.1	91.02	0.00
3024.9	90.75	0.00	3034.3	91.03	0.00
3025.1	90.75	0.00	3034.4	91.03	0.00
3025.3	90.76	0.00	3034.7	91.04	0.00
3025.4	90.76	0.00	3034.7	91.04	0.00
3025.6	90.77	0.00	3034.7	91.04	0.00
3026.0	90.78	0.00	3039.0	91.17	0.00
3026.0	90.78	0.00	3039.1	91.17	0.00
3026.5	90.80	0.00	3039.1	91.17	0.00
3026.7	90.80	0.00	3039.2	91.18	0.00
3028.2	90.85	0.00	3039.3	91.18	0.00
3028.4	90.85	0.00	3039.3	91.18	0.00
3028.6	90.86	0.00	3039.3	91.18	0.00
3028.8	90.87	0.00	3039.3	91.18	0.00
3030.5	90.91	0.00	3040.3	91.21	0.00
3031.2	90.94	0.00	3040.3	91.21	0.00
3031.3	90.94	0.00	3040.5	91.22	0.00
3031.3	90.94	0.00	3040.6	91.22	0.00
3031.4	90.94	0.00	3040.6	91.22	0.00
3031.6	90.95	0.00	3040.8	91.22	0.00
3031.7	90.95	0.00	3040.8	91.22	0.00
3031.8	90.95	0.00	3040.9	91.23	0.00
3033.2	90.99	0.00	3041.2	91.24	0.00
3033.2	91.00	0.00	3041.2	91.24	0.00
3033.3	91.00	0.00	3041.4	91.24	0.00
3033.4	91.00	0.00	3041.5	91.24	0.00
3033.4	91.00	0.00	3041.5	91.25	0.00
3033.5	91.00	0.00	3041.5	91.25	0.00
3033.5	91.01	0.00	3041.6	91.25	0.00
3033.6	91.01	0.00	3041.8	91.25	0.00
3035.4	91.06	0.00	3041.8	91.25	0.00
3035.6	91.07	0.00	3041.8	91.25	0.00
3035.6	91.07	0.00	3041.9	91.26	0.00

3035.7	91.07	0.00	3041.9	91.26	0.00
3035.8	91.07	0.00	3042.0	91.26	0.00
3035.8	91.07	0.00	3042.1	91.26	0.00
3036.1	91.08	0.00	3042.1	91.26	0.00
3036.2	91.09	0.00	3042.1	91.26	0.00
3041.3	91.24	0.00	3045.0	91.35	0.00
3041.4	91.24	0.00	3045.0	91.35	0.00
3041.5	91.24	0.00	3045.0	91.35	0.00
3041.5	91.24	0.00	3045.1	91.35	0.00
3041.5	91.25	0.00	3045.1	91.35	0.00
3041.6	91.25	0.00	3045.1	91.35	0.00
3041.7	91.25	0.00	3045.1	91.35	0.00
3041.7	91.25	0.00	3045.1	91.35	0.00

Table S3. Experimental details for the acquisition of the ^{13}C CP/MAS NMR spectra

	Cooling Down 308 K	Cooling Down 280 K	All Others
B_0 (T)	14.1	14.1	14.1
Time domain size	4096	4096	4096
Dwell time (μs)	3.6	3.6	3.6
Acquisition time (ms)	14.7	14.7	14.7
Spectral width (kHz)	138.88	138.88	138.88
Number of scans	128	128	128
Recycle delay (s)	13	15	15
Spinning rate (kHz)	14	14	14
^{1}H Hartmann-Hahn matching field (kHz)	50	50	50
^{13}C Hartmann-Hahn matching field (kHz)	50	50	50
Contact time (ms)	5	7	9
^{1}H π/2 pulse width (μs)	2.5	2.5	2.5
^{1}H SPINAL-64 decoupling field (kHz)	100	100	100

Table S4. Experimental details for the acquisition of the $^{2}H\{^{1}H\}$ QCPMG ssNMR spectra

	All Others	Temperature (K)	Recycle Delay (s)	Number of Echoes
B_0 (T)	18.8	295.5	180/90[a]	94/89[a]
Time domain size	122880	280	90	89
Dwell time (μs)	0.4	260	240	89
Acquisition time (ms)	49.152	250	600	89
Spectral width (kHz)	1250	240	720	89
Number of scans	8	230	720	89
Excitation Length (us)	3	220	840	89
Refocusing Length (us)	3	200	1080	89
Duration of half echo (us)	510			
Dead Time (us)	50			
^{1}H CW decoupling field (kHz)	50			

[a]A 180 s recycle delay and 94 echoes were used during cooling and a 90 s recycle delay and 89 echoes were used during heating.

Table S5. Crystal data and structure refinement parameters for Compound **1** determined by laboratory-based single crystal X-ray diffraction.

Identification code	mo_DPJB290_0m	mo_DPJB200_0m	mo_DPJB290-Re_0m
Empirical formula	$C_{28}H_{20}N_2OS$	$C_{28}H_{20}N_2OS$	$C_{28}H_{20}N_2OS$
Formula weight	432.52	432.52	432.52
Temperature/K	290	200	290
Crystal system	triclinic	triclinic	triclinic
Space group	$P\bar{1}$	$P\bar{1}$	$P\bar{1}$
a/Å	9.0482(7)	9.3165(13)	9.0383(18)
b/Å	11.2757(9)	9.7354(12)	11.265(2)
c/Å	11.5271(9)	11.7977(15)	11.502(2)
α/°	93.629(3)	76.334(4)	93.570(6)
β/°	99.053(2)	85.051(4)	99.044(6)
γ/°	112.116(2)	87.674(4)	112.215(6)
Volume/Å^3	1066.25(15)	1035.7(2)	1061.2(4)
Z	2	2	2
ρ_{calc}g/cm^3	1.347	1.387	1.354
μ/mm^{-1}	0.176	0.181	0.177
F(000)	452.0	452.0	452.0
Crystal size/mm^3	0.3 × 0.25 × 0.2	0.3 × 0.25 × 0.2	0.3 × 0.25 × 0.2
Radiation	MoK_α	MoK_α	MoK_α
2Θ range for data collection/°	3.61 to 50	3.564 to 49.998	3.618 to 49.998
Index ranges	$-10 \le h \le 10$, $-13 \le k \le 13$, $-13 \le l \le 13$	$-11 \le h \le 11$, $-11 \le k \le 11$, $-13 \le l \le 14$	$-9 \le h \le 10$, $-13 \le k \le 13$, $-13 \le l \le 13$
Reflections collected	11795	13903	16204
Independent reflections	3689 [R_{int} = 0.0273, R_{sigma} = 0.0297]	3588 [R_{int} = 0.0545, R_{sigma} = 0.0463]	3595 [R_{int} = 0.0658, R_{sigma} = 0.0526]
Data/restraints/parameters	3689/0/290	3588/0/290	3595/0/290
Goodness-of-fit on F^2	1.058	1.035	1.080
Final R indexes [I>=2σ (I)]	R_1 = 0.0480, wR_2 = 0.1132	R_1 = 0.0484, wR_2 = 0.1104	R_1 = 0.0791, wR_2 = 0.1534
Final R indexes [all data]	R_1 = 0.0614, wR_2 = 0.1188	R_1 = 0.0649, wR_2 = 0.1184	R_1 = 0.1098, wR_2 = 0.1640
Largest diff. peak/hole / e Å^{-3}	0.44/-0.37	0.26/-0.29	0.39/-0.36
CCDC Number	2406385	2406386	2406424

References

[1] Aguilar-Granda, A.; Pérez-Estrada, S.; Roa, A. E.; Rodríguez-Hernández, J.; Hernández-Ortega, S.; Rodríguez, M.; Rodríguez-Molina, B. Synthesis of a carbazole-[pi]-carbazole molecular rotor with fast solid state intramolecular dynamics and crystallization-induced emission. *Cryst. Growth Des*. **2016**, *16*, 3435-3442.

[2] Belmonte-Vázquez, J. L.; Hernández-Morales, E. A.; Hernández, F.; García-González, M. C.; Miranda, L. D.; Crespo-Otero, R.; Rodríguez-Molina, B. Asymmetric dual-state emitters featuring thiazole acceptors. *Eur. J. Org. Chem*. **2022**, e202200372.

[3] *APEX3*, v2015.52; Bruker AXS Inc.: Madison, WI, 2015.

[4] *SAINT*, v8.34A; Bruker AXS Inc.: Madison, WI, 2013.

[5] Blessing, R. H. An empirical correction for absorption anisotropy. *Acta Crystallogr. Sect. A: Found. Crystallogr*. **1995**, *51*, 33–38.

[6] Dolomanov, O. V.; Bourhis, L. J.; Gildea, R. J.; Howard, J. A. K.; Puschmann, H. OLEX2: a complete structure solution, refinement and analysis program. *J. Appl. Crystallogr*. **2009**, *42*, 339-341.

[7] Sheldrick, G. M. SHELXT - Integrated space-group and crystal-structure determination. *Acta Crystallogr. Sect. A: Found. Adv.* **2015**, *71*, 3–8.

[8] Spek, A. L. Single-crystal structure validation with the program PLATON. *J. Appl. Crystallogr*. **2003**, *36*, 7–13.

[9] Nardelli, M. PARST95—an update to PARST: a system of Fortran routines for calculating molecular structure parameters from the results of crystal structure analyses. *J. Appl. Cryst*. **1995**, *28*, 659.

[10] *Persistence of Vision Raytracer* (POV-Ray), version 3.6; Persistence of Vision Pty Ltd.: Victoria, Australia, 2004.

[11] Oliver, W. C.; Pharr, G. M. An improved technique for determining hardness and elastic modulus using load and displacement sensing indentation experiments. *J. Mater. Res*. **1992**, *7*, 1564–1583.

[12] Fischer-Cripps, A. C. Nanoindentation testing: nanoindentation 21–37 (Springer, 2011).

[13] Peersen, O. B.; Wu, X. L.; Kustanovich, I.; Smith, S. O. Variable-amplitude cross-polarization MAS NMR. *J. Magn. Reson. Ser. A* **1993**, *104*, 334–339.

[14] Metz, G.; Wu, X. L.; Smith, S. O. Ramped-amplitude cross polarization in magic-angle spinning NMR. *J. Magn. Reson. Ser. A1* **1994**, *110*, 219–227.

[15] Schaefer, J.; Stejskal, E. O. Carbon-13 nuclear magnetic resonance of polymers spinning at the magic angle. *J. Am. Chem. Soc.* **1976**, *98*, 1031–1032.

[16] Pines, A.; Gibby, M. G.; Waugh, J. S. Proton-enhanced nuclear induction spectroscopy ^{13}C chemical shielding anisotropy in some organic solids. *Chem. Phys. Lett.* **1972**, *15*, 373–376.

[17] Pines, A.; Gibby, M. G.; Waugh, J. S. Proton-enhanced NMR of dilute spins in solids. *J. Chem. Phys.* **1973**, *59*, 569–590.

[18] Taylor, R. E. ^{13}C CP/MAS: application to glycine. *Concepts Magn. Reson.* **2004**, *22A*, 79–89.

[19] Guan, X.; Stark, R. E. A general protocol for temperature calibration of MAS NMR probes at arbitrary spinning speeds. *Solid State Nucl. Magn. Reson.* **2010**, *38*, 74–76.

[20] Larsen, F. H.; Jakobsen, H. J.; Ellis, P. D.; Niels, N. C. Sensitivity-enhanced quadrupolar-echo NMR of half-integer quadrupolar nuclei. Magnitudes and relative

orientation of chemical shielding and quadrupolar coupling tensors. *J. Phys. Chem. A* **1997**, *101*, 8597–8606.
[21] *Materials Studio*, version 8.0.0.843; Dassault systèmes: San Diego, CA, 2014.
[22] Mayo, S. L.; Olafson, B. D.; Goddard, W. A. DREIDING: a generic force field for molecular simulations. *J. Phys. Chem*. **1990**, *94*, 8897– 8909.
[23] Rappé A. K.; Goddard W. A. Charge equilibration for molecular dynamics simulations. *J. Phys. Chem*. **1991**, *95*, 3358–3363.
[24] Nosé, S. A unified formulation of the constant temperature molecular-dynamics methods. *J. Chem. Phys*. **1984**, *81*, 511–519.
[25] Berendsen, H. J. C.; Postma, J. P. M.; van Gunsteren, W. F.; DiNola, A.; Haak, J. R. Molecular dynamics with coupling to an external bath. *J. Chem. Phys*., **1984**, *81*, 3684-3690.
[26] Delley, B. An all-electron numerical method for solving the local density functional for polyatomic molecules. *J. Chem. Phys.* **1990**, *92*, 508-517.
[27] Zhao, Y.; Truhlar, D. G. A new local density functional for main-group thermochemistry, transition metal bonding, thermochemical kinetics, and noncovalent interactions. *J. Chem.Phys*., **2006**, *125*, 194101.
[28] Delley, B. Ground-state enthalpies: evaluation of electronic structure approaches with emphasis on the density functional method. *J. Phys. Chem. A*, **2006**, *110*, 13632.
[29] *Gaussian 16*, Revision C.01, Frisch, M. J.; Trucks, G. W.; Schlegel, H. B.; Scuseria, G. E.; Robb, M. A.; Cheeseman, J. R.; Scalmani, G.; Barone, V.; Petersson, G. A.; Nakatsuji, H.; Li, X.; Caricato, M.; Marenich, A. V.; Bloino, J.; Janesko, B. G.; Gomperts, R.; Mennucci, B.; Hratchian, H. P.; Ortiz, J. V.; Izmaylov, A. F.; Sonnenberg, J. L.; Williams-Young, D.; Ding, F.; Lipparini, F.; Egidi, F.; Goings, J.; Peng, B.; Petrone, A.; Henderson, T.; Ranasinghe, D.; Zakrzewski, V. G.; Gao, J.; Rega, N.; Zheng, G.; Liang, W.; Hada, M.; Ehara, M.; Toyota, K.; Fukuda, R.; Hasegawa, J.; Ishida, M.; Nakajima, T.; Honda, Y.; Kitao, O.; Nakai, H.; Vreven, T.; Throssell, K.; Montgomery, J. A., Jr.; Peralta, J. E.; Ogliaro, F.; Bearpark, M. J.; Heyd, J. J.; Brothers, E. N.; Kudin, K. N.; Staroverov, V. N.; Keith, T. A.; Kobayashi, R.; Normand, J.; Raghavachari, K.; Rendell, A. P.; Burant, J. C.; Iyengar, S. S.; Tomasi, J.; Cossi, M.; Millam, J. M.; Klene, M.; Adamo, C.; Cammi, R.; Ochterski, J. W.; Martin, R. L.; Morokuma, K.; Farkas, O.; Foresman, J. B.; Fox, D. J. Gaussian, Inc., Wallingford CT, 2016.
[30] Zhao, Y.; Truhlar, D. G. The M06 suite of density functionals for main group thermochemistry, thermochemical kinetics, noncovalent interactions, excited states, and transition elements: two new functionals and systematic testing of four M06-class functionals and 12 other functionals. *Theo. Chem. Acc.* **2008**, *120*, 215-241.
[31] Weigend, F.; Ahlrichs, R. Balanced basis sets of split valence, triple zeta valence and quadruple zeta valence quality for H to Rn: design and assessment of accuracy. *Phys. Chem. Chem. Phys*., **2005**, 7, 3297-3305.
